# Synthesis and evaluation of novel 2,4-disubstituted arylthiazoles against *T. brucei*†
†Electronic supplementary information (ESI) available. See DOI: 10.1039/c9md00478e


**DOI:** 10.1039/c9md00478e

**Published:** 2019-12-19

**Authors:** Markos-Orestis Georgiadis, Violeta Kourbeli, Ioannis P. Papanastasiou, Andrew Tsotinis, Martin C. Taylor, John M. Kelly

**Affiliations:** a Division of Pharmaceutical Chemistry , Department of Pharmacy , School of Health Sciences , National and Kapodistrian University of Athens , Panepistimioupoli-Zografou , 157 84 Athens , Greece . Email: papanastasiou@pharm.uoa.gr; b Department of Pathogen Molecular Biology , London School of Hygiene and Tropical Medicine , Keppel Street , London WC1 E7HT , UK

## Abstract

The design, synthesis and pharmacological evaluation of the 4-substituted-2-[3-(adamant-1-yl)-4-fluorophenyl]thiazoles **1a–j**, the 4-substituted-2-[4-(adamant-1-yl)phenyl]thiazoles **2a–h**, the 2-substituted-4-[4-(adamant-1-yl)phenyl]thiazoles **3a–e**, the *N*-substituted 2-phenylthiazol-4-ethylamides **4a**, **b** and the *N*-substituted 4-phenylthiazol-2-ethylamides **4c**, **d** is described. Compounds **1a** and **2a** exhibit trypanocidal activity in the range of IC_50_ = 0.42 μM and IC_50_ = 0.80 μM, respectively. Both of these derivatives bear a lipophilic end, which consists of a 4-(1-adamantyl) phenyl or a 3-(1-adamantyl)phenyl moiety, a 1,3-thiazole ring and a functional end, which comprises of an alkylamine and can be considered as promising candidates for the treatment of *Trypanosoma brucei* infections.

## Abstract 2

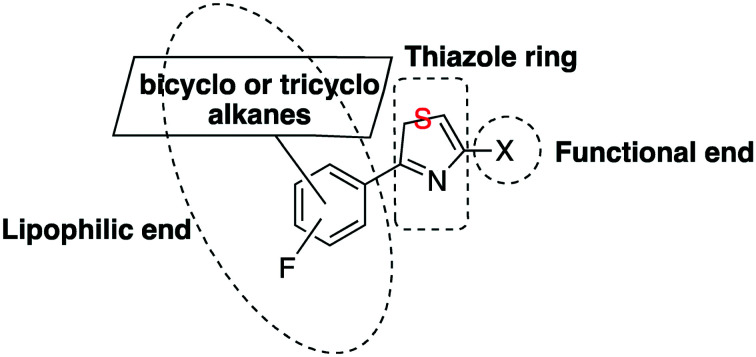
2-{2-[3-(1-Adamantyl)-4-fluorophenyl]thiazol-4-yl}ethan-1-amine (**1a**) and 2-{2-[4-(1-adamantyl)phenyl]thiazol-4-yl}ethan-1-amine (**2a**) exhibit activity against *T. brucei* in the range of IC_50_ = 0.42 μM and IC_50_ = 0.80 μM, respectively.

## Introduction

The African sleeping sickness and the Chagas disease are two of the major neglected tropical diseases (NTDs). The trypanosomiases are vector-borne parasitic infections caused by flagellated protozoa of the class Kinetoplastida.^1^ There are two species of human-infectious trypanosomes, *Trypanosoma brucei*, that causes human African trypanosomiasis (HAT), and *Trypanosoma cruzi*, which is responsible for the Chagas disease. HAT is prevalent in sub-Saharan Africa, transmitted by the bite of a tsetse fly infected with one of the two subspecies, *Trypanosoma brucei gambiense* or *Trypanosoma brucei rhodesiense*. The Chagas disease is spread predominantly in Latin and Central America by *Triatominae* bugs infected with *T. cruzi*.^2^,^3^ Trypanosomiases, as with other NTDs, are becoming public health problems in non-endemic countries, as a result of travel and migration. New drugs are urgently required, as those that are currently available are characterized by side-effects and treatment failures.^4^,^5^

**Fig. 1:**
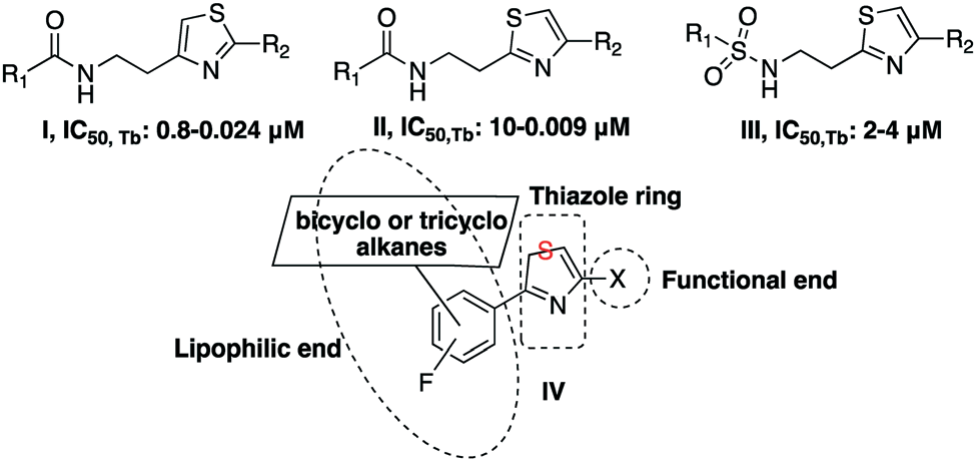
General type scaffolds with trypanocidal activity.

Various initiatives^6^–^8^ have led to the discovery of promiscuous trypanocidal derivatives from phenotypic high-throughput screening of a number of compound libraries. These have been further refined and optimized to enhance drug-like properties. The Walter and Eliza Hall Institute (WEHI), in partnership with the Drugs for Neglected Diseases *initiative* (DND*i*), and the Genomics Institute of the Novartis Research Foundation (GNF) have described the amide and urea derivatives of thiazolethylamines **I**, **II** and sulfonamides **III**, shown in Fig. 1, as potent trypanocidals.^6^,^7^

Based on these findings and our involvement in the adamantane chemistry,^9^–^20^ we report herein on the chemistry and biology of thiazole derivatives of the general type scaffold **IV**. The thiazole moiety is an important pharmacophore in many compounds used against several tropical infectious diseases.^21^

**Fig. 2:**
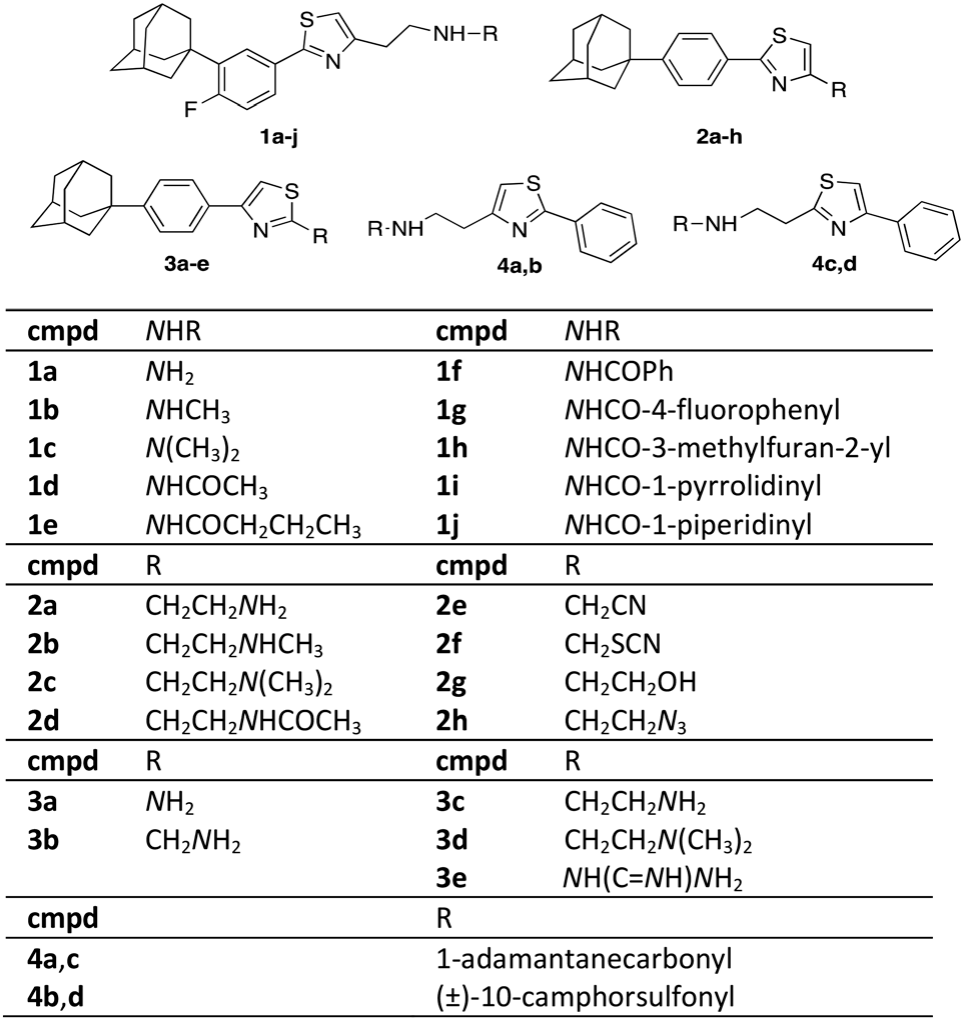
Novel thiazole derivatives **1a–j**, **2a–h**, **3a–e**, **4a–d**.

Scaffold **IV** includes a 1,3-thiazole moiety, which is 2,4-disubstituted. One substituent is the lipophilic end of the scaffold, which consists of a phenyl ring bearing fluoro- and 1-adamantyl-functionalities. The 4-(1-adamantyl)phenyl substituent has been proven to be well tolerated and is endowed with trypanocidal properties.^22^ The thiazole ring bears a variety of functional groups (Fig. 2).

2-Phenylthiazol-4-ethylamines **1a–d** and **2a–d** share the same structural features, apart from the relative position of the 1-adamantyl core and the addition of a fluoro-substituent in series **1**. Fluorine alters the biophysical and chemical properties, such as lipophilicity, acidity, as well as the reactivity and conformation of the substituted derivatives.^23^ In 2018, 18 out of the 38 small drug molecules, that were approved by the FDA, contain a fluorine atom.^24^,^25^ Derivatives **3** differ in the thiazole moiety compared to adducts **1** and **2**. The 2,4-substituents of the thiazoles **2a**, **c** have their positions switched in derivatives **3c**, **d**. The functionalization of the amino-end of congeners **1** involves various amide (aromatic and non-aromatic) and urea substituents. In adducts **2**, the polar heads were translocated to the functional end of the general type scaffold **IV**. The length of the side chain of derivatives **2e**, **2g** and **3e** was kept at the distance of three atoms (2C and 1N and *vice versa*), which in the derivatives **I**, **II** and **III** was found to be the optimal length for enhanced trypanocidal potency.^6^–^8^ The length of the R group is different in adducts **2f**, **h** and **3a**, **b**. 2-Aminothiazole (adduct **3a**), is a frequent-hitting fragment in biophysical binding assays.^26^ Moreover, an analogous thiazole guanidinium system of derivative **3e** has been used as a substitute for other aromatic rings improving biological activity.^27^

The relative position of the adamantane cage, the phenyl ring and the thiazole moiety was altered in derivatives **4a**, **c**. Compounds **4a**, **c** bear the same thiazole ring substituents as derivatives **2** and **3**. Additionally, the adamantane core was replaced in the camphor skeleton in adducts **4b**, **4**. The latter molecules are sulfonamides in alignment with the scaffolds of compounds **III**.^7^

## Results and discussion

### Synthesis

**Scheme 1:**
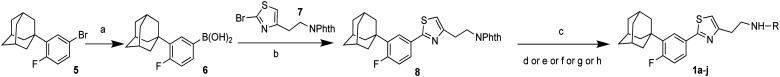
Reagents and conditions: (a) i. *n*-BuLi, THF, –78 °C, 20 min, ii. (i-PrO)_3_B, r.t. 18 h, 90%; (b) Pd(PPh_3_)_4_, Na_2_CO_3_, anh. toluene, 80 °C, 18 h, 80%; (c) H_2_NNH_2_.H_2_O, EtOH, reflux, 1 h; (d) i. ClCOOEt, NEt_3_, THF, r.t. 18 h, ii. LiAlH_4_, THF, reflux 4 h, iii. EtOH, H_2_O, NaOH 10%, 0 °C, 88% from **8**; (e) MeONa, CH_3_COOH, HCHO 38% in H_2_O, NaBH_3_CN, MeOH, r.t. 18 h, 75% from **8**. (f) Benzoyl chloride (**1f**: 96% from **8**) or 4-fluorobenzoyl chloride (**1g**: 93% from **8**) or 1-piperidinecarbonyl chloride (**1j**: 36% from **8**) or 1-pyrrolidinecarbonyl chloride (**1i**: 46% from **8**), Et_3_N, EtOAc, r.t. 18 h; (g) acetic anhydride (**1d**: 84% from **8**) or butyric anhydride (**1e**: 92% for **8**), Et_3_N, EtOAc, r.t. 48 h; (h) 3-methyl-furoic acid, EDCI, HOBt, DMAP, DMF/DCM, 45 °C, 18 h, 29% from **8**.

The 4-substituted-2[3-(adamant-1-yl)-4-fluorophenyl]thiazoles **1a–j** were synthesized as shown in Scheme 1. As starting material, for the synthesis of thiazoles **1a–j**, the (3-adamant-1-yl)-(4-fluorophenyl)boronic acid (**6**) was used. The reported method for the preparation of the boronic acid **6** (ref. 28) has been modified, by changing the reaction times. The synthetic route involved a Suzuki–Miyaura palladium-catalyzed coupling between the boronic acid **6** and the 2-thiazole bromide **7** (ref. 6) to provide the phthalimide protected adamantane derivative **8**. The hydrazinolysis of phthalimide derivative **8** led to the deprotected parent compound 2-{2-[3-(adamant-1-yl)-4-fluorophenyl]thiazol-4-yl}ethan-1-amine (**1a**), which was subsequently methylated, dimethylated,^29^ acylated and carbamoylated to deliver adducts **1b–j**, *via* the procedures shown in Scheme 1.

**Scheme 2:**
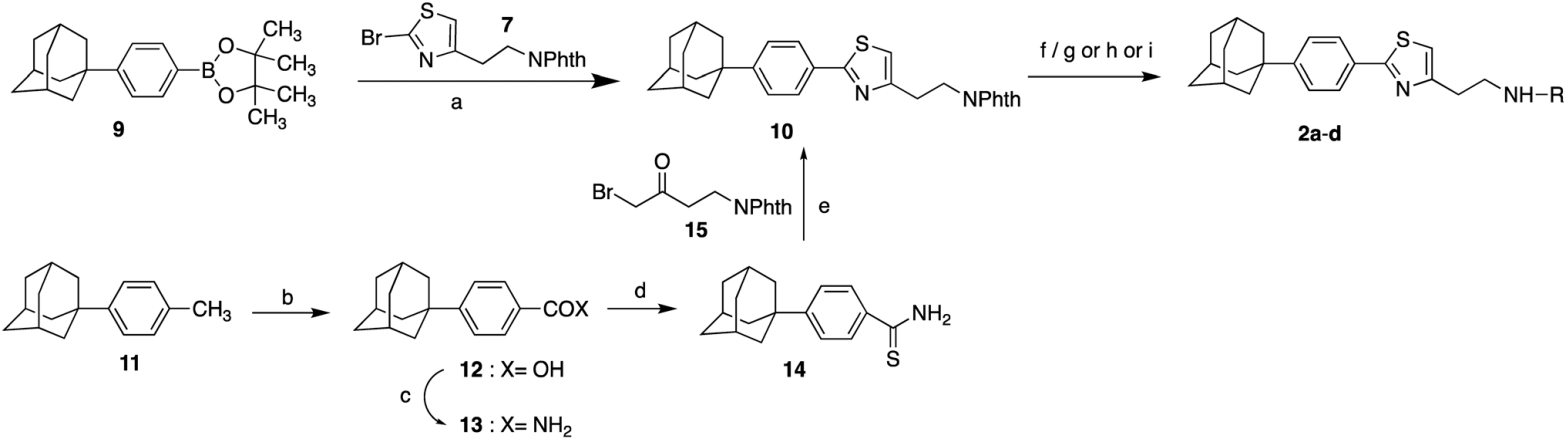
Reagents and conditions: (a) Pd(PPh_3_)_4_, Na_2_CO_3_, anh. toluene, 80 °C, 18 h, 87%; (b) O_2_, NaBr, Mn(OAc)_2_, Co(OAc)_2_, AcOH/dioxane, 90 °C, 6 h, 89%; (c) i. SOCl_2_, 65 °C, 45 min, ii. aq. NH_3_, 25%, r.t. 1 h, 96%; (d) Lawesson's reagent, dioxane, 110 °C, 2 h, 50%; (e) i-PrOH, autoclave, 120 °C, 18 h, 65%; (f) H_2_NNH_2_·H_2_O, EtOH, reflux 1 h; (g) acetic anhydride, Et_3_N_,_ EtOAc, r.t. 48 h, 53% from **10**; (h) i. ClCOOEt, NEt_3_, THF, r.t. 18 h, ii. LiAlH_4_, THF, reflux 4 h, iii. EtOH, H_2_O, NaOH 10%, 0 °C, 54% from **10**; (i) MeONa, CH_3_COOH, HCHO 38% in H_2_O, NaBH_3_CN, MeOH, r.t. 18 h, 85% from **10**.

The synthesis of the 4-substituted-2[4-(adamant-1-yl)phenyl]thiazoles **2a–d** was realized following two synthetic pathways, as illustrated in Scheme 2. The key-compound for the preparation of the thiazoles **2a–h**, the 2-{2-[(2-(adamant-1-yl)phenyl)thiazol-4-yl]ethyl}isoindoline-1,3-dione (**10**) was obtained *via* two different synthetic routes. The first involves a Suzuki–Miyaura^30^ palladium-catalysed coupling between the 4,4,5,5-tetramethyl-2-[4-(adamant-1-yl)phenyl]-2*H*-1,3,2-dioxaborolane (**9**)^31^ and the 2-thiazole bromide **7**, which led to the protected precursor **10**. The second synthetic approach, towards the thiazole adduct **10**, was based on the Hantzsch condensation^32^ of thiobenzamide **14** with the α-bromoketone **15**.^6^ Our lab has previously published the preparation of the 4-(adamant-1-yl)benzoic acid (**12**),^31^ which was now obtained by a different transition metal ion catalyzed oxidation of 1-(4-tolyl)adamantane (**11**).^33^ The reported method of oxidation^34^ was modified as the reaction mixture was bubbled with oxygen gas and heated at 105 °C for 6 h. The benzoic acid **12** was subsequently converted to the corresponding benzamide **13** and the thiobenzamide **14**. Comparing the two methods, the first involves 5 steps (17% total yield), while the second 6 steps (25% total yield), a more facile work-up and cheaper reagents. The parent thiazole **2a** was methylated, dimethylated and acetylated to the respective congeners **2b–d**, as previously shown.

**Scheme 3:**
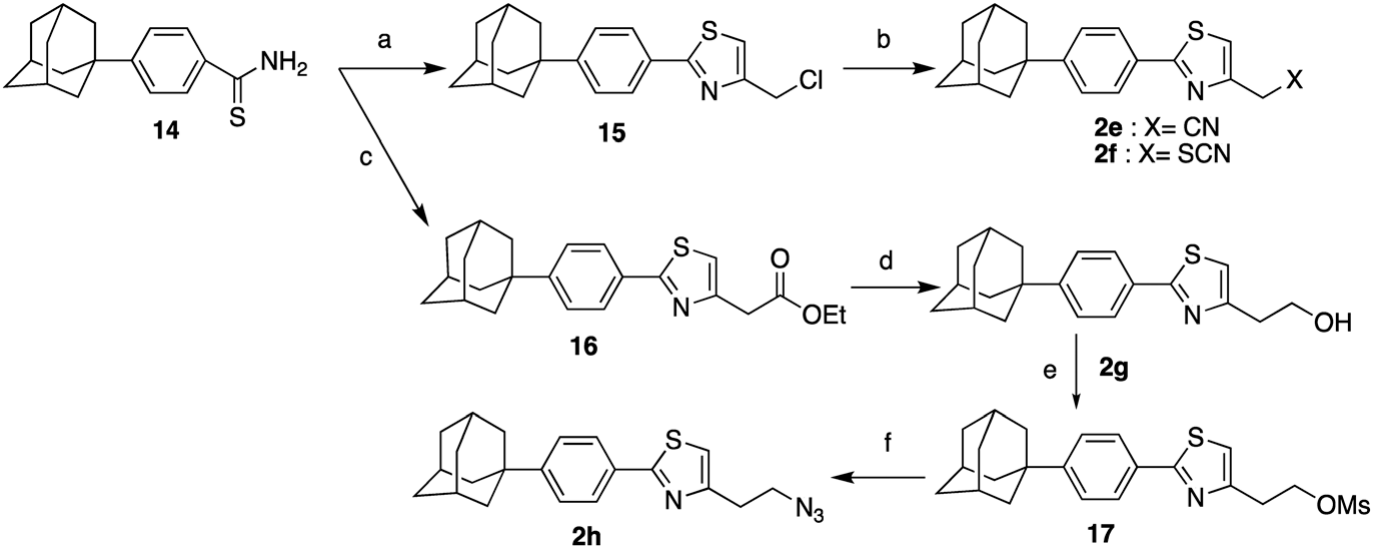
Reagents and conditions: (a) 1,3-dichloroacetone, acetone, reflux, 18 h, 75%; (b) KCN, anh. DMF, 60 °C, 36 h (**2e**: 41%) or KSCN, EtOH, 45 °C, 18 h (**2f**: 67%); (c) 4-chloroacetoacetate, i-PrOH, autoclave, 120 °C, 18 h, 92%; (d) i. LiAlH_4_, THF, r.t. 2 h, ii. EtOH, H_2_O, NaOH 10%, 0 °C, 80%; (e) MsCl, Et_3_N, DCM, 0 °C then r.t. 18 h, 95%; (f) NaN_3_, anh. DMF, 60 °C, 2 h, 65%.

The functionalized thiazoles **2e–h** were obtained by the route shown in Scheme 3. The thiobenzamide **14** was condensed with 1,3-dichloroacetone and 4-chloroacetoacetate, under Hantzsch reaction conditions, to give the chloromethylthiazole **15** and the thiazolethyl acetate **16**, respectively. Treatment of the choromethylthiazole **15** with KCN or KSCN led to the respective cyanide **2e** and the thiocyanide **2f**. The thiazolethyl acetate **16** was reduced to the corresponding alcohol **2g**, which was then converted to the azide **2h**, *via* activation of the methanesulphonyl derivative **17**.

**Scheme 4:**
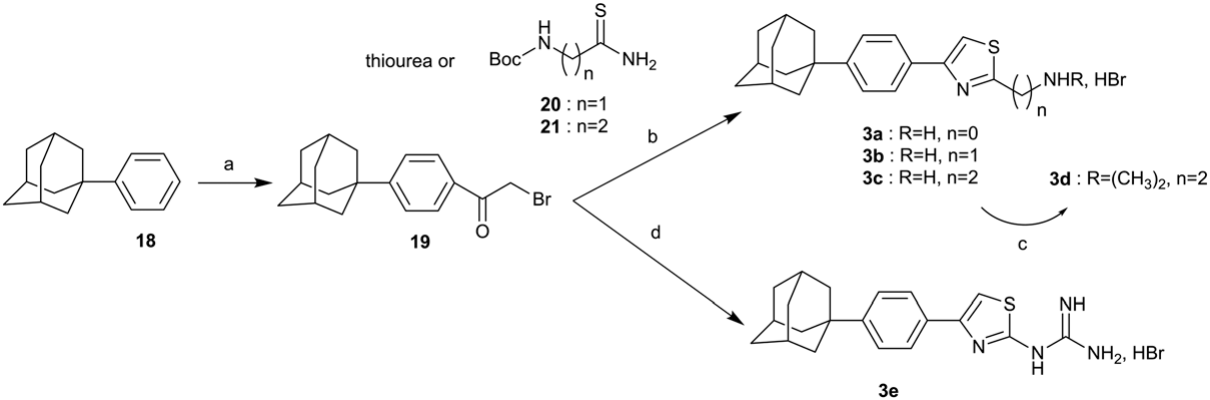
Reagents and conditions: (a) BrCOCH_2_Br, AlCl_3_, DCM, –10 °C then r.t. 18 h, 57%; (b) appropriate thiobenzamide **20** (**3b**: 36%), 21 (**3c**: 85%), i-PrOH, autoclave, 120 °C 18 h or thiourea (**3a**: 88%), EtOH, reflux, 18 h; (c) MeONa, CH_3_COOH, HCHO 38% in H_2_O, NaBH_3_CN, MeOH, r.t. 18 h, 85%; (d) guanylthiourea, EtOH, reflux, 18 h, 89%.

The synthesis of the 2-substituted-4-{4-(adamant-1-yl)phenyl}thiazoles **3a–d** and the guanidyl derivative **3e**, is shown in Scheme 4. (1-Phenyl)adamantane (**18**)^4^ was acylated under Friedel–Crafts reaction conditions^36^ to deliver the corresponding α-bromoketone **19**, which *via* a Hantzsch condensation with the appropriate reagent, thiourea,^37^ thioamides **20**,^38^**21** (ref. 7) and guanylthiourea provided the desired thiazoles **3a–c** and **3e**, respectively. The dimethylthiazole **3d** was prepared from the parent thiazole **3c**, as shown before.

**Scheme 5:**
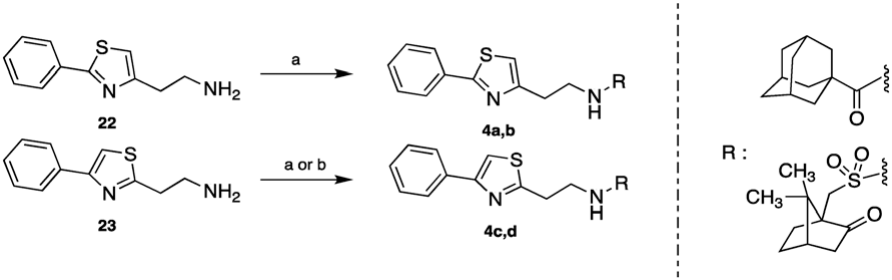
Reagents and conditions: (a) appropriate chloride NEt_3_, DCM or THF, r.t. 18 h, (**4a**: 49%, **4b**: 50%, **4d**: 81%); (b) 1-adamantanecarboxylic acid HBTU, DIPEA, DCM/DMF, r.t. 24 h, 94%.

The 1-adamantylcarbonylamides **4a**, **c** and the (±)-10-camphorsulfonyl amides **4b**, **d** were obtained upon coupling the commercially available 1-adamantylcarboxylic acid and (±)-10-camphorsulfonyl chloride with the 2-phenylthiazol-4-ethylamine (**22**)^6^ and the 4-phenylthiazol-2-ethylamine (**23**),^7^ respectively. The acid reacted in the presence of the coupling reagent HBTU, while the chlorides reacted without the aid of any activating reagent (Scheme 5).

### Pharmacology

The 27 new thiazole derivatives were tested for their activity against the bloodstream form *Trypanosoma brucei* and the results are shown in Table 1.

**Table 1:** Screening of the new thiazole derivatives against *T. brucei*

Cmpd	*T. brucei* IC_50_^*a*^ (μM)	*T. brucei* IC_90_^*a*^ (μM)	L6 cells IC_50_^*a*^ (μM)	S.I.^*b*^
**1a**	0.42 ± 0.01	0.56 ± 0.01	1.05 ± 0.23	2.5
**1b**	0.90 ± 0.01	1.10 ± 0.01	2.01 ± 0.32	2.2
**1c**	0.79 ± 0.02	1.03 ± 0.01	1.53 ± 0.09	1.9
**1d**	15.3 ± 0.2	18.1 ± 0.2	—	—
**1e**	>25	—	—	—
**1f**	>20	—	—	—
**1g**	>20	—	—	—
**1h**	>20	—	—	—
**1i**	>20	—	—	—
**1j**	10.7 ± 0.3	12.6 ± 0.2	<10.30	<1
**2a**	0.80 ± 0.03	1.17 ± 0.01	4.08 ± 0.15	5.1
**2b**	0.59 ± 0.02	0.79 ± 0.01	0.96 ± 0.26	1.6
**2c**	1.27 ± 0.07	1.60 ± 0.22	—	—
**2d**	>20	—	—	—
**2e**	>20	—	—	—
**2f**	∼10	—	—	—
**2g**	∼10	—	—	—
**2h**	>20	—	—	—
**3a**	22.5 ± 0.6	30.5 ± 4.5	13.8 ± 1.6	<1
**3b**	9.76 ± 0.77	12.8 ± 0.2	12.6 ± 0.9	<1
**3c**	2.74 ± 0.29	4.40 ± 0.07	4.16 ± 0.24	1.5
**3d**	1.41 ± 0.09	3.58 ± 0.05	3.19 ± 0.25	2.3
**3e**	∼10	—	—	—
**4a**	12.2 ± 0.8	18.7 ± 0.4	—	—
**4b**	20.6 ± 0.7	31.1 ± 0.3	—	—
**4c**	9.82 ± 0.22	13.1 ± 0.2	—	—
**4d**	23.8 ± 1.1	31.5 ± 0.6	—	—

^*a*^IC_50_ and IC_90_; concentration that inhibits growth by 50% and 90%.

^*b*^S.I.; selectivity index, the ratio of IC_50_ values obtained with L6 cells and *T. brucei* respectively.

It is apparent from the test results that the ethylamines **1a–c** exhibit the highest activity among the new 2,4-disubstituted arylthiazoles. Bulkier substituents than the methyl group at the amino end have a negative impact on trypanocidal activity. Amido adducts (alkyl **1d** and **1c**, the aromatic **1f** and **1g** and the heteroaromatic **1h**) and the ureido derivatives, **1i** and **1j**, have a non-significant activity. The same pattern is also observed in the **2** series, as compounds **2a–c** are *ca.* 20 times more active than their acetamido congener **2d**. Comparing series **1** and **2**, it becomes apparent that the fluorine substitution has little positive effect on the activity. The dimethylamino isomeric thiazoles **2c** and **3d** present almost the same potency, while the nor-derivatives, the isomeric thiazoles **2a** and **3c**, show a substantial difference in potency. The 2-phenylthiazol-4-ethylamines **1a**, **c** and **2a**, **c** seem to be, in general, more potent than their isomeric 4-phenylthiazol-2-ethylamines **3c** and **3d**. The decrease of the length of the side chain does not enhance activity. Methanamine **3b** bears two atoms (carbon and nitrogen) in its side chain and is twice as potent as the 2-aminothiazole **3a**, which has only one nitrogen atom. The polar functionalization of the side chain did not improve the trypanocidal activity. The azido and cyano-tailored derivatives **2e** and **2h** are less potent, and the thiocyanate **2f**, the ethanol **2g** and the guanyl derivative **3e** exhibit modest activity. The change in the relative position of the adamantane cage, the phenyl ring and the thiazole moiety, in adducts **4a** and **4c**, did not lead to activity enhancement. Last, the replacement of the adamantane skeleton by the camphorsulfonyl moiety in derivatives **4b** and **4d** has not led to antitrypanosomal enhancement. The 2,4-disubstituted arylthiazole adamantane derivatives, the ethylamines **1a–c** and **2a–c**, present a notable pharmacological profile, which merits further investigation in terms of activity and toxicity. These findings suggest that an aliphatic amine moiety at the side chain is mandatory to achieve notable trypanocidal activity. This amine group is positively charged at the cytosolic pH, which is not the case for all the other polar heads tested. The presence of this particular group might also enhance the cellular accumulation into the protozoa, as it is reported in the case of bacteria.^39^,^40^ Thus, the ethylamines **1a–c** and **2a–c** seem to exhibit promising trypanocidal properties, although further optimisation will be necessary to reduce their cytotoxicity and to develop a more drug-like profile.

## Conclusions

In this work, we describe the synthesis of a new series of aromatic 2,4-disubstituted 1,3-thiazole analogues with trypanocidal potency. Among their congeners, the 2-phenylthiazol-4-ethylamines **1a–c** and **2a–c** presented the most significant trypanocidal activity against *T. brucei*. Analogues **1a** and **2a** exhibit antitrypanosomal activity in the range of IC_50_ = 0.42 μM and IC_50_ = 0.80 μM, respectively. Primary amine **2a** is less potent than its congener **1a**, but exhibits higher selectivity, which is a promising perspective for designing new trypanocidals in the future. Both of these classes of derivatives bear a lipophilic end, which consists of a 4-(1-adamantyl)phenyl or a 3-(1-adamantyl)phenyl moiety, a 1,3-thiazole ring and a functional end, which comprises of an alkylamine. The addition of the adamantane ring into the scaffold of the thiazole reference compounds^6^,^7^ has not improved their pharmacological profile, in terms of activity and toxicity. On the other hand, the new congeners exhibit promising trypanocidal properties that merit further investigation. These tailored-made structural modifications will be implemented in the future in the design of trypanocidal agents.

## Experimental part

### Biology

#### Cytotoxic activity against rat skeletal myoblast L6 cells

Cytotoxicity against mammalian cells was assessed using microtitre plates. Briefly, L6 cells (a rat skeletal muscle line) were seeded at 1 × 10^4^ mL^–1^ in 200 μL of growth medium containing 7 different compound concentrations in a range previously established to encompass both the IC_50_ and IC_90_ values. The plates were incubated for 6 days at 37 °C and 20 μL Alamar Blue (Biosource UK Ltd) was then added to each well. After an additional 8 hours incubation, the fluorescence was determined using a FLUOstar Omega fluorescent plate reader (BMG Labtech). Inhibition of growth was calculated by comparison with control values and IC_50_ and IC_90_ values were determined in triplicate using linear regression analysis.

#### *Trypanosoma brucei* culturing and drug testing

Bloodstream form *T. brucei* (strain 427) were cultured at 37 °C in modified Iscove's medium. Trypanocidal activity was assessed by growing parasites in microtiter plates in the presence of various drug concentrations. Parasites were seeded at 0.25 × 10^5^ mL^–1^ in 200 μL of growth medium containing 7 different compound concentrations in a range previously established to encompass both the IC_50_ and IC_90_ values. The plates were incubated for 48 hours at 37 °C and 20 μL Alamar Blue was then added to each well. After an additional overnight incubation, the fluorescence was determined. Inhibition of growth was calculated by comparison with control values and IC_50_ and IC_90_ values were determined in triplicate using linear regression analysis.

#### Synthetic procedures

All chemicals and solvents were obtained from commercial suppliers and used without further purification. Reactions were monitored by thin layer chromatography. Melting points were determined on a Büchi 530 apparatus and are uncorrected. Infrared (IR) spectra were recorded on a Perkin-Elmer 833 spectrophotometer. ^1^H-NMR spectra recorded on a Bruker DRX 400 (400 MHz) spectrometer and ^13^C-NMR spectra were taken at 50 MHz on Bruker AC 200 (200 MHz) spectrometer and at 150 MHz on Bruker Avance 600 spectrometer (600 MHz). All NMR spectra were taken in deuterochloroform or hexadeuterodimethyl sulfoxide and the chemical shifts are reported in ppm. Elemental analyses (C, H, N) were carried out by the Institute of Chemical Biology, NHRF, Greece and the results obtained had a maximum deviation of ±0.4% from the theoretical value.

#### 3-{(1-Tricyclo[3.3.1.1^3,7^]decyl)-4-fluorophenyl}boronic acid (**6**)

*n*-BuLi (4 mL,1.6 M in hexanes, 6.4 mmol) was added in one portion to a stirred solution of the bromide **5** (ref. 28) (1.25 g, 4.04 mmol) in anhydrous THF (20 mL), at –73 °C, under an argon atmosphere. The mixture was then stirred at –80 °C for 25 min prior to addition of (i-PrO)_3_B (3 mL, 12.1 mmol). The reaction mixture was stirred for 35 min at the same temperature and subsequently at ambient temperature overnight. Next, dilute HCl (20 mL) was added dropwise at 0 °C, the mixture was stirred for 30 min at room temperature and then extracted with EtOAc (3 × 20 mL). The combined organic layers were washed with water, dried over Na_2_SO_4_ and the solvent evaporated under reduced pressure. The residue was crystallized from *n*-hexane to give compound **6** (1.1 g, 90%) as a white solid, which was used directly in the next step. ^1^H NMR (400 MHz, CDCl_3_) *δ* 8.18 (d, *J* = 8.7 Hz, 1H, 2-Har), 8.03 (dd, *J* = 8.4, 4.2 Hz, 1H, 6-Har), 7.12 (dd, *J* = 8.1, 7.8 Hz, 1H, 5-Har), 2.12 (bs, 3H, 3,5,7-Had), 2.06 (bs, 6H, 2,8,9-Had), 1.79 (br.s, 6H, 4,6,10-Had).

#### 2-{2-[2-((1-Tricyclo[3.3.1.1^3,7^]decyl)-4-fluorophenyl)thiazol-4-yl]ethyl}isoindoline-1,3-dione (**8**)

Argon was bubbled for 20 min through a stirred mixture of boronic acid **6** (250 mg, 0.9 mmol), the 2-thiazole bromide **7** (ref. 6) (161 mg, 0.45 mmol), toluene (5 mL) and Na_2_CO_3_ (2 M, 5 mL). The reaction mixture was then heated to 80 °C, under an argon atmosphere, Pd(PPh_3_)_4_ (107 mg) was added and heating was continued overnight. After cooling, the reaction mixture was extracted with EtOAc (3 × 25 mL) and the combined organics were washed with water dried over Na_2_SO_4_ and the solvent removed *in vacuo*. The residue was purified by column chromatography. Elution with 10–20% EtOAc in hexanes afforded compound **8** as a foamy solid (175 mg, 80%). ^1^H NMR (400 MHz, CDCl_3_) *δ* 7.85–7.79 (m, 2H, 3′,4′-Har), 7.76 (dd, *J* = 7.7, 2.2 Hz, 1H, 2-Har), 7.73–7.68 (m, 2H, 2′,5′-Har), 7.64–7.58 (m, 1H, 6-Har), 6.71 (s, 1H, 5-Hth), 6.96 (dd, *J* = 12.7, 8.3 Hz, 1H, 5-Har), 4.11 (t, *J* = 7.0 Hz, 2H, CH_2_*N*), 3.23 (t, *J* = 7.0 Hz, 2H, CH_2_), 2.10 (br.s, 3H, 3,5,7-Had), 2.04 (bs, 6H, 2,8,9-Had), 1.79 (br.s, 6H, 4,6,10-Had). ^13^C NMR (150 MHz, DMSO-*d*_6_) *δ* 167.2 (C=O), 166.6 (2-Cth), 162.8 (d, *J* = 251.9 Hz, 4-Car), 152.8 (4-Cth), 137.5 (d, *J* = 11.4 Hz, 3-Car), 132.9 (1′,6′-Car), 132.8 (3′,4′-Car), 129.2 (1-Car), 126.2 (d, *J* = 8.4 Hz, 2-Car), 125.1 (6-Car), 122.2 (2′,5′-Car) 117.5 (d, *J* = 25.4, 5-Car), 116.4 (5-Cth), 40.5 (2,8,9-Cad), 38.4 (*N*CH_2_), 36.2 (1-Cad), 36.0 (4,6,10-Cad), 28.8 (CH_2_), 28.2 (3,5,7-Cad). Anal. calcd for C_29_H_27_FN_2_O_2_S: C, 71.58; H, 5.59; N, 5.76 found C, 71.35; H, 5.41; N, 5.54.

#### 2-{2-[3-(1-Tricyclo[3.3.1.1^3,7^]decyl)-4-fluorophenyl]thiazol-4-yl}ethan-1-amine (**1a**)

A solution of phthalimide **8** (600 mg, 1.23 mmol) and hydrazine hydride (2 mL) in EtOH (20 mL) was refluxed for 1 h and then cooled to 0 °C. The resulting suspension was filtered and the filtrate evaporated. The residue (crude amine **1a**) was used in the next steps without further purification. M.p. (dihydrochloride): 246–248 °C (EtOH/Et_2_O). ^1^H NMR (600 MHz, DMSO-*d*_6_) *δ* 8.35 (bs, 1H, *N*Hth), 7.83–7.73 (m, 2H, 2.6-Har), 7.50 (s, 1H, 5-Hth), 7.18 (dd, *J* = 12.7, 8.4 Hz, 1H, 5-Har), 6.10 (br.s, 4H, NH_4_), 3.31–2.93 (m, 4H, CH_2,_*N*CH_2_), 2.01 (br.s, 3H, 3,5,7-Had), 1.96 (br.s, 6H, 2,8,9-Had), 1.69 (br.s, 6H, 4,6,10-Had). ^13^C NMR (151 MHz, DMSO-*d*_6_) *δ* 166.6 (s, 2-Cth), 162.8 (d, *J* = 251.9 Hz, 4-Car), 152.8 (4-Cth), 137.5 (d, *J* = 11.4 Hz, 3-Car), 129.2 (1-Car), 126.2 (d, *J* = 8.4 Hz, 2-Car), 125.1 (6-Car), 117.5 (d, *J* = 25.4 Hz, 5-Car), 116.4 (5-Cth), 40.5 (2,8,9-Cad), 38.4 (*N*CH_2_), 36.2 (1-Cad), 36.0 (4,6,10-Cad), 28.8 (CH_2_), 28.2 (3,5,7-Cad). Anal. calcd for C_21_H_29_FCl_2_N_2_S: C, 58.74; H, 6.34; N, 6.52 found C, 58.51; H, 6.44; N, 6.68.

#### 2-{2-[3-(1-Tricyclo[3.3.1.1^3,7^]decyl)-4-fluorophenyl]thiazol-4-yl}-*N*-methylethan-1-amine (**1b**)

Ethyl chloroformate (0.1 mL, 1.01 mmol) and Et_3_N (0.15 mL) were added to a stirred solution of the amine **1a** (180 mg, 0.51 mmol) in anhydrous THF (3 mL), at 0 °C, under an argon atmosphere. The reaction mixture was stirred for 5 min at 0 °C and then at ambient temperature, overnight. Next, water was added into the mixture, which was then extracted with EtOAc. The organic phase was washed with water dried over MgSO_4_ and the solvent evaporated. The resulting residue was used in the next step without further purification.

A solution of the crude amide (220 mg, 0.51 mmol) in anhydrous THF (5 mL) was added dropwise to a stirred suspension of LiAlH_4_ (100 mg, 2.52 mmol) in anhydrous THF (5 mL), under an argon atmosphere. The mixture was stirred at ambient temperature for 25 min and then refluxed for 4 h. Next, the reaction mixture was cooled in an ice bath, and ethanol, water and a NaOH (10%) solution were sequentially added. The resulting suspension was then filtered, the filtrate was evaporated *in vacuo* and the resulting residue was treated with water and a HCl (5%) solution. The aqueous phase was then washed with Et_2_O and solid Na_2_CO_3_ was added until pH = 10. The aqueous phase was then extracted with DCM and the combined organic phase was dried over Na_2_CO_3_ and the solvent evaporated *in vacuo* to afford compound **1b**, as a viscous oil (170 mg, 88% from compound **8**). M.p. (dihydrochloride): 221–223 °C (EtOH/Et_2_O). ^1^H NMR (400 MHz, DMSO-*d*_6_) *δ* 9.26 (br.s, 1H, *N*Hth), 7.97 (bs, 2H, *N*H_2_), 7.86–7.76 (m, 2H, 2,6-Har), 7.53 (s, 1H, 5-Hth), 7.24 (dd, *J* = 12.8, 8.3 Hz, 1H, 5-Har), 3.24 (d, *J* = 4.7 Hz, 2H, *N*CH_2_), 3.17 (d, *J* = 7.5 Hz, 2H, CH_2_), 2.57 (t, *J* = 5.4 Hz, 3H, CH_3_), 2.07 (s, 3H, 3,5,7-Had), 2.02 (s, 6H, 2,8,9-Had), 1.74 (s, 6H, 4,6,10-Had). ^13^C NMR (150 MHz, DMSO-*d*_6_) *δ* 166.5 (2-Cth), 162.7 (d, *J* = 251.7 Hz, 4-Car), 152.7 (4-Cth), 137.4 (d, *J* = 11.1 Hz, 3-Car), 129.3 (1-Car), 126.0 (d, *J* = 10.2 Hz, 2-Car), 124.9 (d, *J* = 7.1 Hz, 6-Car), 117.4 (d, *J* = 26.5 Hz, 5-Car), 116.1 (5-Cth), 47.2 (*N*CH_2_), 40.4 (2,8,9-Cad), 36.2 (4,6,10-Cad), 36.0 (1-Cad), 32.4 (CH_3_), 28.1 (3,5,7-Cad), 27.4 (CH_2_). Anal. calcd for C_22_H_31_FCl_2_N_2_S: C, 58.59; H, 6.59; N, 6.32 found C, 58.71; H, 6.25; N, 6.09.

#### 2-{2-[3-(1-Tricyclo[3.3.1.1^3,7^]decyl)-4-fluorophenyl]thiazol-4-yl}-*N*,*N*-dimethylethyl-1-amine (**1c**)

A solution of MeONa (0.1 mL, 30% in MeOH, 0.52 mmol) was added to a stirred solution of compound **1a** dihydrochloride (220 mg, 0.52 mmol) in MeOH (8 mL) and the resulting mixture was stirred for 10 min in ambient temperature. Then acetic acid (0.12 mL, 2 mmol) and NaCNBH_3_ (65 mg, 1.01 mmol) were added into the reaction mixture. Subsequently, a solution of aq. HCHO (38%, 0.1 mL, 1.20 mmol) dissolved in MeOH (2.5 mL) was added dropwise over the course of 30 min and the reaction mixture was stirred at ambient temperature, overnight. The solvent was removed *in vacuo* and an aqueous solution of NaOH (4N, 5 mL) was added. The resulting mixture was then extracted with EtOAc (3 × 20 mL) and the combined organic phases were washed with brine, dried over MgSO_4_ and the solvent evaporated to afford compound **1c**, as a yellow viscous oil (150 mg, 75% from compound **8**). M.p. (dihydrochloride): 280–282 °C (EtOH/Et_2_O). ^1^H NMR (400 MHz, DMSO-*d*_6_) *δ* 10.89 (s, 1H, *N*HTh), 7.86–7.76 (m, 2H, 2,6-Har), 7.53 (s, 1H, 5-Hth), 7.25 (dd, *J* = 12.7, 8.2 Hz, 1H, 5-Har), 6.05 (s, 1H, NH), 3.52–3.36 (m, 2H, *N*CH_2_), 3.30–3.19 (m, 2H, CH_2_), 2.81 (d, *J* = 4.9 Hz, 6H, CH_3_), 2.07 (s, 3H, 3,5,7-Had), 2.02 (s, 6H, 2,8,9-Had), 1.75 (s, 6H, 4,6,10-Had). ^13^C NMR (150 MHz, DMSO-*d*_6_) *δ* 166.9 (2-Cth), 163.1 (d, *J* = 251.8 Hz, 4-Car), 153.0 (4-Cth), 137.9 (d, *J* = 11.1 Hz, 3-Car), 129.8 (d, *J* = 2.8 Hz, 1-Car), 126.4 (d, *J* = 9.8 Hz, 2-Car), 125.3 (d, *J* = 5.4 Hz, 6-Car), 117.8 (d, *J* = 25.6 Hz, 5-Car), 116.6 (5-Cth), 55.8 (*N*CH_2_), 42.5 (CH_3_), 40.9 (2,8,9-Cad), 36.6 (4,6,10-Cad), 36.4 (1-Cad), 28.5 (3,5,7-Cad), 26.4 (CH_2_). Anal. calcd for C_23_H_33_FCl_2_N_2_S: C, 60.39; H, 6.83; N, 6.12 found C, 60.50; H, 6.91; N, 6.41.

#### General method for the preparation of amides **1d–g**, **i**, **j** and **2d**

A stirred solution of amine **1a** or **2a** (1 eq.) and Et_3_N (0.5 mL) in EtOAc (7 mL) was cooled to 0 °C and the appropriate acid chloride or anhydride (2–3 eq.) was added under an argon atmosphere. The reaction mixture was stirred at ambient temperature for 24–48 h. Water was added into the mixture, which was then extracted with EtOAc. The combined organic layers were washed with water, dried over Na_2_SO_4_, the solvent evaporated and the residue was purified by column chromatography.

#### *N*-{2-[2-(3-(1-Tricyclo[3.3.1.1^3,7^]decyl)-4-fluorophenyl]thiazol-4-yl]ethyl}acetamide (**1d**)

Acetamide **1d** was prepared, as described in the general method, using acetic anhydride (3 eq.). Elution with EtOAc afforded compound **1d** as a foamy solid (140 mg, 84% from compound **8**). ^1^H NMR (400 MHz, CDCl_3_) *δ* 7.85 (dd, *J* = 7.7, 2.2 Hz, 1H, 2-Har), 7.70 (ddd, *J* = 8.2, 4.3, 2.2 Hz, 1H, 6-Har), 7.04 (dd, *J* = 12.4, 8.4 Hz, 1H, 5-Har), 6.95 (s, 1H, 5-Hth), 6.49 (br.s, 1H, NH), 3.64 (q, *J* = 6.3 Hz, 2H, CH_2_N), 2.98 (t, *J* = 6.3 Hz, 2H, CH_2_), 2.09 (br.s, *J* = 14.7 Hz, 9H, 3,5,7,2,8,9-Had), 1.99 (s, 3H, CH_3_), 1.80 (br.s, 6H, 4,6,10-Had). ^13^C NMR (50 MHz, CDCl_3_) *δ* 170.1 (C=O), 166.1 (2-Cth), 164.5 (d, *J* = 349.7 Hz, 4-Car), 155.5 (4-Cth), 138.6 (d, *J* = 11.2 Hz, 3-Car), 129.6 (1-Car), 125.8 (d, *J* = 2.9 Hz, 6-Car), 125.6 (2-Car), 117.2 (d, *J* = 25.8 Hz, 5-Car), 114.2 (5-Cth), 41.1 (d, *J* = 3.5 Hz, 2,8,9-Cad), 39.1 (CH_2_*N*), 36.9 (4,6,10-Cad), 36.7 (1-Cad), 31.0 (CH_2_), 28.9 (3,5,7-Cad), 23.5 (CH_3_). M.p. (fumarate): 143-145 °C (MeOH/Et_2_O). Anal. calcd for C_27_H_31_FN_2_O_5_S: C, 63.03; H, 6.07; N, 5.44 found C, 62.89; H, 6.01; N, 5.21.

#### *N*-{2-[2-(3-(1-Tricyclo[3.3.1.1^3,7^]decyl)-4-fluorophenyl)thiazol-4-yl]ethyl}butylamide (**1e**)

Butylamide **1e** was prepared, as described in general method, using butyric anhydride (3 eq.). Elution with 50% EtOAc in hexanes afforded compound **1e** as a foamy solid (165 mg, 92% from **8**). ^1^H NMR (400 MHz, CDCl_3_) *δ* 7.85 (dd, *J* = 7.7, 2.2 Hz, 1H, 2-Har), 7.70 (ddd, *J* = 8.2, 4.3, 2.2 Hz, 1H, 6-Har), 7.04 (dd, *J* = 12.4, 8.4 Hz, 1H, 5-Har), 6.95 (s, 1H, 5-Hth), 6.49 (br.s, 1H, *N*H), 3.64 (q, *J* = 6.3 Hz, 2H, CH_2_*N*), 2.98 (t, *J* = 6.3 Hz, 2H, CH_2_), 2.09 (br.s, *J* = 14.7 Hz, 9H, 3,5,7,2,8,9-Had), 2.18 (t, *J* = 6.9 Hz, 2H, CH_2_CO), 1.80 (br.s, 6H, 4,6,10-Had), 1.67 (h, *J* = 6.9 Hz, 2H, CH_2_), 0.94 (t, *J* = 7.4 Hz, 3H, CH_3_). ^13^C NMR (50 MHz, CDCl_3_) *δ* 170.2 (C=O), 166.1 (2-Cth), 164.5 (d, *J* = 349.7 Hz, 4-Car), 155.5 (4-Cth), 138.3 (d, *J* = 11.2 Hz, 3-Car), 129.6 (1-Car), 125.8 (d, *J* = 2.9 Hz, 6-Car), 125.6 (2-Car), 117.2 (d, *J* = 25.8 Hz, 5-Car), 114.3 (5-Cth), 41.1 (2,8,9-Cad), 39.2 (CH_2_*N*), 36.9 (4,6,10-Cad), 36.7 (1-Cad), 31.0 (CH_2_), 28.9 (3,5,7-Cad), 28.9 (CH_2_), 19.3 (CH_2_), 13.9 (CH_3_). M.p. (fumarate): 291–293 °C (MeOH/Et_2_O). Anal. calcd for C_29_H_35_FN_2_O_5_S: C, 64.19; H, 6.50; N, 5.16 found C, 64.38; H, 6.61; N, 5.33.

#### *N*-{2-[2-(3-(1-Tricyclo[3.3.1.1^3,7^]decyl)-4-fluorophenyl)thiazol-4-yl] ethyl}benzamide (**1f**)

Benzamide **1f** was prepared, as described in general method, using benzoyl chloride (2 eq.). Elution with 30% EtOAc in hexanes afforded compound **1f** as a foamy solid (186 mg, 96%, from **8**). ^1^H NMR (400 MHz, CDCl_3_) *δ* 7.91–7.82 (m, 2H, 2′,6′-Har), 7.85 (dd, *J* = 7.7, 2.2 Hz, 1H, 2-Har), 7.70 (ddd, *J* = 8.2, 4.3, 2.2 Hz, 1H, 6-Har), 7.59–7.51 (m, 1H, 4′-Har), 7.46–7.36 (m, 2H, 3′,5′-Har), 7.04 (dd, *J* = 12.4, 8.4 Hz, 1H, 5-Har), 6.95 (s, 1H, 5-Hth), 6.49 (br.s, 1H, *N*H), 3.64 (q, *J* = 6.3 Hz, 2H, CH_2_*N*), 2.98 (t, *J* = 6.3 Hz, 2H, CH_2_), 2.09 (br.s, *J* = 14.7 Hz, 9H, 3,5,7-Had, 2,8,9-Had), 1.80 (bs, 6H, 4,6,10-Had). ^13^C NMR (50 MHz, CDCl_3_) *δ* 166.1 (2-Cth), 166.0 (C=O), 164.5 (d, *J* = 349.7 Hz, 4-Car), 155.5 (4-Cth), 138.3 (d, *J* = 11.2 Hz, 3-Car), 131.3 (4′-Car), 129.6 (1-Car), 128.5 (3′,5′-Car), 127.0 (2′,6′-Car), 125.8 (d, *J* = 2.9 Hz, 6-Car), 125.6 (2-Car), 117.2 (d, *J* = 25.8 Hz, 5-Car), 114.3 (5-Cth), 41.1 (2,8,9-Cad), 39.2 (CH_2_*N*), 36.9 (4,6,10-Cad), 36.7 (1-Cad), 31.0 (CH_2_), 28.9 (3,5,7-Cad). M.p. (fumarate): 305 °C (dec) (MeOH/Et_2_O). Anal. calcd for C_32_H_33_FN_2_O_5_S: C, 66.65; H, 5.77; N, 4.86 found C, 65.39; H, 5.90; N, 4.72.

#### *N*-{2-[2-(3-(1-Tricyclo[3.3.1.1^3,7^]decyl)-4-fluorophenyl)thiazol-4-yl]ethyl}-4-fluorobenzamide (**1g**)

4-Fluorobenzamide **1g** was prepared, as described in general method, using 4-fluorobenzoyl chloride (3 eq.). Elution with 50% EtOAc in hexanes afforded compound **1g** as a foamy solid (185 mg, 93% from compound **8**). ^1^H NMR (400 MHz, DMSO-*d*_6_) *δ* 8.05 (br.s, 1H, *N*H), 7.80 (m, 2H, 2′,6′-Har), 7.75 (dd, *J* = 7.7, 2.0 Hz, 1H, 2-Har), 7.68 (dd, *J* = 7.7, 8.9 Hz, 1H, 6-Har), 7.12–6.96 (m, 4H, 5-Har, 5-Hth, 3′,5′-Har), 3.70 (q, *J* = 5.8 Hz, 2H, CH_2_*N*), 3.05 (t, *J* = 6.7 Hz, 2H, CH_2_), 2.03 (s, 3H, 3,5,7-Had), 1.99 (s, 6H, 2,8,9-Had), 1.72 (s, 6H, 4,6,10-Had). ^13^C NMR (50 MHz, CDCl_3_) *δ* 166.1 (2-Cth), 166.0 (C=O), 164.5 (d, *J* = 349.7 Hz, 4-Car), 163.9 (d, *J* = 348.6 Hz, 4′-Car) 155.5 (4-Cth), 138.3 (d, *J* = 11.2 Hz, 3-Car), 130.1 (d, *J* = 3 Hz, 1′-Car), 129.6 (1-Car), 128.3 (d, *J* = 7 Hz, 2′,6′-Car), 125.8 (d, *J* = 2.9 Hz, 6-Car), 125.6 (2-Car), 117.2 (d, *J* = 25.8 Hz, 5-Car), 116.1 (d, *J* = 18 Hz, 3′,5′-Car) 114.3 (5-Cth), 41.1 (d, *J* = 3.5 Hz, 2,8,9-Cad), 39.2 (CH_2_N), 36.9 (4,6,10-Cad), 36.7 (1-Cad), 31.0 (CH_2_), 28.9 (3,5,7-Cad). M.p. (fumarate): 226–227 °C (MeOH/Et_2_O). Anal. calcd for C_32_H_32_F_2_N_2_O_5_S: C, 64.63; H, 5.42; N, 4.71 found C, 64.44; H, 5.73; N, 4.88.

#### *N*-{2-[2-(3-(1-Tricyclo[3.3.1.1^3,7^]decyl)-4-fluorophenyl)thiazol-4-yl] ethyl}-3-methylfuran-2-ylcarboxamide (**1h**)

DMAP (57 mg, 0.46 mmol), EDCI (90 mg, 0.46 mmol) and HOBt (65 mg 0.41 mmol) were added to a stirred mixture of the amine **1a** (150 mg, 0.41 mmol) and 3-methylfuroic acid (52 mg, 0.41 mmol) in anhydrous DMF (2 mL) and anhydrous DCM (1 mL) at 0 °C, under an argon atmosphere. The reaction mixture was stirred at the same temperature for 1 h and then at 45 °C, overnight. Water was added into the mixture, which was then extracted with EtOAc. The organic phase was washed with water dried over Na_2_SO_4_ and the solvent evaporated *in vacuo*. The residue was purified by column chromatography. Elution with EtOAc afforded compound **1h** as a foamy solid (50 mg, 29% from compound **8**).^1^H NMR (400 MHz, CDCl_3_) *δ* 7.85 (dd, *J* = 7.7, 2.2 Hz, 1H, 2-Har), 7.70 (ddd, *J* = 8.2, 4.3, 2.2 Hz, 1H, 6-Har), 7.30 (d, *J* = 0.9 Hz, 1H, 5-Hfur) 7.04 (dd, *J* = 12.4, 8.4 Hz, 1H, 5-Har), 6.95 (s, 1H, 5-Hth), 6.49 (bs, 1H, NH), 6.35 (d, *J* = 1.0 Hz, 1H, 4-Hfur), 3.64 (q, *J* = 6.3 Hz, 2H, CH_2_*N*), 2.98 (t, *J* = 6.3 Hz, 2H, CH_2_), 2.40 (s, 3H, CH_3_), 2.09 (br.s, *J* = 14.7 Hz, 9H, 3,5,7,2,8,9-Had), 1.80 (br.s, 6H, 4,6,10-Had). ^13^C NMR (50 MHz, CDCl_3_) *δ* 166.1 (2-Cth), 164.5 (d, *J* = 349.7 Hz, 4-Car), 160.6 (C=O), 155.5 (4-Cth), 142.7 (4-Cfur), 141.1 (1-Cfur), 138.3 (d, *J* = 11.2 Hz, 3-Car), 129.6 (1-Car), 125.8 (d, *J* = 2.9 Hz, 6-Car), 125.6 (2-Car), 117.2 (d, *J* = 25.8 Hz, 5-Car), 115.6 (2-Cfur), 115.2 (5-Cth), 114.3 (3-Cfur), 41.1 (2,8,9-Cad), 39.2 (CH_2_N), 36.9 (4,6,10-Cad), 36.7 (1-Cad), 31.0 (CH_2_), 28.9 (3,5,7-Cad), 11.0 (CH_3_). M.p. (fumarate): 260 (dec) °C (MeOH/Et_2_O). Anal. Calcd for C_31_H_32_FN_2_O_6_S: C, 64.12; H, 5.73; N, 4.82 found C, 64.44; H, 5.54; N, 4.98.

#### *N*-(2-(2-(3-(1-Tricyclo[3.3.1.1^3,7^]decyl)-4-fluorophenyl)thiazol-4-yl) ethyl)pyrrolidin-1-yl-carboxamide (**1i**)

Carboxamide **1i** was prepared, as described in general method, using 1-pyrrolidinecarbonyl chloride (2 eq.). Elution with 75% EtOAc in hexanes afforded compound **1i** as a foamy solid (80 mg, 46% from compound **8**). ^1^H NMR (400 MHz, CDCl_3_) *δ* 7.85 (dd, *J* = 7.7, 2.2 Hz, 1H, 2-Har), 7.70 (ddd, *J* = 8.2, 4.3, 2.2 Hz, 1H, 6-Har), 7.04 (dd, *J* = 12.4, 8.4 Hz, 1H, 5-Har), 6.95 (s, 1H, 5-Hth), 5.24 (br.s, 1H, *N*H), 3.64 (q, *J* = 6.3 Hz, 2H, CH_2_*N*), 3.46–3.26 (m, 4H, 2,5-Hpy) 2.98 (t, *J* = 6.3 Hz, 2H, CH_2_), 2.09 (br.s, 9H, 3,5,7,2,8,9-Had), 1.94–1.85 (m, 4H, 3,4-Hpy), 1.80 (br.s, 6H, 4,6,10-Had). ^13^C NMR (50 MHz, CDCl_3_) *δ* 166.1 (2-Cth), 164.5 (d, *J* = 349.7 Hz, 4-Car), 156.9 (C=O), 155.5 (4-Cth), 138.3 (d, *J* = 11.2 Hz, 3-Car), 129.6 (1-Car), 125.8 (d, *J* = 2.9 Hz, 6-Car), 125.6 (2-Car), 117.2 (d, *J* = 25.8 Hz, 5-Car), 114.3 (5-Cth), 45.4 (2,5-Cpy), 41.1 (d, *J* = 3.5 Hz, 2,8,9-Cad), 39.2 (CH_2_*N*), 36.9 (4,6,10-Cad), 36.7 (1-Cad), 31.0 (CH_2_), 28.9 (3,5,7-Cad), 25.6 (3,4-Cpy). M.p. (fumarate): 301 °C (dec) (MeOH/Et_2_O). Anal. calcd for C_30_H_36_FN_3_O_5_S: C, 63.25; H, 6.37; N, 7.38 found C, 63.09; H, 6.14; N, 7.19.

#### *N*-{2-[2-(3-(1-Tricyclo[3.3.1.1^3,7^]decyl)-4-fluorophenyl)thiazol-4-yl}ethyl)piperidine-1-yl-carboxamide (**1j**)

Carboxamide **1j** was prepared, as described in general method, using 1-piperidinecarbonyl chloride (2 eq.). Elution with 50% EtOAc in hexanes afforded compound **1j** as a foamy solid (50 mg, 36% from compound **8**). ^1^H NMR (400 MHz, CDCl_3_) *δ* 7.85 (dd, *J* = 7.7, 2.2 Hz, 1H, 2-Har), 7.70 (ddd, *J* = 8.2, 4.3, 2.2 Hz, 1H, 6-Har), 7.04 (dd, *J* = 12.4, 8.4 Hz, 1H, 5-Har), 6.95 (s, 1H, 5-Hth), 5.24 (br.s, 1H, *N*H), 3.64 (q, *J* = 6.3 Hz, 2H, CH_2_N), 3.51–3.20 (m, 4H, 2,6-Hpi) 2.98 (t, *J* = 6.3 Hz, 2H, CH_2_), 2.09 (br.s, *J* = 14.7 Hz, 9H, 3,5,7,2,8,9-Had), 1.80 (bs, 6H, 4,6,10-Had), 1.66–1.39 (m, 6H, 3,4,5-Hpi). ^13^C NMR (150 MHz, CDCl_3_) *δ* 166.6 (2-Cth), 162.8 (d, *J* = 251.9 Hz, 4-Car), 156.9 (C=O), 152.8 (4-Cth), 137.5 (d, *J* = 11.4 Hz, 3-Car), 129.2 (1-Car), 126.2 (d, *J* = 8.4 Hz, 2-Car), 125.1 (6-Car), 117.5 (d, *J* = 25.4, 5-Car), 116.4 (5-Cth), 43.9 (2,6-Cpi), 40.5 (2,8,9-Cad), 38.4 (*N*CH_2_), 36.2 (1-Cad), 36.0 (4,6,10-Cad), 28.8 (CH_2_), 28.2 (3,5,7-Cad), 24.8 (3,5-Cpi), 23.5 (4-Cpi). Anal. calcd for C_27_H_34_FN_3_OS: C, 69.35; H, 7.33; N, 8.99 found C, 69.55; H, 7.39; N, 9.12.

#### 4-(1-Tricyclo[3.3.1.1^3,7^]decyl)benzoic acid (**12**)

Oxygen gas was bubbled into a stirred solution of 1-(4-tolyl)adamantane (**11**)^33^ (1.4 g, 6.17 mmol), Co(OAc)_2_ (73 mg, 0.31 mmol), Mn(OAc)_2_ (9 mg, 0.03 mmol) and NaBr (35 mg, 0.32 mmol) in AcOH (26 mL)/dioxane (2.8 mL)/H_2_O (0.7 mL) for 6 h, at 105 °C. The resulting mixture was cooled to ambient temperature and water was added into the mixture. The residue obtained was filtered and washed with water. The resulting solid was then dried *in vacuo*, in the presence of P_2_O_5_, overnight to afford compound **12**, as a white solid (1.4 g, 89%). ^1^H NMR (400 MHz, CDCl_3_) *δ* 12.30 (br.s, 1H, OH) 8.04 (d, *J* = 8.4 Hz, 2H, 3,5-Har), 7.46 (d, *J* = 8.5 Hz, 2H, 2,6-Har), 2.12 (s, 3H, 3,5,7-Had), 1.94 (s, 6H, 2,8,9-Had), 1.78 (d, *J* = 7.1 Hz, 6H, 4,6,10-Had).

#### 4-(1-Tricyclo[3.3.1.1^3,7^]decyl)benzamide (**13**)

A solution of 4-(adamant-1-yl)benzoic acid (**12**) (2 g, 6.17 mmol) in SOCl_2_ (12 ml) was heated at 65 °C for 45 min. The excess of SOCl_2_ was removed under reduced pressure and subsequently by azeotropic distillation with benzene. The resulting precipitate was then dissolved in anhydrous THF (10 mL) and added to a stirred solution of NH_3_ (25%) in water (50 mL), dropwise, at 0 °C. The reaction mixture was stirred for 30 min at ambient temperature and extracted with EtOAc. The organic layer dried over MgSO_4_ and the solvent evaporated to afford compound **13** as an off white solid (1.9 g, 96%). ^1^H NMR (400 MHz, CDCl_3_) *δ* 7.83 (d, *J* = 8.5 Hz, 2H, 3,5-Har), 7.64 (br.s, 2H, *N*H_2_), 7.39 (d, *J* = 8.5 Hz, 2H, 2,6-Har), 2.11 (s, 3H, 3,5,7-Had), 1.90 (br.s, 6H, 2,8,9-Had), 1.77 (q, *J* = 12.2 Hz, 6H, 4,6,10-Had). Anal. calcd for C_17_H_21_NO: C, 79.96; H, 8.29 found C, 79.77; H, 8.57.

#### 4-(1-Tricyclo[3.3.1.1^3,7^]decyl)thiobenzamide (**14**)

Lawesson's reagent (1.2 g) was added to a stirred solution of benzamide **13** (1.5 g, 6.17 mmol) in dioxane (15 mL) and the reaction mixture was heated to 110 °C, overnight. The solvent was removed *in vacuo* and the crude residue was crystallised from DCM. The filtrate of the recrystallisation still contained benzamide **13**, thus it was purified by column chromatography. Elution with DCM afforded compound **14** as a yellow solid (780 mg, 50%). M.p.: 201–202 °C ^1^H NMR (400 MHz, CDCl_3_) *δ* 7.83 (d, *J* = 8.5 Hz, 2H, 3,5-Har), 7.64 (br.s, 1H, *N*H), 7.39 (d, *J* = 8.5 Hz, 2H, 2,6-Har), 7.20 (s, 1H, *N*H), 2.11 (s, 3H, 3,5,7-Had), 1.90 (br.s, 6H, 2,8,9-Had), 1.77 (q, *J* = 12.2 Hz, 6H, 4,6,10-Had). ^13^C NMR (150 MHz, CDCl_3_) *δ* 202.6 (C=S), 156.0 (1-Car), 136.4 (4-Car), 126.9 (2,6-Car), 125.1 (3,5-Car), 42.9 (2,8,9-Cad), 36.7 (4,6,10-Cad), 36.6 (1-Cad), 28.8 (3,5,7-Cad). C_17_H_21_NS: C, 75.23; H, 7.80 found C, 75.07; H, 7.99.

#### 2-{2-[2-(1-Tricyclo[3.3.1.1^3,7^]decyl)phenyl]ethyl}isoindoline-1,3-dione (**10**)

##### Method A

Phthalimide **10** was prepared in a similar way as for compound **8** using pinacolborane **9** (ref. 31) (300 mg, 0.88 mmol) and bromothiazole **7** (ref. 6) (250 mg, 0.73 mmol) as starting materials. Elution with 10–20% EtOAc in hexanes afforded compound **10** as a white solid (300 mg, 87%).

##### Method B

The bromoketone **15** (ref. 6) (200 mg, 0.66 mmol) was added to a stirred solution of the thiobenzamide **14** (180 mg, 0.66 mmol) in EtOH (4 mL) and the reaction mixture was refluxed overnight. The resulting suspension was filtered and the precipitate was washed with Et_2_O and dried over MgSO_4_ to afford compound **10**, as a white solid (200 mg, 65%) M.p.: 213–214 °C. ^1^H NMR (600 MHz, CDCl_3_) *δ* 7.81 (dt, *J* = 6.8, 3.4 Hz, 2H, 2′,5′-Har), 7.73 (d, *J* = 8.4 Hz, 2H, 2,6-Har), 7.66 (dt, *J* = 6.8, 3.4 Hz, 2H, 3′,4′-Har), 7.34 (d, *J* = 8.4 Hz, 2H, 3,5-Har), 6.94 (s, 1H, 5-Hth), 4.10 (t, *J* = 7.1 Hz, 2H, *N*CH_2_), 3.21 (t, *J* = 7.1 Hz, 2H, CH_2_), 2.12 (br.s, 3H, 3,5,7-Had), 1.91 (br.s, 6H, 2,8,9-Had), 1.78 (dd, *J* = 25.4, 12.1 Hz, 6H, 4,6,10-Had). ^13^C NMR (150 MHz, CDCl_3_) *δ* 168.4 (C=O), 168.2 (2-Cth), 154.2 (4-Cth), 153.4 (1-Car), 133.9 (3′,4′-Car), 132.3 (1′,6′-Car), 131.1 (4-Car), 126.4 (2,6-Car), 125.4 (3,5-Car), 123.3 (2′,5′-Car), 114.2 (5-Cth), 43.1 (2,8,9-Cad), 37.7 (NCH_2_), 36.9 (4,6,10-Cad), 36.5 (1-Cad), 30.2 (CH_2_), 29.0 (3,5,7-Cad). C_29_H_28_N_2_O_2_S: C, 74.33; H, 6.02; N, 5.98 found C, 74.64; H, 6.12; N, 6.21.

#### 2-{2-[4-(1-Tricyclo[3.3.1.1^3,7^]decyl)phenyl]thiazol-4-yl}ethan-1-amine (**2a**)

The amine **2a** was prepared in a similar way as the amine **1a**, using phthalimide **10** as starting material to afford **2a** as a green solid. M.p. (dihydrochloride): 225 °C (dec) (MeOH/Et_2_O). ^1^H NMR (400 MHz, DMSO-*d*_6_) *δ* 8.31 (s, 1H, *N*Hth), 7.86 (d, *J* = 8.3 Hz, 2H, 2,6-Har), 7.52 – 7.44 (m, 3H, 3,5-Har, 5-Hth), 6.75 (s, 3H, *N*H_3_), 3.20–3.07 (m, 4H, CH_2_, NCH_2_), 2.06 (s, 3H, 3,5,7-Had), 1.88 (s, 6H, 2,8,9-Had), 1.74 (s, 6H, 4,6,10-Had).^13^C NMR (75 MHz, DMSO-*d*_6_) *δ* 167.5 (2-Cth), 153.6 (4-Cth), 153.4 (1-Car), 130.9 (4-Car) 126.4 (2,6-Car), 126.0 (3,5-Car), 116.1 (5-Cth), 42.8 (2,8,9-Cad), 38.6 (NCH_2_), 36.5 (4,6,10-Cad), 36.4 (1-Cad), 29.2 (CH_2_), 28.7 (3,5,7-Cad). Anal. calcd for C_21_H_27_Cl_2_N_2_S: C, 61.31; H, 6.86; N, 6.81 found C, 61.19; H, 6.95; N, 6.69.

#### 2-{2-[4-(1-Tricyclo[3.3.1.1^3,7^]decyl)phenyl]thiazol-4-yl}-*N*-methylethan-1-amine (**2b**)

The amine **2b** was prepared in a similar way as the methylamine **1b**, using the amine **2a** (180 mg, 0.51 mmol) as starting material to afford compound **2b** as yellow solid (95 mg, 54% from **10**). M.p. (difumarate): 147–149 °C (EtOH/Et_2_O). ^1^H NMR (600 MHz, DMSO-*d*_6_) *δ* 7.89–7.83 (d, *J* = 8.2 Hz, 2H, 2,6-Har), 7.46 (m, 3H, 5-Hth, 3,5-Har), 6.57 (s, 4H, Hfum), 3.31–3.22 (m, 2H, *N*CH_2_), 3.17–3.08 (m, 2H, CH_2_), 2.68 (br.s, 2H, *N*H_2_), 2.60 (s, 3H, CH_3_), 2.06 (br.s, 3H, 3,5,7-Had), 1.87 (br.s, 6H, 2,8,9-Had), 1.75 (br.s, 6H, 4,6,10-Had). ^13^C NMR (150 MHz, DMSO-*d*_6_) *δ* 167.2 (2-Cth), 167.0 (C=O, fum), 153.2 (1-Car), 152.9 (4-Cth), 134.6 (C-fum), 130.5 (4-Car), 126.0 (2,6-Car), 125.6 (3,5-Car), 115.7 (5-Cth), 47.4 (*N*CH_2_), 42.4 (2,8,9-Cad), 36.1 (4,6,10-Cad), 36.0 (1-Cad), 32.5 (CH_3_), 28.3 (3,5,7-Cad), 27.6 (CH_2_). Anal. calcd for C_30_H_36_N_2_O_8_S: C, 61.63; H, 6.21; N, 4.79 found C, 61.47; H, 6.08; N, 5.01.

#### 2-{2-[4-(1-Tricyclo[3.3.1.1^3,7^]decyl)phenyl]thiazol-4-yl}-*N*,*N*-dimethylethan-1-amine (**2c**)

Dimethylamine **2c** was prepared in a similar way as the dimethylamine **1c**, using the amine **2a** (220 mg, 0.64 mmol), as starting material to afford compound **2c** as yellow solid (150 mg, 85% from **10**). M.p. (difumarate): 302 °C (dec) (EtOH/Et_2_O). ^1^H NMR (400 MHz, DMSO-*d*_6_) *δ* 7.89–7.83 (d, *J* = 8.2 Hz, 2H, 2,6-Har), 7.46 (m, 3H, 5-Hth, 3,5-Har), 6.57 (s, 4H, Hfum), 3.31–3.22 (m, 2H, *N*CH_2_), 3.17–3.08 (m, 2H, CH_2_), 2.68 (br.s, 2H, *N*H), 2.46 (s, 6H, CH_3_), 2.06 (br.s, 3H, 3,5,7-Had), 1.87 (br.s, 6H, 2,8,9-Had), 1.75 (br.s, 6H, 4,6,10-Had). ^13^C NMR (150 MHz, DMSO-*d*_6_) *δ* 167.5 (4-Cth), 166.6 (C=O, fum), 153.7 (4-Cth), 134.7 (1-Car), 134.6 (CH, fum), 130.9 (4-Car), 126.4 (2,6-Car), 126.0 (3,5-Car), 116.0 (5-Cth), 56.4 (CH_2_N), 43.0 (CH_3_), 42.9 (2,8,9-Cad), 36.6 (4,6,10-Cad), 36.5 (1-Cad), 28.7 (3,5,7-Cad), 27.0 (CH_2_). Anal. calcd for C_31_H_38_N_2_O_8_S: C, 62.19; H, 6.40; N, 4.68 found C, 62.31; H, 6.64; N, 4.14.

#### *N*-{2-[2-(4-(1-Tricyclo[3.3.1.1^3,7^]decyl)phenyl)thiazol-4-yl]ethyl}acetamide (**2d**)

The acetamide **2d** was prepared as described in the general method, using the amine **2a** (150 mg, 0.42 mmol) and acetic anhydride (0.15 ml, 1.42 mmol). Elution with 10% MeOH in DCM afforded compound **2d** as a white solid (100 mg, 53% from compound **10**). M.p. (fumarate): 164–166 °C (MeOH/Et_2_O). ^1^H NMR (600 MHz, DMSO-*d*_6_) *δ* 13.12 (s, 1H, *N*HTh), 7.95 (s, 1H *N*HC=O), 7.84 (d, *J* = 8.2 Hz, 2H, 2,6-Har), 7.46 (d, *J* = 8.2 Hz, 2H, 3,5-Har), 7.34 (s, 1H, 5-Hth), 6.63 (s, 2H, CH-Fum), 3.39 (dt, *J* = 13.3, 6.7 Hz, 2H, *N*CH_2_), 2.88 (t, *J* = 7.2 Hz, 2H, CH_2_), 2.06 (s, 3H, 3,5,7-Had), 1.88 (s, 6H, 2,8,9-Had), 1.80 (s, 3H, CH_3_), 1.74 (br.s, 6H, 4,6,10-Had). ^13^C NMR (75 MHz, DMSO-*d*_6_) *δ* 169.1 (*N*C=O), 166.5 (2-Cth), 166.0 (C=O, fum), 155.2 (4-Cth), 153.0 (1-Car), 134.0 (CH-fum), 130.7 (4-Car), 125.9 (2,6-Car), 125.5 (3,5-Car), 114.6 (5-Cth), 42.4 (2,8,9-Cad), 38.2 (*N*CH_2_), 36.1 (4,6,10-Cad), 36.0 (1-Cad), 31.3 (CH_2_), 28.3 (3,5,7-Cad), 22.7 (CH_3_). Anal. calcd for C_27_H_32_N_2_O_5_S: C, 65.30; H, 6.50; N, 5.64 found C, 65.27; H, 6.33; N, 5.45.

#### 2-[4-(1-Tricyclo[3.3.1.1^3,7^]decyl)phenyl]-4-(chloromethyl)thiazole (**15**)

Thiobenzamide **14** (200 mg, 0.74 mmol) was added to a stirred solution of 1-3-dichloroacetone (125 mg, 0.99 mmol) in acetone (4 mL) and the reaction mixture was refluxed overnight. The solvent was removed *in vacuo* and the resulting residue was dissolved in conc. H_2_SO_4_ (5 mL), stirred for 30 min and subsequently poured onto a mixture of water and ice. The resulting suspension was the filtered and the precipitate was washed with water to afford compound **15** as a yellow-white solid (190 mg, 75%), which was used in the next step without further purification.

#### 2-{2-[4-(1-Tricyclo[3.3.1.1^3,7^]decyl)phenyl]thiazol-4-yl}acetonitrile (**2e**)

A solution of the chloride **15** (180 mg, 0.52 mmol) and KCN (260 mg, 5.24 mmol) in anhydrous DMF (2 mL), was heated at 60 °C under an argon atmosphere for 36 h and then chilled to room temperature. Water was then added into the reaction mixture, which was extracted with EtOAc. The organic layer was washed with water and brine, dried over MgSO_4_, and then the solvent was evaporated *in vacuo*. The solid residue was then purified by column chromatography. Elution with 20% EtOAc in hexanes afforded compound **2e** as a yellow crystalline solid (70 mg, 41%). M.p.: 159–160 °C. ^1^H NMR (400 MHz, CDCl_3_) *δ* 7.86 (d, *J* = 8.5 Hz, 2H, 2,6H-ar), 7.44 (d, *J* = 8.4 Hz, 2H, 3,5-Har), 7.26 (s, 1H, 5-Hth), 3.95 (s, 2H, CH_2_), 2.12 (s, 3H, 3,5,7-Had), 1.93 (d, *J* = 2.1 Hz, 6H, 2,8,9-Had), 1.79 (q, 6H, 4,6,10-Had). ^13^C NMR (150 MHz, CDCl_3_) *δ* 169.7 (2-Cth), 154.2 (1-Car), 145.6 (4-Cth), 130.4 (4-Car), 126.4 (2,6-Car), 125.6 (3,5-Car), 116.9 (CN), 115.6 (5-Cth), 43.0 (2,8,9-Cad), 36.7 (4,6,10-Cad), 36.5 (1-Cad), 28.9 (3,5,7-Cad), 20.7 (CH_2_). Anal. calcd for C_21_H_22_N_2_S: C, 75.41; H, 6.63; N, 8.38 found C, 74.34; H, 6.29; N, 8.51.

#### 2-[4-(1-Tricyclo[3.3.1.1^3,7^]decyl)phenyl]-4-(thiocyanatemethyl)thiazole (**2f**)

A solution of the chloride **15** (250 mg, 0.73 mmol) and KSCN (100 mg, 1.34 mmol) in EtOH (4 ml), was heated to 45 °C under an argon atmosphere overnight. The reaction mixture was then poured onto a mixture of ice and water and the resulting suspension was filtered. The residue obtained was crystallized from EtOH to afford compound **2f** as a white solid (250 mg, 67%). M.p.: 169–170 °C. ^1^H NMR (600 MHz, CDCl_3_) *δ* 7.85 (d, *J* = 8.4 Hz, 2H, 2,6H-ar), 7.41 (d, *J* = 8.4 Hz, 2H, 3,5-Har), 7.23 (s, 1H, 5-Hth), 4.29 (s, 2H, CH_2_), 2.09 (s, 3H, 3,5,7-Had), 1.91 (d, 6H, 2,8,9-Had), 1.76 (q, 6H, 4,6,10-Had). ^13^C NMR (150 MHz, CDCl_3_) *δ* 169.7 (2-Cth), 154.2 (1-Car), 149.7 (4-Cth), 130.4 (4-Car), 126.5 (2,6-Car), 125.6 (3,5-Car), 117.5 (5-Cth), 112.0 (SCN), 43.0 (2,8,9-Cad), 36.7 (4,6,10-Cad), 36.50 (1-Cad), 33.9 (CH_2_), 28.9 (3,5,7-Cad). Anal. calcd for C_21_H_22_N_2_S_2_: C, 68.81; H, 6.05; N, 7.64 found C, 68.81; H, 6.09; N, 7.59.

#### Ethyl 2-{2-[4-(1-Tricyclo[3.3.1.1^3,7^]decyl)phenyl]thiazol-4-yl}acetate (**16**)

4-Chloroacetoacetate (700 mg, 2.43 mmol) was added to a stirred mixture of thiobenzamide **14** (1 g, 3.68 mmol) in i-PrOH (8 mL), and the reaction mixture was stirred overnight, at 110 °C, in an autoclave. The solvent was then removed *in vacuo* and the resulting residue was dissolved in EtOAc and washed with water, a saturated aqueous solution of NaHCO_3_ and brine. The organic layer was then dried over MgSO_4_ and the solvent evaporated under vacuum. The resulting oil was triturated with EtOAc/hexanes to afford compound **16** as a light orange solid (1.3 g, 92%) which was used in the next step without further purification.

#### 2-{2-[4-(1-Tricyclo[3.3.1.1^3,7^]decyl)phenyl]thiazol-4-yl}ethan-1-ol (**2g**)

To a stirred suspension of LiAlH_4_ (85 mg, 2.23 mmol) in anhydrous THF (5 mL), was added dropwise a solution of compound **16** (170 mg, 0.44 mmol) in anhydrous THF (3 mL), under an argon atmosphere and then the reaction mixture was stirred at ambient temperature for 2 h. Next, the mixture was cooled in an ice bath and ethanol, water and a NaOH (10%) solution were added in order. The resulting suspension was then filtered, the filtrate was evaporated *in vacuo* and extracted with DCM. The organic layer was then washed with water, dried over MgSO_4_ and the solvent evaporated to afford compound **2g** as an off white solid (120 mg, 80%). M.p.: 93–94 °C. ^1^H NMR (400 MHz, CDCl_3_) *δ* 7.86 (d, *J* = 8.4 Hz, 2H, 2,6H-ar), 7.42 (d, *J* = 8.4 Hz, 2H, 3,5-Har), 6.94 (s, 1H, 5-Hth), 3.99 (s, 2H, CH_2_OH), 3.68 (s, 1H, OH), 3.02 (t, *J* = 5.5 Hz, 2H, CH_2_), 2.12 (s, 3H 3,5,7-Had), 1.95 (d, *J* = 11.0 Hz, 6H, 2,8,9-Had), 1.83–1.73 (m, 6H, 4,6,10-Had). ^13^C NMR (150 MHz, CDCl_3_) *δ* 168.5 (2-Cth), 155.7 (4-Cth), 153.8 (1-Car), 130.7 (4-Car), 126.3 (2,6-Car), 125.5 (3,5-Car), 113.4 (5-Cth), 62.1 (CH_2_OH), 43.0 (2,8,9-Cad), 36.7 (4,6,10-Cad), 36.4 (1-Cad), 33.8 (CH_2_), 28.9 (3,5,7-Cad). Anal. calcd for C_21_H_25_NOS: C, 74.30; H, 7.42; N, 4.13 found C, 74.53; H, 7.31; N, 3.95.

#### 2-{2-[4-(1-Tricyclo[3.3.1.1^3,7^]decyl)phenyl]thiazol-4-yl)ethyl}methanesulfonate (**17**)

To a stirred solution of the alcohol **2g** (170 mg, 0.50 mmol) and Et_3_N (85 μL) in DCM (2 mL), MsCl (50 μL) was added dropwise at 0 °C. The reaction mixture was stirred at ambient temperature overnight. 1 M hydrochloride solution was added, into the reaction mixture, which was then extracted with EtOAc and the organic layer was dried over MgSO_4_ and the solvent evaporated to afford compound **17** as a yellow solid (170 mg, 95%), which used in the next step without further purification.

#### 2-[4-(1-Tricyclo[3.3.1.1^3,7^]decyl)phenyl]-4-(2-azidoethyl)thiazole (**2h**)

To a stirred solution of the mesylate **17** (180 mg, 0.42 mmol) in anhydrous DMF (2 mL), was added NaN_3_ (40 mL) and the reaction mixture was heated at 60 °C, for 2 h. The reaction mixture was then extracted with EtOAc and the organic layer was dried over MgSO_4_ and evaporated *in vacuo*. The resulting residue was then purified by column chromatography. Elution with 20% EtOAc in hexanes afforded compound **2h** as a white solid (100 mg, 65%). ^1^H NMR (400 MHz, CDCl_3_) *δ* 7.87 (d, *J* = 8.6 Hz, 2H, 2,6H-ar), 7.42 (d, *J* = 8.5 Hz, 2H, 3,5-Har), 6.99 (s, 1H, 5-Hth), 3.70 (t, *J* = 6.9 Hz, 2H, CH_2_N), 3.09 (t, *J* = 7.1 Hz, 2H, CH_2_), 2.12 (s, 3H, 3,5,7-Had), 1.93 (d, 6H, 2,8,9-Had), 1.85–1.71 (m, 6H, 4,6,10-Had). ^13^C NMR (75 MHz, CDCl_3_) *δ* 168.5 (2-Cth), 154.0 (4-Cth), 153.7 (1-Car) 131.1 (4-Car), 126.5 (2,6-Car), 125.6 (3,5-Car), 114.6 (5-Cth), 50.7 (CH_2_N_3_), 43.2 (2,8,9-Cad), 36.9 (4,6,10-Cad), 36.6 (1-Cad), 31.5 (CH_2_), 29.0 (3,5,7-Cad). Anal. calcd for C_21_H_24_N_4_S: C, 69.20; H, 6.64; N, 15.37 found C, 69.08; H, 6.47; N, 14.99.

#### 2-Bromo-1-[4-(1-Tricyclo[3.3.1.1^3,7^]decyl)phenyl]ethanone (**19**)

To a stirred solution of (1-phenyl)adamantane (**18**)^35^ (1.00 g, 4.71 mmol) and AlCl_3_ (700 mg, 5.10 mmol) in anhydrous DCM (10 mL) was added a solution of BrCOCH_2_Br (0.5 mL, 4.71 mmol) in anhydrous DCM (10 mL) under an argon atmosphere, at –10 °C. The reaction mixture was then heated to room temperature and stirred under an argon atmosphere overnight. Then the reaction mixture was poured onto ice-water, extracted with DCM and the organic layer was dried over MgSO_4_ and the solvent evaporated under vacuum. The resulting crude residue was purified by gradient column chromatography. Elution with EtOAc 3–5% in hexanes afforded compound **19** as a white solid (900 mg, 57%). M.p.: 94–96 °C (EtOAc/Hex). ^1^H NMR (400 MHz, CDCl_3_) *δ* 7.94 (d, *J* = 8.6 Hz, 2H, 2,6-Har), 7.48 (d, *J* = 8.6 Hz, 2H, 3,5-Har), 4.45 (s, 2H, CH_2_), 2.12 (s, 3H, 3,5,7-Had), 1.92 (d, *J* = 2.5 Hz, 6H, 2,8,9-Had), 1.83–1.75 (br.s, 6H, 4,6,8-Had). ^13^C NMR (75 MHz, CDCl_3_) *δ* 191.0 (C=O), 158.0 (1-Car), 129.0 (2,6-Cad), 127.5 (4-Cad), 125.6 (3,5-Car), 42.9 (2,8,9-Cad), 36.9 (1-Cad), 36.8 (3,6,10-Cad), 31.1 (CH_2_), 28.8 (3,5,7-Cad). Anal. calcd for C_18_H_21_BrO: C, 64.87; H, 6.35 found C, 64.63; H, 6.46.

#### 4-[4-(1-Tricyclo[3.3.1.1^3,7^]decyl)phenyl]thiazol-2-amine hydrobromide (**3a**)

A solution of the bromoketone **19** (130 mg, 0.39 mmol) and thiourea (30 mg, 0.39 mmol) in EtOH (3 mL) was heated at 80 °C, in an autoclave, overnight. The reaction mixture was then cooled to room temperature, treated with Et_2_O and the resulting precipitate was filtered to afford compound **3a** hydrobromide as a white solid (150 mg, 88%). M.p. (hydrobromide): 345 °C (dec) (EtOH/Et_2_O). ^1^H NMR (400 MHz, DMSO-*d*_6_) *δ* 8.84 (br.s, 3H, *N*H_3_), 7.66 (d, *J* = 8.5 Hz, 2H, 2,6-Har), 7.47 (d, *J* = 8.5 Hz, 2H, 3,5-Har), 7.18 (d, *J* = 5.1 Hz, 1H, 5-Hth), 2.07 (d, *J* = 8.1 Hz, 3H, 3,5,7-Had), 1.87 (br.s, 6H, 2,8,9-Had), 1.74 (s, 6H, 4,6,10-Had). ^13^C NMR (150 MHz, DMSO-*d*_6_) *δ* 170.1 (4-Cth), 152.2 (2-Cth), 127.2 (1-Car), 125.7 (4-Car), 125.6 (2.6-Car), 125.3 (3,5-Car), 101.9 (5-Cth), 42.3 (2,8,9-Cad), 36.1 (4,6,10-Cad), 35.9 (1-Cad), 28.2 (3,5,7-Cad). Anal. calcd for C_19_H_22_BrN_2_S: C, 58.31; H, 5.92; N, 7.16 found C, 58.12; H, 5.85; N, 6.98.

#### 2-{4-[4-(1-Tricyclo[3.3.1.1^3,7^]decyl)phenyl]thiazol-2-yl}methanamine difumarate (**3b**)

A solution of the bromoketone **19** (1.10 g, 3.30 mmol) and amide **20** (ref. 38) (750 mg, 3.60 mmol) in i-PrOH (12 mL) was heated at 120 °C, in an autoclave, overnight. The reaction mixture was then cooled to room temperature, treated with Et_2_O and the resulting precipitate was filtered to afford compound **3b** (480 mg, 36%). M.p. (difumarate): 172 °C (dec) (EtOH/Et_2_O). ^1^H NMR (400 MHz, DMSO-*d*_6_) *δ* 8.02 (s, 1H, 5-Hth), 7.90 (d, *J* = 8.5 Hz, 2H, 2,6-Har), 7.42 (d, *J* = 8.5 Hz, 2H, 3,5-Har), 6.57 (s, 4H, Hfum), 4.33 (s, 2H, CH_2_), 2.06 (s, 3H, 3,5,7-Had), 1.88 (d, *J* = 2.2 Hz, 6H, 2,8,9-Had), 1.74 (s, 6H, 4,6,10-Had). ^13^C NMR (75 MHz, DMSO-*d*_6_) *δ* 167.7 (2-Cth), 166.8 (C=O, fum), 151.0 (4-Cth), 134.5 (CH_2_fum), 131.4 (1-Car), 127.1 (4-Car), 125.9 (2,6-Car), 125.2 (3,5-Car), 114.5 (5-Cth), 42.6 (2,8,9-Cad), 40.8 (CH_2_N), 36.3 (4,6,10-Cad), 35.8 (1-Cad), 28.4 (3,5,7-Cad). Anal. calcd for C_28_H_32_N_2_O_8_S: C, 60.62; H, 5.79; N, 5.03 found C, 60.33; H, 6.02; N, 4.89.

#### 2-{4-[4-(1-Tricyclo[3.3.1.1^3,7^]decyl)phenyl]thiazol-2-yl}ethanamine hydrobromide (**3c**)

Ethanamine **3c** was prepared in a similar way as the amine **3b**, using the bromoketone **19** (800 mg, 2.40 mmol) and derivative **21** (ref. 7) (550 mg, 2.69 mmol) as starting materials to afford compound **3c** hydrobromide as a white solid (850 mg, 85%). M.p. (hydrobromide): 261–263 °C (EtOH/Et_2_O). ^1^H NMR (400 MHz, DMSO-*d*_6_) *δ* 7.98 (s, 1H, 5-Cth), 7.94–7.85 (m, 5H, *N*H_3,_ 2,6-Har), 7.42 (d, *J* = 8.4 Hz, 2H, 3,5-Har), 3.34–3.26 (m, 4H, *N*CH_2_, CH_2_), 2.07 (s, 3H, 3,5,7-Had), 1.88 (s, 6H, 2,8,9-Had), 1.74 (s, 6H, 4,6,10-Had). ^13^C NMR (150 MHz, DMSO-*d*_6_) *δ* 165.5 (4-Cth), 154.1 (2-Cth), 150.8 (1-Car), 125.9 (2,6-Car), 1245.0 (3,5-Car), 113.5 (5-Cth), 42.5 (2,8,9-Cad), 38.1 (NCH_2_), 36.1 (4,6,10-Cad), 35.7 (1-Cad), 30.3 (CH_2_), 28.3 (3,5,7-Cad). Anal. calcd for C_21_H_27_BrN_2_S: C, 60.14; H, 6.49; N, 6.68 found C, 60.38; H, 6.59; N, 6.45.

#### 2-{4-[4-(1-Tricyclo[3.3.1.1^3,7^]decyl)phenyl]thiazol-2-yl}-*N*,*N*-dimethylethan-1-amine dihydrochloride (**3d**)

Dimethylamine **3d** was prepared in a similar way as the amine **1c**, using the derivative **3c** (200 mg, 0.48 mmol) as starting material to afford compound **3d** as a white solid (150 mg, 85%). M.p. (dihydrochloride): 200–202 °C (EtOH/Et_2_O). ^1^H NMR (400 MHz, DMSO-*d*_6_) *δ* 10.84 (s, 1H, *N*HTh), 7.97 (br.s, 1H, 5-Hth), 7.88 (d, *J* = 8.4 Hz, 2H, 2,6-Har), 7.42 (d, *J* = 8.5 Hz, 2H, 3,5-Har), 5.16 (br.s, 1H, *N*H), 3.55 (s, 4H, CH_2_, *N*CH_2_), 2.83 (d, *J* = 4.9 Hz, 6H, 3,5,7-Had), 2.07 (s, 3H), 1.88 (d, *J* = 2.3 Hz, 6H, 2,8,9-Had), 1.74 (s, 6H, 4,6,10-Had). ^13^C NMR (150 MHz, DMSO-*d*_6_) *δ* 165.0 (4-Cth), 154.0 (2-Cth), 150.8 (1-Car), 131.3 (4-Car), 125.9 (2,6-Car), 125.0 (3,5-Car), 113.6 (5-Cth), 54.9 (*N*CH_2_), 42.5 (2,8,9-Cad), 42.2 (CH_3_), 36.2 (4,6,10-Cad), 35.7 (1-Cad), 28.3 (3,5,7-Cad), 27.6 (CH_2_). Anal. calcd for C_23_H_3_Cl_2_N_2_S: C, 62.86; H, 7.34; N, 6.37 found C, 62.72; H, 7.23; N, 6.11.

#### 1-{2-[4-(1-Tricyclo[3.3.1.1^3,7^]decyl)phenyl]thiazol-4-yl}guanidine (**3e**)

A stirred solution of the bromoketone **19** (200 mg, 0.60 mmol) and guanylthiourea (80 mg, 0.66 mmol) in EtOH (4 mL) was refluxed overnight. The reaction mixture was then cooled to room temperature, Et_2_O was added and the resulting suspension was filtered to afford compound **3e** hydrobromide (200 mg, 89%). M.p. (hydrobromide): 358–359 °C. ^1^H NMR (400 MHz, DMSO-*d*_6_) *δ* 11.98 (s, 1H, *N*H), 8.22 (br.s, 4H, 2 × *N*H_2_), 7.85 (d, *J* = 8.4 Hz, 2H, 2,6-Har), 7.68 (s, 1H, 5-Hth), 7.41 (d, *J* = 8.4 Hz, 2H, 3,5-Har), 2.06 (s, 3H, 3,5,7-Had), 1.87 (s, 6H, 2,8,9-Had), 1.74 (s, 6H, 4,6,10-Had). ^13^C NMR (75 MHz, DMSO-*d*_6_) *δ* 160.2 (2-Cth), 154.0 (4-Cth), 151.1 (1-Car), 130.9 (4-Car), 125.8 (2,6-Car) 125.1 (3,5-Car), 107.4 (5-Cth), 42.9 (2,8,9-Cad), 36.1 (4,6,10-Cad), 35.7 (1-Cad), 28.3 (3,5,7-Cad). Anal. calcd for C_20_H_25_BrN_4_S: C, 55.43; H, 5.81; N, 12.93 found C, 55.31; H, 5.89; N, 13.23.

#### N-(2-(2-Phenylthiazol-4-yl)ethyl)(1-tricyclo[3.3.1.1^3,7^]decane)carboxamide (**4a**)

A solution of 1-adamantylcarbonyl chloride (450 mg, 2.26 mmol) in anhydrous THF (8 mL) was added dropwise, at 0 °C onto a stirred solution of the 2-phenylthiazol-4-ethylamine (**22**)^6^ (308 mg, 1.51 mmol) and Et_3_N (0.45 mL, 3.23 mmol) in THF (8 mL) and the reaction mixture was stirred at ambient temperature under an argon atmosphere overnight. The mixture was extracted with DCM and the organic phase was then washed with water, dried over MgSO_4_ and the solvent evaporated under reduced pressure. The resulting residue was purified with column chromatography. Elution with 50% EtOAc in hexanes afforded compound **4a** as a white solid (270 mg, 49%). M.p.: 135–136 °C. ^1^H NMR (400 MHz, CDCl_3_) *δ* 7.99–7.91 (m, 2H, 2,6-Har), 7.46–7.38 (m, 3H, 3,4,5-Har), 6.96 (s, 1H, 5-Cth), 6.86 (br.s, 1H, *N*H), 3.60 (dd, *J* = 12.0, 5.6 Hz, 2H, CH_2_*N*H), 2.91 (t, *J* = 9,51 Hz 2H, CH_2_), 2.05 (br.s, 3H, 3,5,7-Had), 1.88 (s, 6H, 2,8,9-Had), 1.70 (q, *J* = 12.1 Hz, 6H 4,6,10-Had). ^13^C NMR (150 MHz, CDCl_3_) *δ* 177.88 (C=O), 168.2 (2-Cth), 155.9 (4-Cth), 133.5 (1-Car), 130.0 (4-Car), 128.8 (2,6-Car), 126.3 (3,5-Car), 114.2 (5-Cth), 40.5 (CH_2_N), 39.3 (2,8,9-Cad), 38.8 (1-Cad), 36.5 (4,6–10-Cad), 30.7 (CH_2_), 28.1 (3,5,7-Cad). Anal. calcd for C_22_H_26_N_2_OS: C, 72.08; H, 7.15; N, 7.64 found C, 72.31; H, 7.09; N, 7.88.

#### 1-((1*R*,4*R*)-7,7-Dimethyl-2-oxobicyclo[2.2.1]hept-1-yl)-*N*-(2-(2-phenylthiazol-4-yl)ethyl)methanesulfonamide (**4b**)

The carboxamide **4b** was prepared in a similar way as the derivative **4a**, using 2-phenylthiazol-4-ethylamine (**22**)^6^ (339 mg, 1.66 mmol) and (±)-10-camphorsulfonyl chloride (623 mg, 2.49 mmol) as starting materials in DCM (7 mL), to afford **4b** as a viscous liquid (350 mg, 50%). M.p (hydrochloride): 144–145 °C. ^1^H NMR (400 MHz, DMSO-*d*_6_) *δ* 7.93 (dd, *J* = 7.3, 2.2 Hz, 2H, 2,6-Har), 7.54–7.43 (m, 4H, 3,4,5-Har, 5-Hth), 7.24 (br.s, 1H, *N*H), 3.45–3.32 (m, 3H, CH_2_*N*H), 3.26 (d, *J* = 14.9 Hz, 1H, CH_2_S), 2.97 (t, *J* = 7.2 Hz, 2H, CH_2_), 2.85 (d, *J* = 14.9 Hz, 1H, CH_2_S), 2.30 (m, 2H, 3-Hcam_exo_, 6-Hcam_exo_), 2.00 (t, *J* = 4.4 Hz, 1H, 5-Hcam), 1.95–1.81 (m, 2H, 4-Hcam_exo_, 6-Hcam_endo_), 1.50 (ddd, *J* = 13.7, 9.3, 4.5 Hz, 1H, 3-Hcar_endo_), 1.39–1.30 (m, 1H, 4-Hcar_endo_), 0.96 (s, 3H, CH_3_), 0.73 (s, 3H, CH_3_). ^13^C NMR (150 MHz, DMSO-*d*_6_) *δ* 215.0 (C=O), 167.2 (2-Cth), 155.0 (4-Cth), 133.4 (1-Car), 130.7 (4-Car), 129.7 (2,6-Car), 126.6 (3,5-Car), 116.3 (5-Cth), 58.3 (2-Ccam), 48.2 (CH_2_), 48.0 (7-Ccam), 42.7 (6-Ccam), 42.5 (5-Ccam), 42.5 (CH_2_N), 32.2 (CH_2_), 26.7 (4-Ccam), 25.0 (3-Ccam), 19.8 (CH_3_), 19.7 (CH_3_). Anal. calcd for C_21_H_27_ClN_2_O_3_S: C, 55.43; H, 5.98; N, 6.16 found C, 55.21; H, 6.11; N, 6.02.

#### *N*-[2-(4-Phenylthiazol-2-yl)ethyl](1-tricyclo[3.3.1.1^3,7^]decane)carboxamide (**4c**)

To a stirred solution of the 4-phenylthiazol-2-ethylamine hydrobromide (**23**)^7^ (300 mg, 1.05 mmol) in DMF/DCM 1 : 1 (10 mL), was added 1-adamantanecarboxylic acid (227 mg, 1.26 mmol), HBTU (478 mg, 1.26 mmol), and DIPEA (474 mg, 3.68 mmol) and the reaction mixture was stirred at ambient temperature under an argon atmosphere, overnight. The mixture was then partitioned between DCM and an aqueous solution of citric acid (10%) and the aqueous phase was extracted with DCM. The combined organic phase was then washed with water and brine, dried over MgSO_4_ and the solvent evaporated under reduced pressure. The resulting residue was purified with gradient column chromatography. Elution with 10% to 50% EtOAc in hexanes afforded compound **4a** as a white solid (330 mg, 94%). M.p.: 107–108 °C. ^1^H NMR (400 MHz, CDCl_3_) *δ* 8.01–7.84 (m, 2H, 2,6-Har), 7.54–7.28 (m, 3,4,5-Har, 5-Hth), 6.92 (s, 1H, *N*H), 3.69 (dd, *J* = 11.8, 5.7 Hz, 3H, CH_2_N), 3.20 (t, *J* = 7.4 Hz, 3H, CH_2_), 2.02 (s, 3H, 3,5,7-Had), 1.87 (d, *J* = 2.3 Hz, 6H, 2,8,9-Had), 1.70 (q, *J* = 12.2 Hz, 6H, 4,6,10-Had).^13^C NMR (150 MHz, CDCl_3_) *δ* 177.9 (C=O), 168.2 (2-Cth), 155.9 (4-Cth), 133.5 (1-Car), 130.0 (4-Car), 128.8 (2,6-Car), 126.3 (3,5-Car), 114.2 (5-Cth), 40.5 (CH_2_N), 39.3 (2,8,9-Cad), 38.8 (1-Cad), 36.5 (4,6–10-Cad), 30.7 (CH_2_), 28.1 (3,5,7-Cad). Anal. calcd for C_22_H_26_N_2_OS: C, 72.09; H, 7.15; N, 7.64 found C, 72.27; H, 7.23; N, 7.92.

#### 1-((1*R*,4*R*)-7,7-Dimethyl-2-oxobicyclo[2.2.1]hept-1-yl)-*N*-(2-(4-phenylthiazol-2-yl)ethyl)methanesulfonamide hydrochloride (**4d**)

The sulfonamide **4d** was prepared in a similar way as the derivative **4b**, using 4-phenylthiazol-2-ethylamine (**23**)^7^ (210 mg, 1.03 mmol) and (±)-10-camphorsulfonyl chloride (400 mg, 1.59 mmol) as starting materials, to afford compound **4d** as a viscous liquid (300 mg, 70%). M.p. (hydrochloride): 130–131 °C. ^1^H NMR (400 MHz, DMSO-*d*_6_) *δ* 8.01 (s, 1H, 5-Hth), 7.95 (d, *J* = 7.5 Hz, 2H, 2,6-Har), 7.54–7.20 (m, 3H, 3,4,5-Har), 5.26 (s, 2H, *N*H), 3.46 (s, 2H, CH_2_N), 3.30 (d, *J* = 14.9 Hz, 1H, CH_2_S), 3.24 (t, *J* = 6.9 Hz, 2H, CH_2_), 2.90 (d, *J* = 14.9 Hz, 1H, CH_2_S), 2.37–2.25 (m, 2H, 3-Hcam_endo_, 6-Hcam_exo_), 2.01 (t, *J* = 4.4 Hz, 1H, 5-Hcam), 1.95–1.83 (m, 2H, 4-Hcam_exo_, 6-Hcam_endo_), 1.51 (ddd, *J* = 13.7, 9.3, 4.6 Hz, 1H, 3-Hcar_endo_), 1.41–1.28 (m, 1H, 4-Hcar_endo_), 0.97 (s, 3H, CH_3_), 0.74 (s, 3H, CH_3_). ^13^C NMR (150 MHz, DMSO-*d*_6_) *δ* 214.5 (C=O), 167.4 (2-Cth), 153.7 (4-Cth), 134.0 (1-Car), 128.7 (2,6-Car), 128.0 (4-Car), 126.0 (3,5-Car), 114.0 (5-Cth), 57.8 (2-Ccam), 47.9 (CH_2_S), 47.6 (7-Ccam), 42.3 (6-Ccam), 42.1 (5-Ccam), 42.0 (CH_2_N), 33.6 (CH_2_), 26.3 (4-Ccam), 24.5 (3-Ccam), 19.3 (CH_3_), 19.2 (CH_3_). Anal. calcd for C_21_H_27_ClN_2_O_3_S: C, 55.43; H, 5.98; N, 6.16 found C, 55.67; H, 5.77, N; 6.23.

## Conflicts of interest

There are no conflicts to declare.

## Supplementary Material

Supplementary information
