# Supplementary material for: Synthesis and evaluation of novel 2,4-disubstituted arylthiazoles against T. brucei
Source: RSC Med Chem. 2019 Dec 19;11(1):72–84. doi: 10.1039/c9md00478e (PMC7522794; doi:10.1039/c9md00478e)
Supplement: Supplementary file 1 [file MD-011-C9MD00478E-s001.pdf]

Electronic Supplementary Material (ESI) for MedChemComm.
This journal is © The Royal Society of Chemistry 2019

Synthesis and evaluation of novel 2,4-disubstituted arylthiazole derivatives against *T.brucei*

Markos-Orestis Georgiadis,[a] Violeta Kourbeli,[a] Ioannis P. Papanastasiou,*[a] Andrew Tsotinis,[a] Martin C. Taylor [b] and John M. Kelly [b]

[a]*School of Health Sciences, Department of Pharmacy, Division of Pharmaceutical Chemistry, National and Kapodistrian University of Athens, Panepistimioupoli-Zografou, 157 84 Athens, Greece*
[b]*Department of Pathogen Molecular Biology, London School of Hygiene and Tropical Medicine, Keppel Street, London*

Supporting Information

^{1}HNMR and ^{13}C NMR spectra

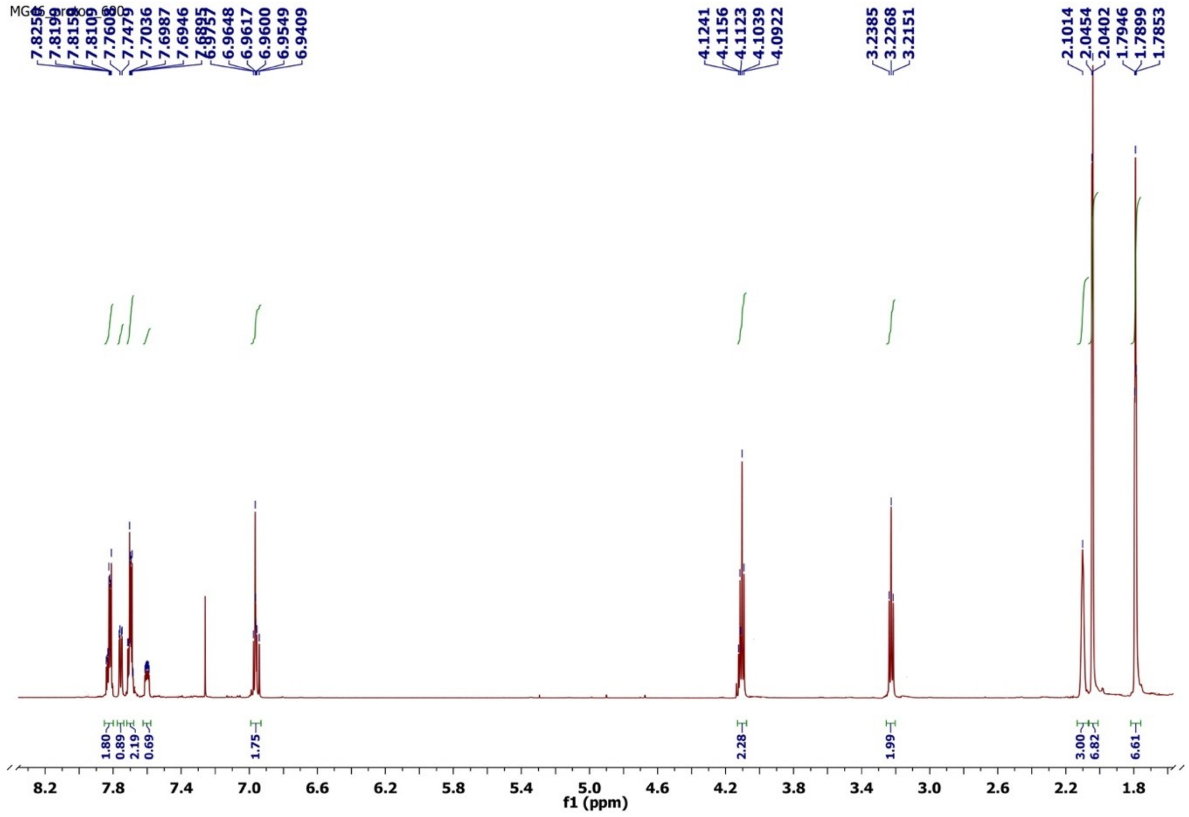

^{1}H NMR (400 MHz, $CDCl_3$) of 2-{2-[3-(1-tricyclo[3.3.1.1^{3,7}]decyl)-4-fluorophenyl)thiazol-4-yl]ethyl}isoindoline-1,3-dione (**8**)

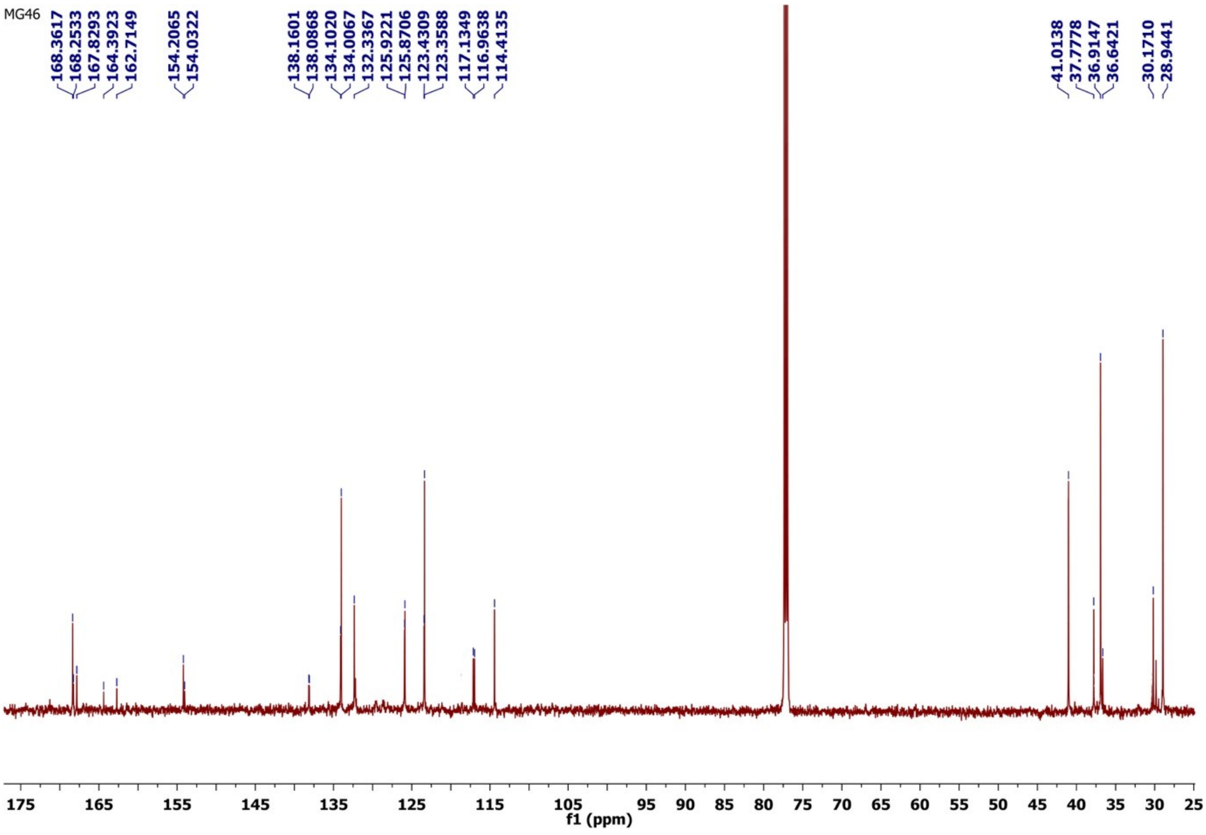

^{13}C NMR (150 MHz, $CDCl_3$) of 2-{2-[3-(1-tricyclo[3.3.1.1^{3,7}]decyl)-4-fluorophenyl)thiazol-4-yl]ethyl}isoindoline-1,3-dione (**8**)

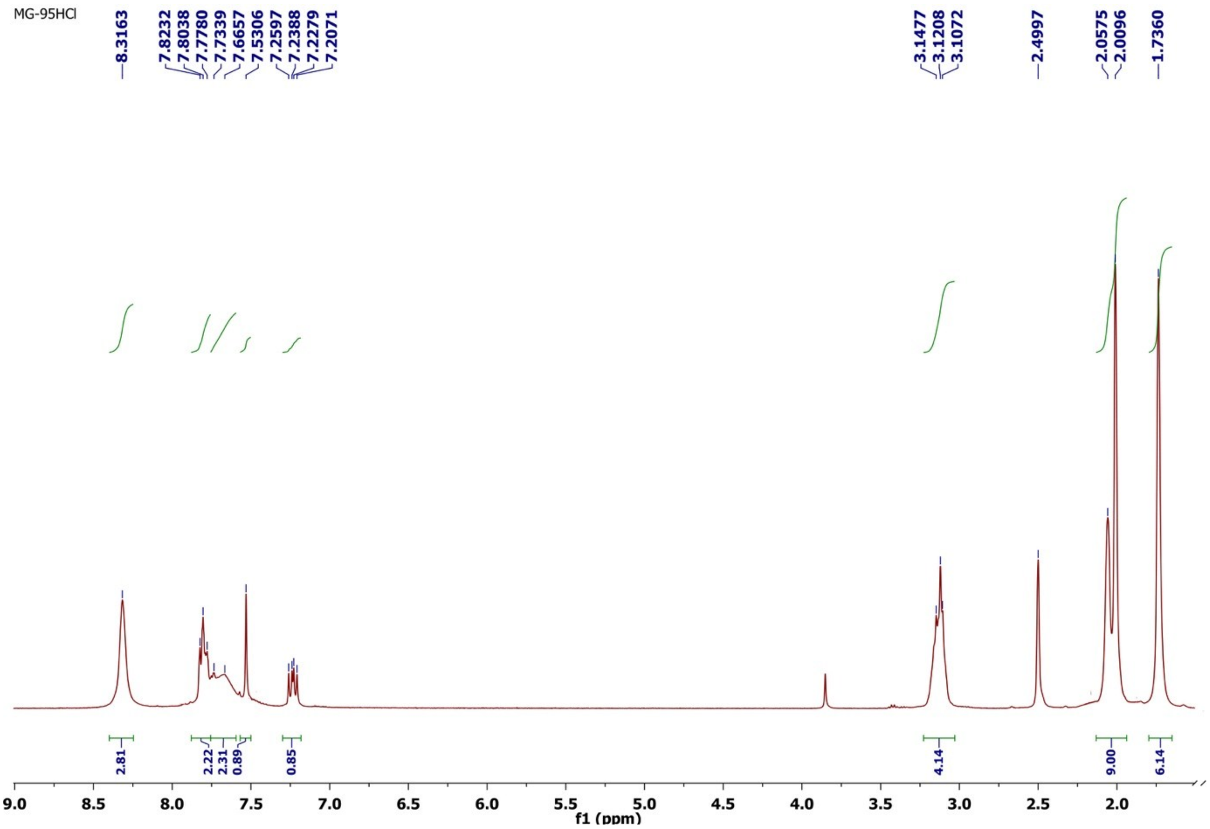

^{1}H NMR (600 MHz, DMSO-d_6) of 2-{2-[3-(1-tricyclo[3.3.1.1^{3,7}]decyl)-4-fluorophenyl]thiazol-4-yl}ethan-1-amine dihydrochloride (**1a**)

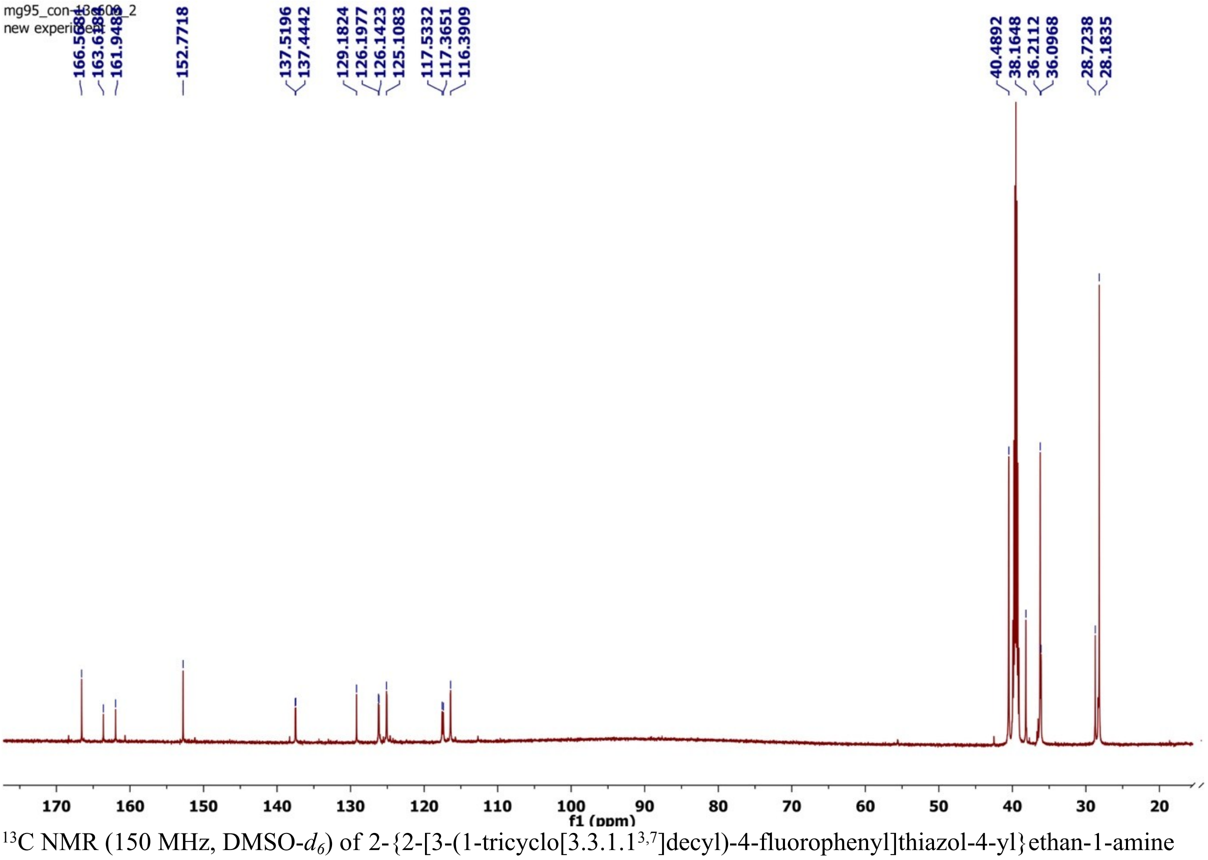

^{13}C NMR (150 MHz, DMSO-d_6) of 2-{2-[3-(1-tricyclo[3.3.1.1^{3,7}]decyl)-4-fluorophenyl]thiazol-4-yl}ethan-1-amine dihydrochloride (**1a**)

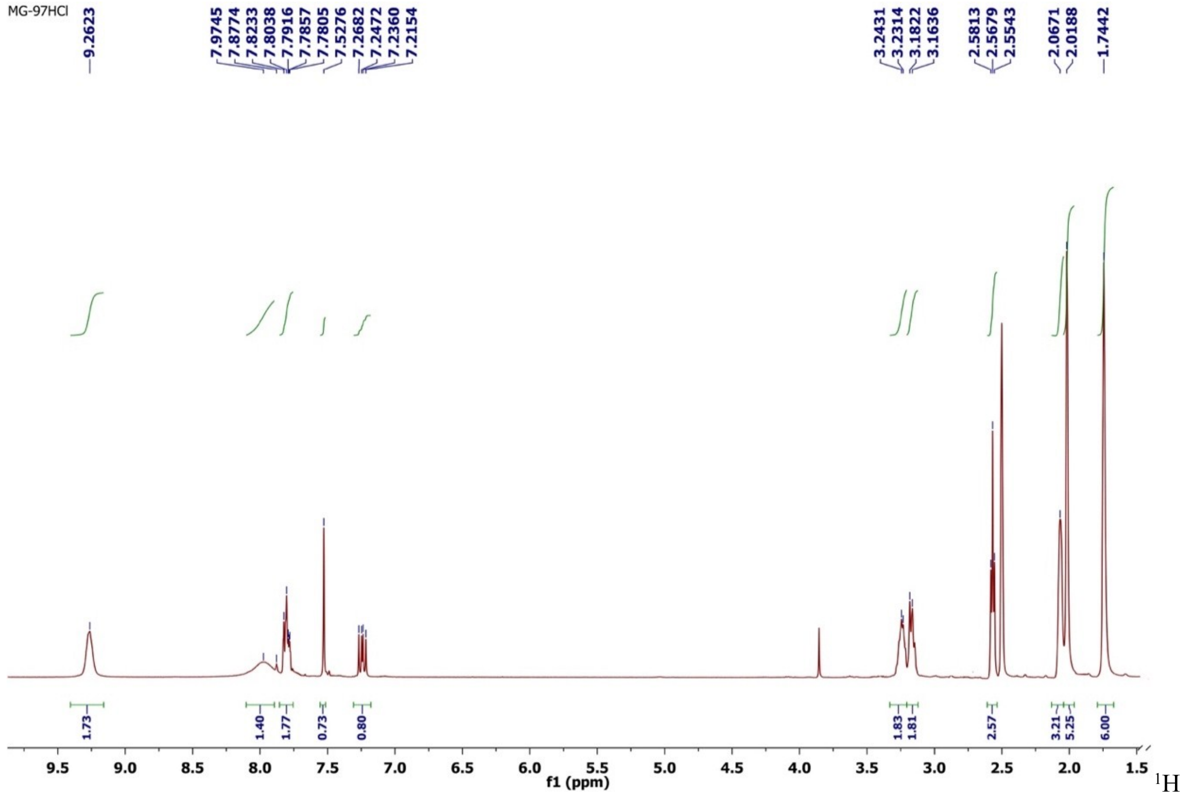

^{1}H NMR (400 MHz, DMSO-d_6) of 2-{2-[3-(1-tricyclo[3.3.1.1^{3,7}]decyl)-4-fluorophenyl]thiazol-4-yl}*N*-methylethan-1-amine dihydrochloride (**1b**)

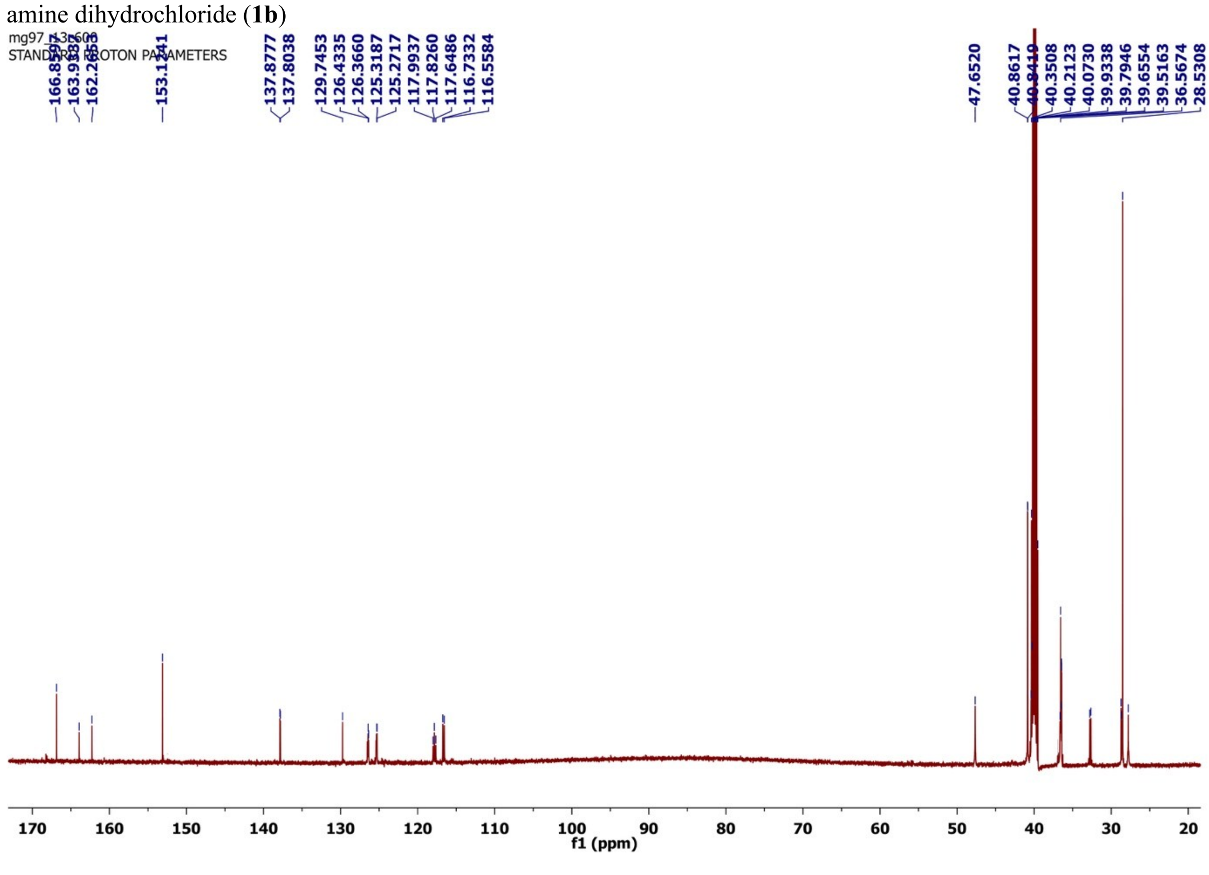

^{13}C NMR (150 MHz, DMSO-d_6) of of 2-{2-[3-(1-tricyclo[3.3.1.1^{3,7}]decyl)-4-fluorophenyl]thiazol-4-yl}*N*-methylethan-1-amine dihydrochloride (**1b**)

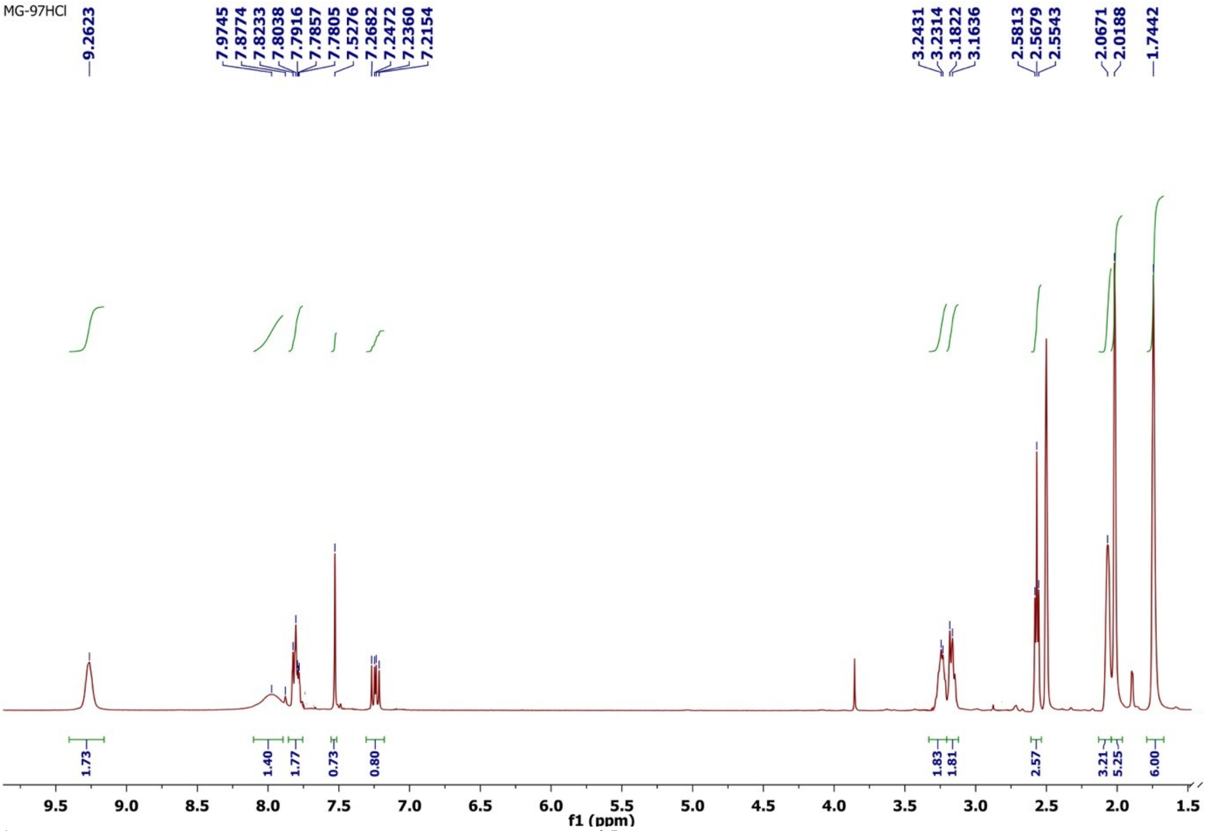

^{1}H NMR (400 MHz, DMSO-d_6) of 2-{2-[3-(1-tricyclo[3.3.1.1^{3,7}]decyl)-4-fluorophenyl]thiazol-4-yl}-*N,N*-dimethylethyl-1-amine dihydrochloride (**1c**)

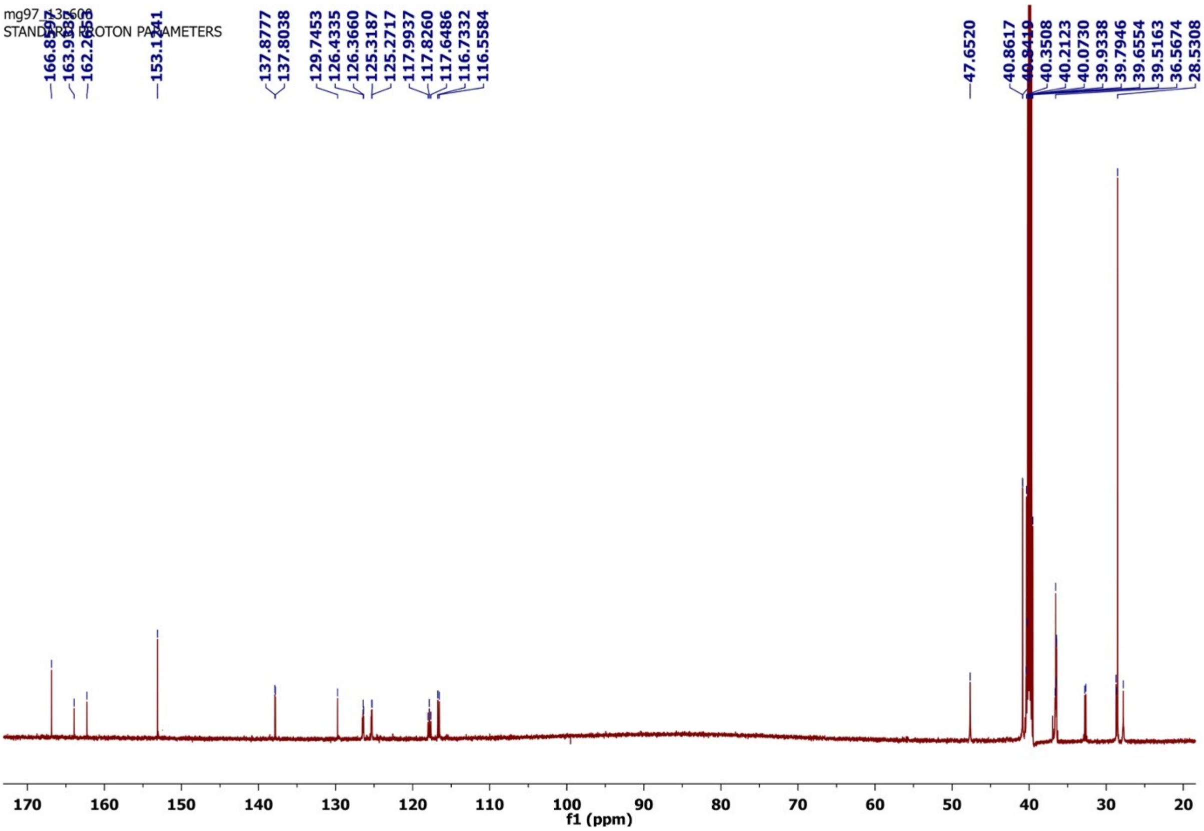

^{13}C NMR (150 MHz, DMSO-d_6) of 2-{2-[3-(1-tricyclo[3.3.1.1^{3,7}]decyl)-4-fluorophenyl]thiazol-4-yl}-*N,N*-dimethylethyl-1-amine dihydrochloride (**1c**)

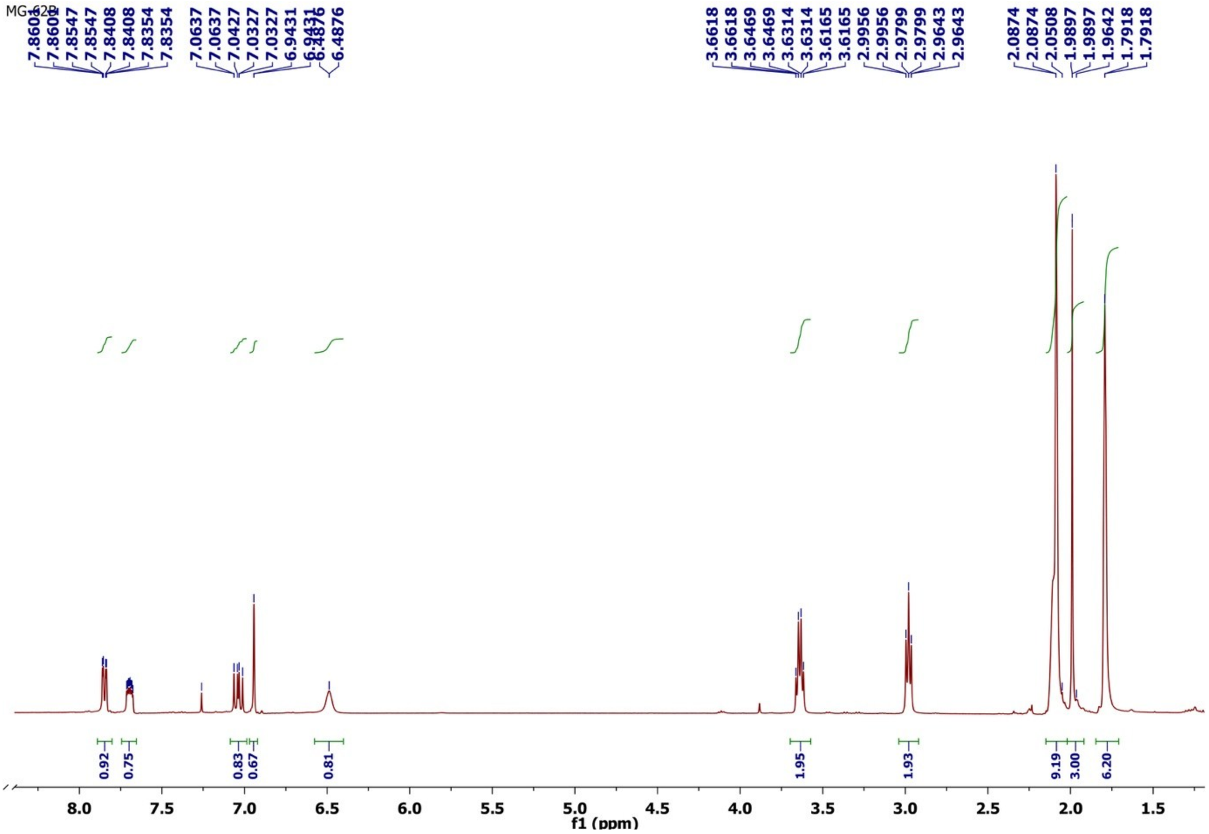

^{1}H NMR (400 MHz, $CDCl_3$) of *N*-{2-[2-(3-(1-tricyclo[3.3.1.1^{3,7}]decyl)-4-fluorophenyl]thiazol-4-yl)ethyl}acetamide (**1d**)

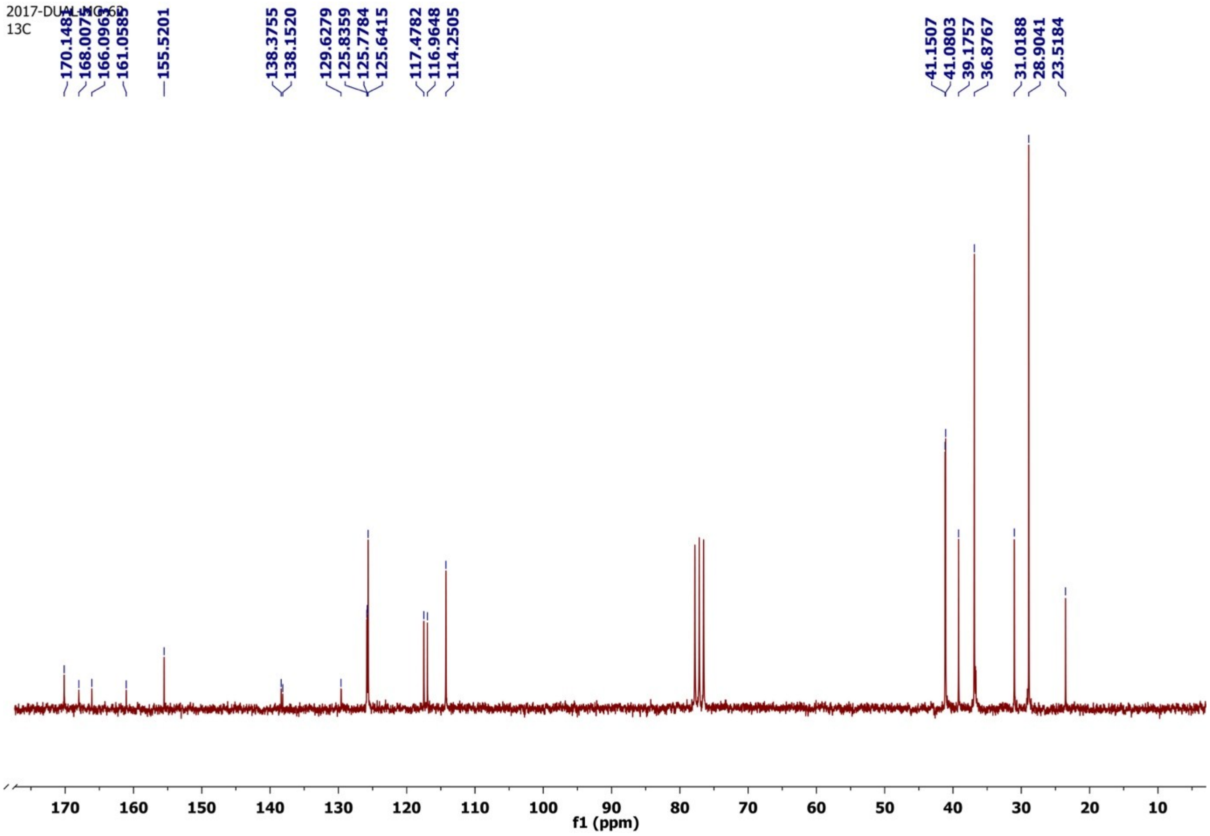

^{13}C NMR (50 MHz, $CDCl_3$) of *N*-{2-[2-(3-(1-tricyclo[3.3.1.1^{3,7}]decyl)-4-fluorophenyl]thiazol-4-yl)ethyl}acetamide (**1d**)

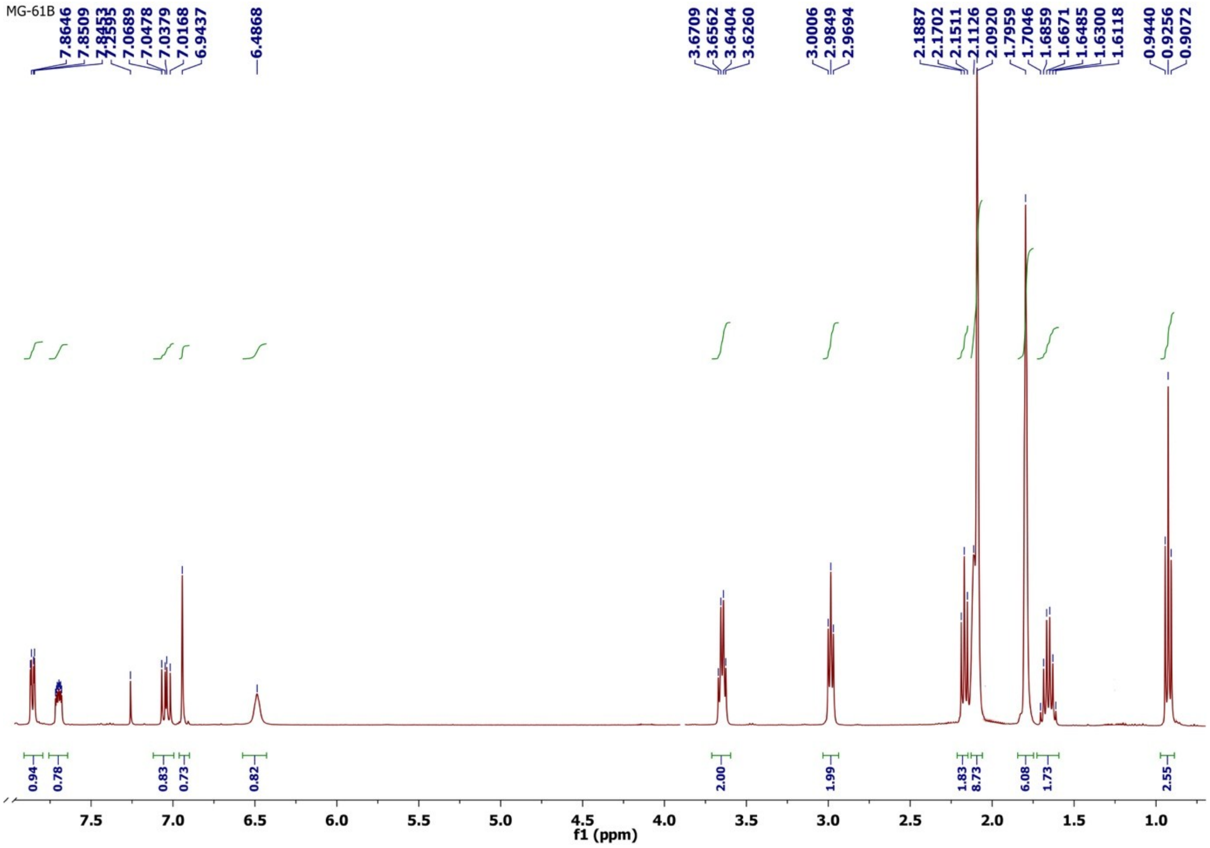

^{1}H NMR (400 MHz, $CDCl_3$) of *N*-{2-[2-(3-(1-tricyclo[3.3.1.1^{3,7}]decyl)-4-fluorophenyl)thiazol-4-yl]ethyl}butylamide (**1e**)

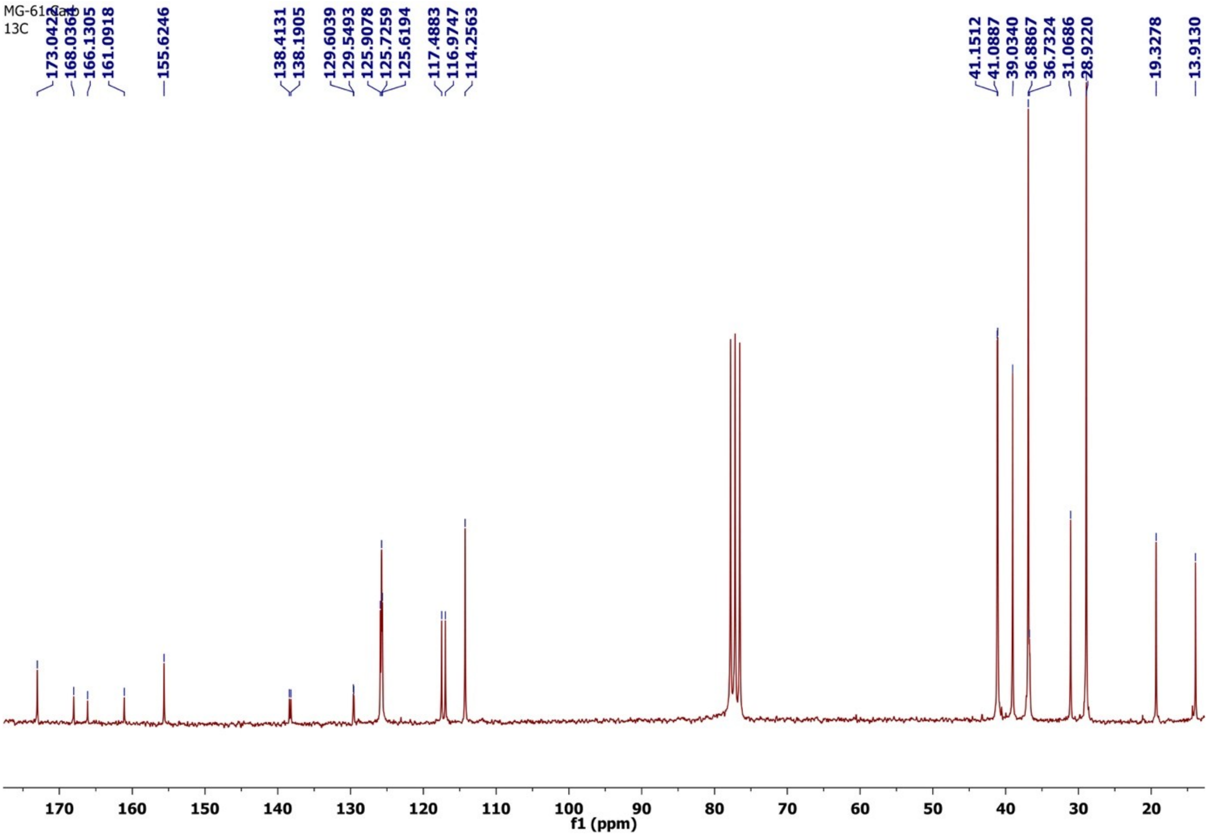

^{13}C NMR (50 MHz, $CDCl_3$) of *N*-{2-[2-(3-(1-tricyclo[3.3.1.1^{3,7}]decyl)-4-fluorophenyl)thiazol-4-yl]ethyl}butylamide (**1e**)

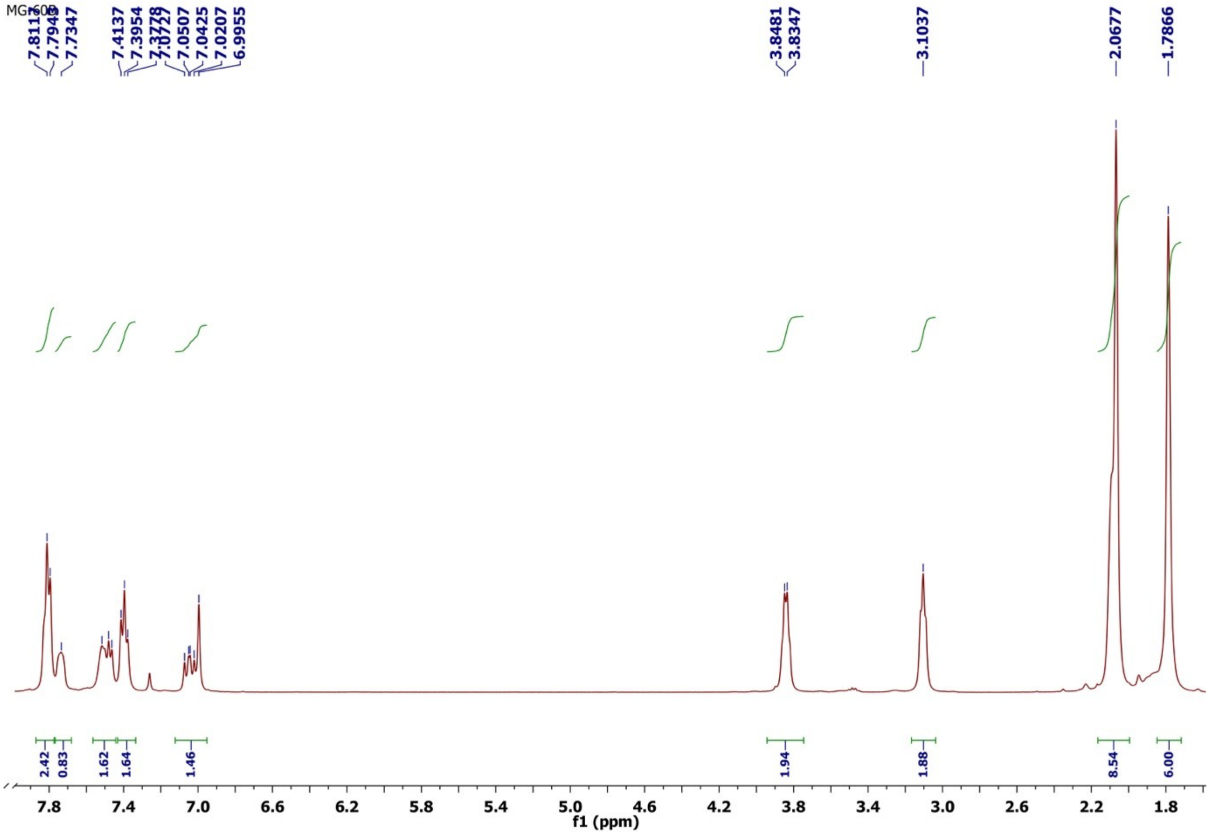

^{1}H NMR (400 MHz, $CDCl_3$) of *N*-{2-[2-(3-(1-tricyclo[3.3.1.1^{3,7}]decyl)-4-flueorophenyl)thiazol-4-yl]ethyl} benzamide (**1f**)

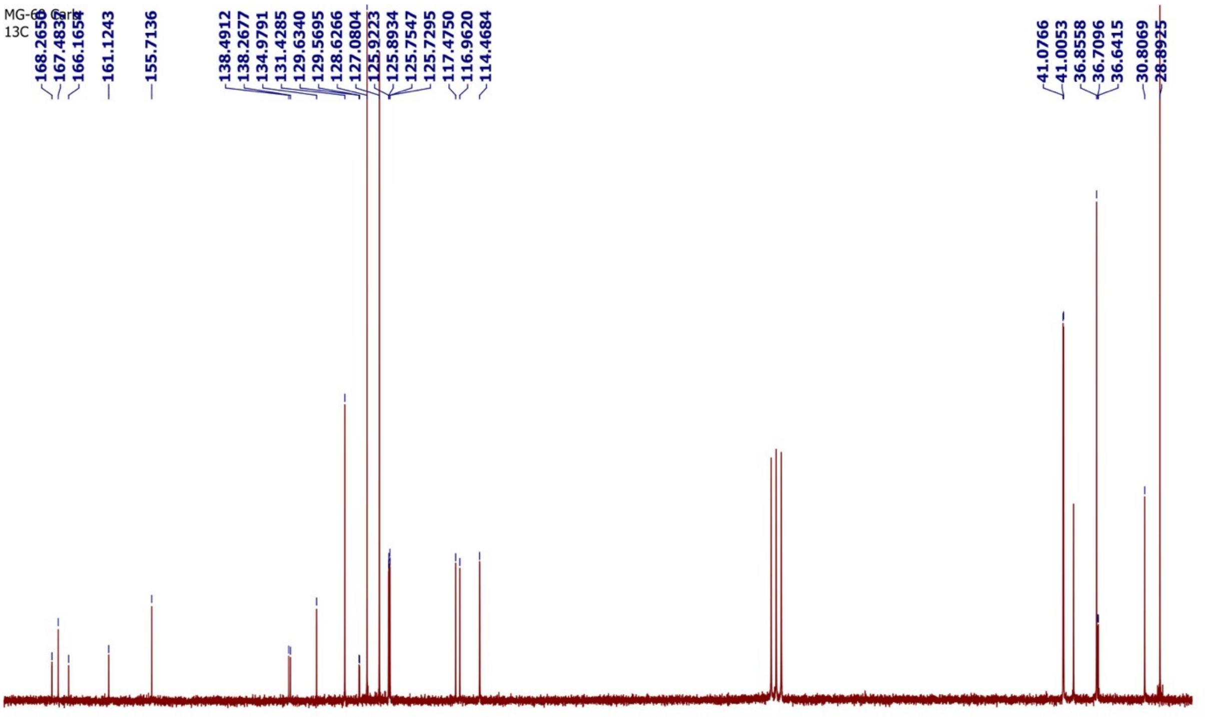

^{13}C NMR (50 MHz, $CDCl_3$) of *N*-{2-[2-(3-(1-tricyclo[3.3.1.1^{3,7}]decyl)-4-flueorophenyl)thiazol-4-yl]ethyl} benzamide (**1f**)

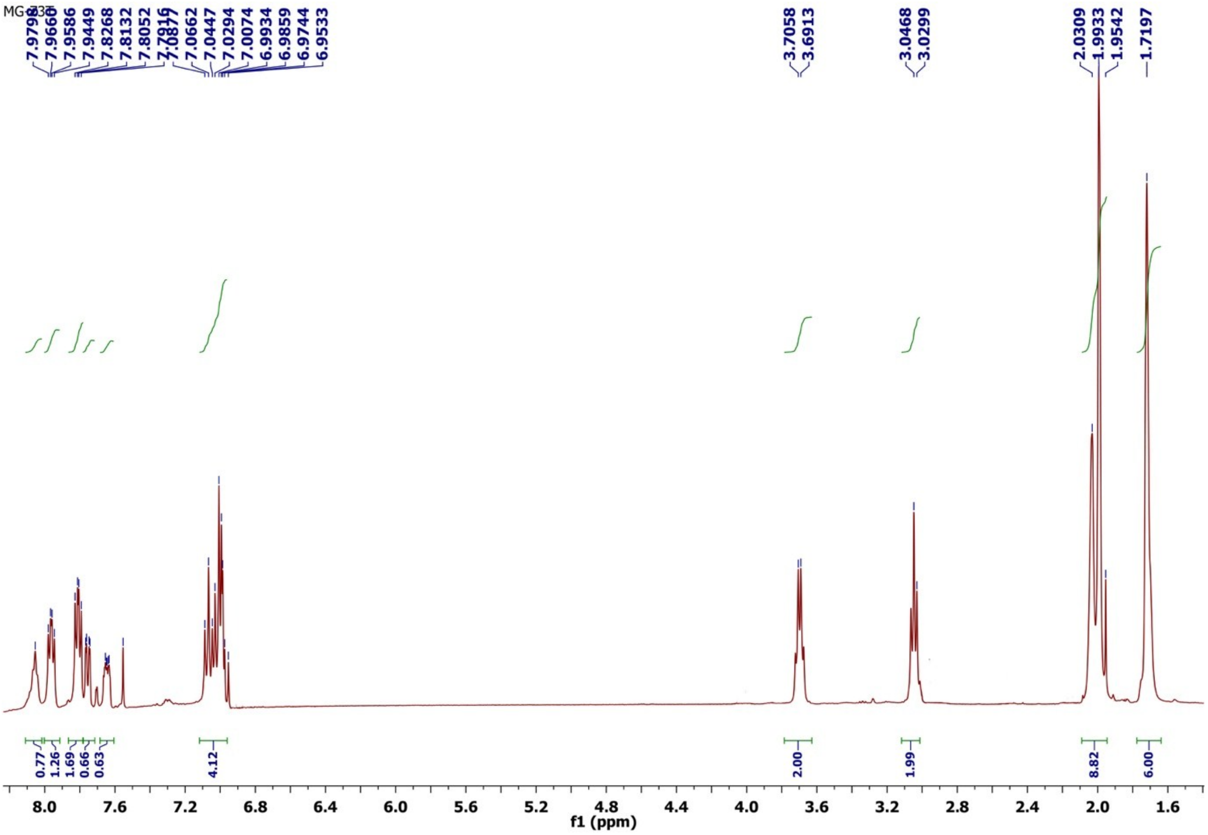

^{1}H NMR (400 MHz, $CDCl_3$) of *N*-{2-[2-(3-(1-tricyclo[3.3.1.1^{3,7}]decyl)-4-fluorophenyl)thiazol-4-yl]ethyl}-4-fluoro-benzamide (**1g**)

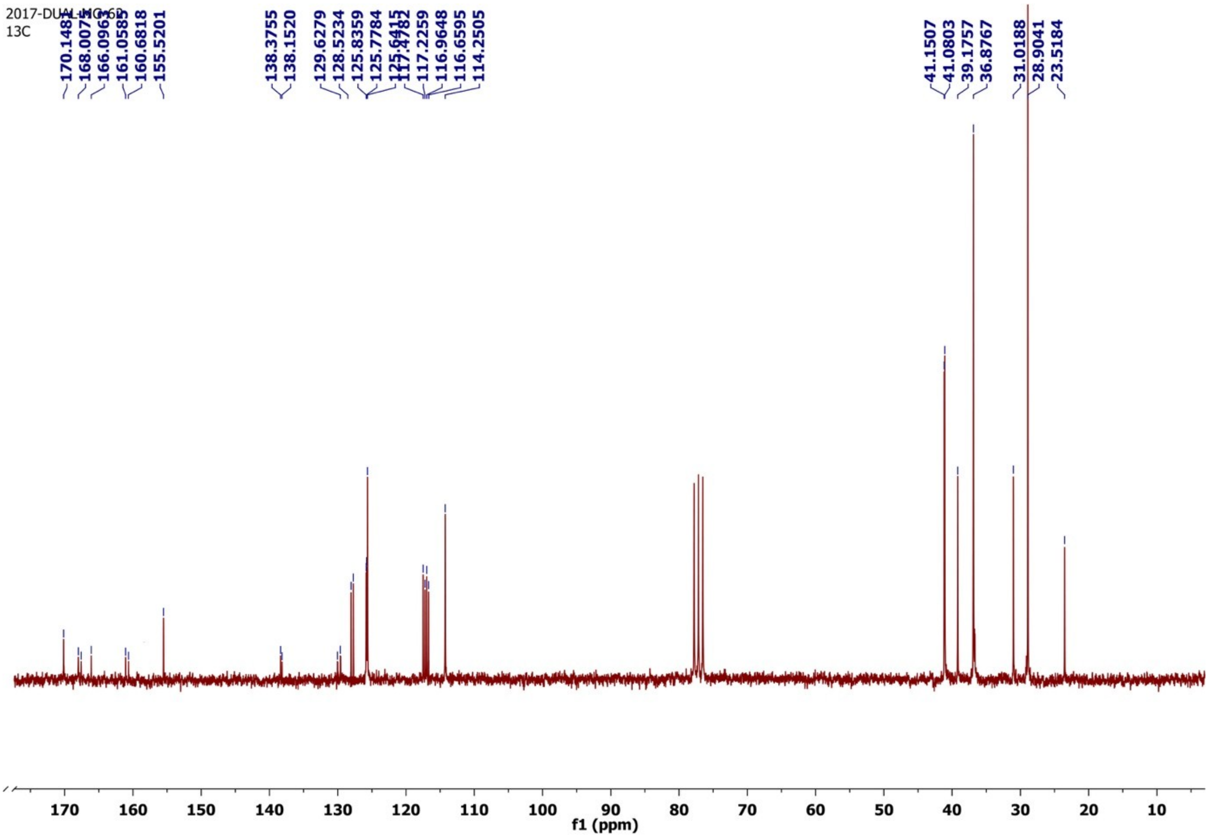

^{13}C NMR (50 MHz, $CDCl_3$) of *N*-{2-[2-(3-(1-tricyclo[3.3.1.1^{3,7}]decyl)-4-fluorophenyl)thiazol-4-yl]ethyl}-4-fluoro-benzamide (**1g**)

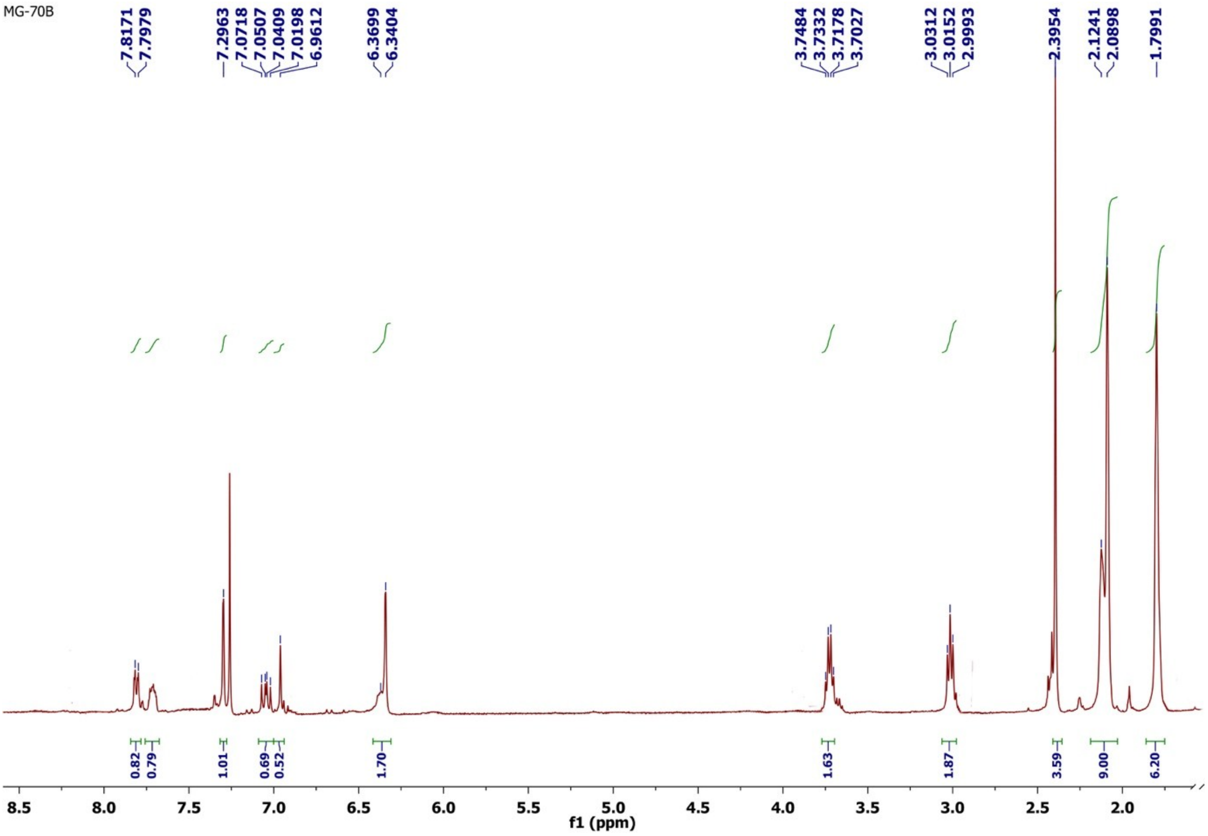

^{1}H NMR (400 MHz, $CDCl_3$) of *N*-{2-[2-(3-(1-tricyclo[3.3.1.1^{3,7}]decyl)-4-fluorophenyl)thiazol-4-yl]ethyl}-3-methyl-furan-2-ylcarboxamide (**1h**)

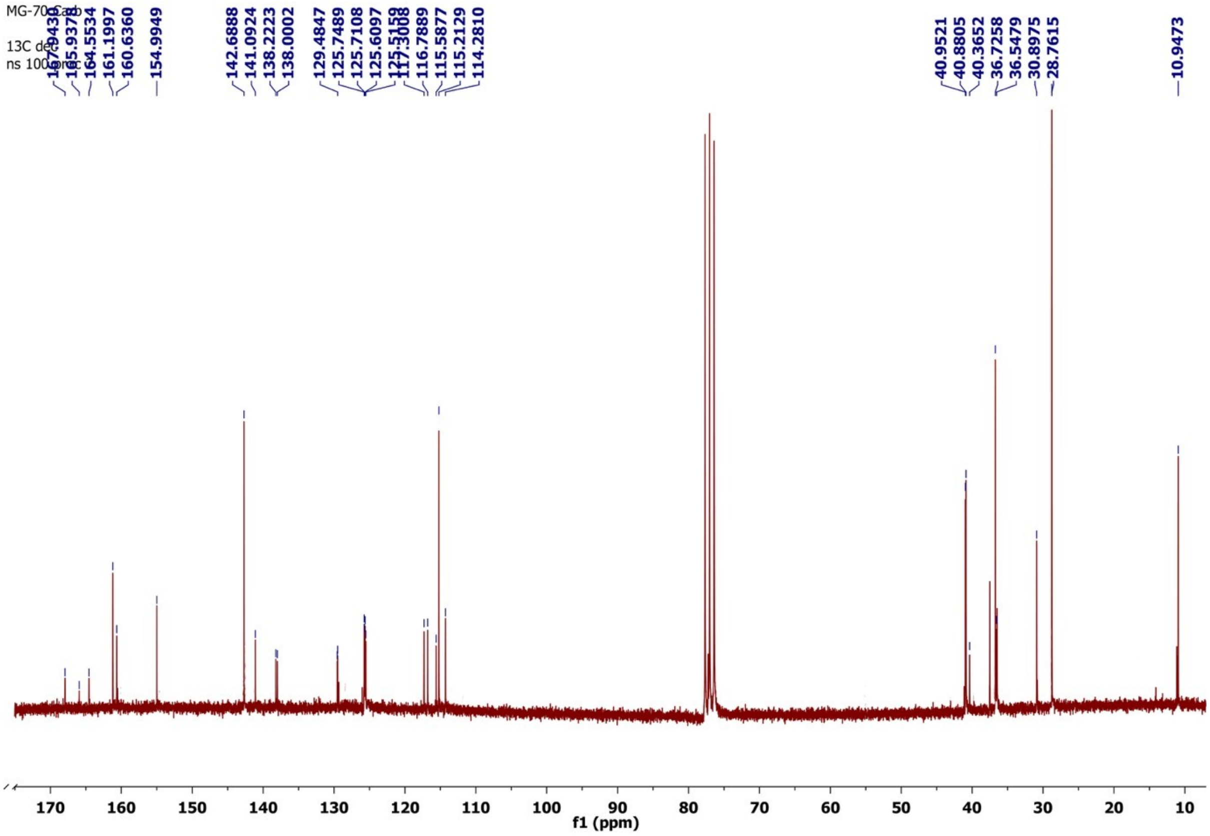

^{13}C NMR (50 MHz, $CDCl_3$) of *N*-{2-[2-(3-(1-tricyclo[3.3.1.1^{3,7}]decyl)-4-fluorophenyl)thiazol-4-yl]ethyl}-3-methyl-furan-2-ylcarboxamide (**1h**)

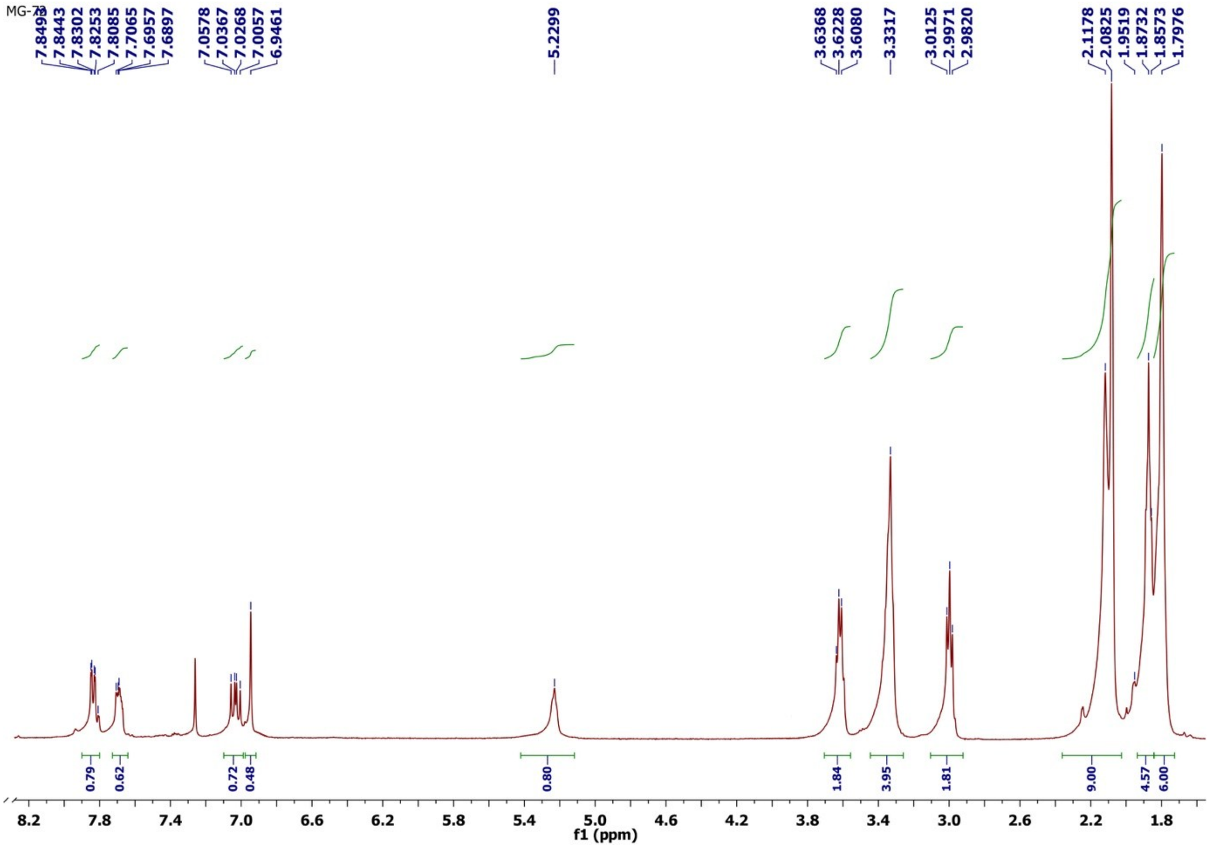

^{1}H NMR (400 MHz, $CDCl_3$) of *N*-{2-[2-(3-(1-tricyclo[3.3.1.$1^{3,7}$]decyl)-4-fluorophenyl)thiazol-4-yl]ethyl}pyrrolidin-1-ylcarboxamide (**1i**)

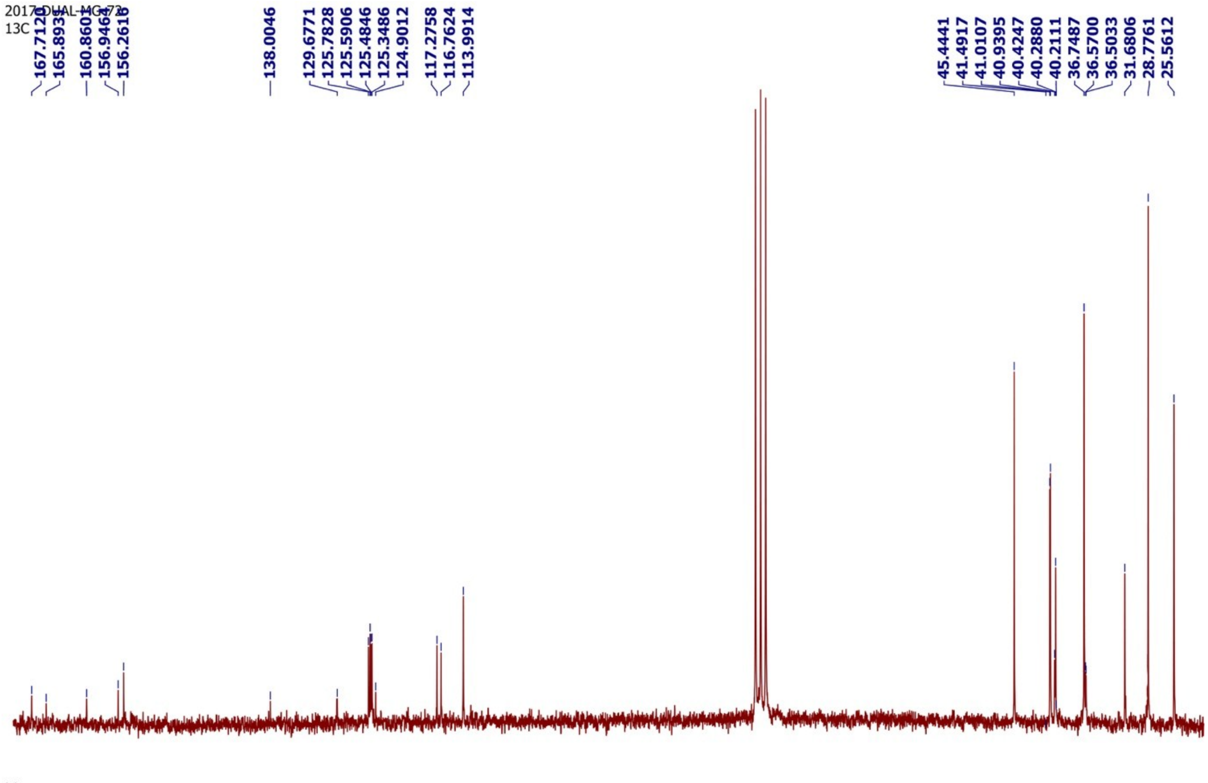

^{13}C NMR (50 MHz, $CDCl_3$) of *N*-{2-[2-(3-(1-tricyclo[3.3.1.$1^{3,7}$]decyl)-4-fluorophenyl)thiazol-4-yl]ethyl}pyrrolidin-1-ylcarboxamide (**1i**)

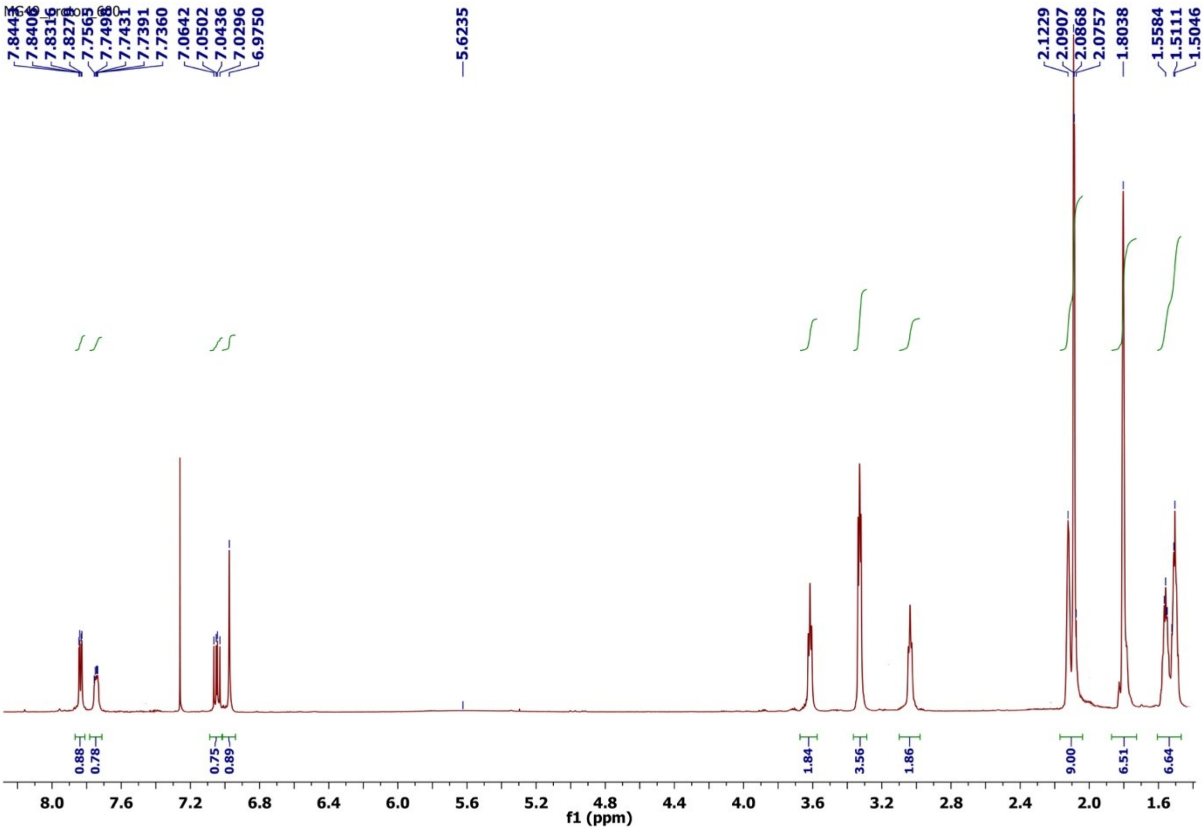

^{1}H NMR (400 MHz, $CDCl_3$) of *N*-{2-[2-(3-(1-tricyclo[3.3.1.1^{3,7}]decyl)-4-fluorophenyl)thiazol-4-yl]ethyl}piperidine-1-ylcarboxamide (**1j**)

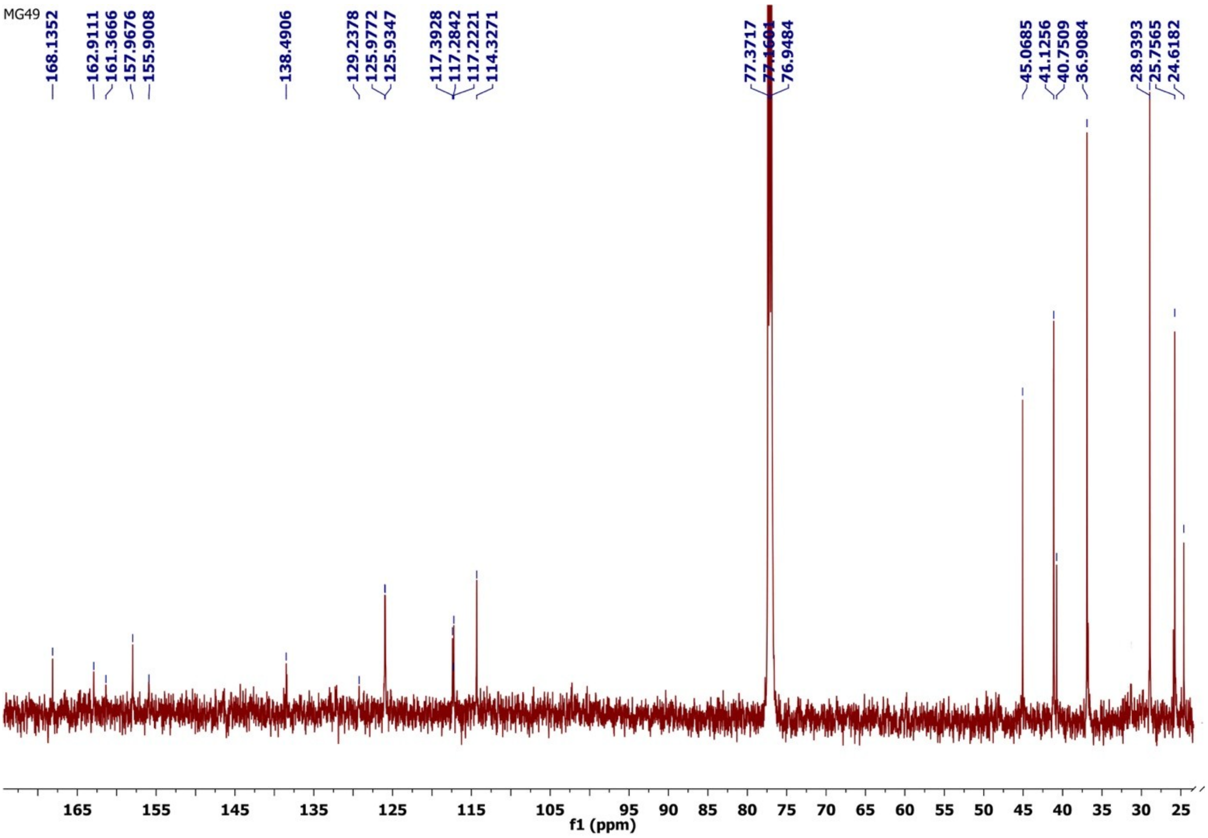

^{13}C NMR (150 MHz, $CDCl_3$) of *N*-{2-[2-(3-(1-tricyclo[3.3.1.1^{3,7}]decyl)-4-fluorophenyl)thiazol-4-yl}ethyl}piperidine-1-ylcarboxamide (**1j**)

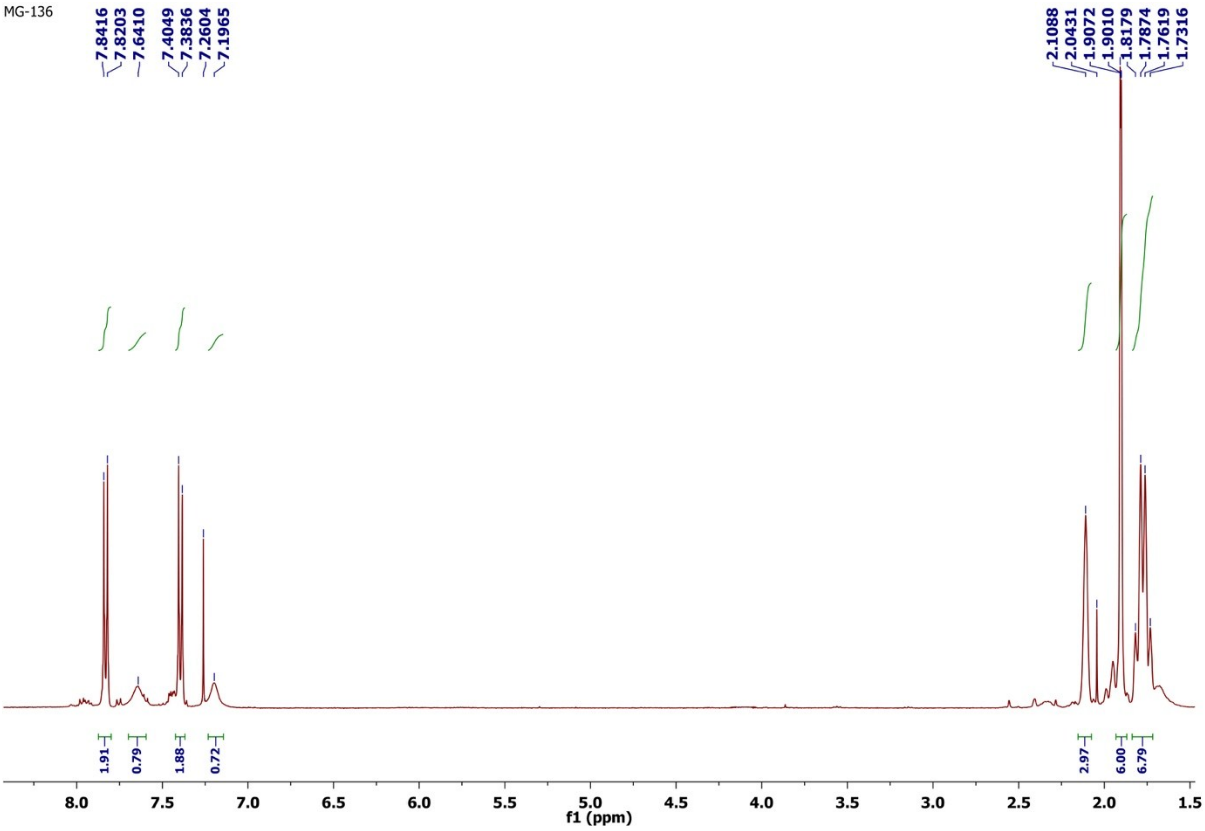

^{1}H NMR (600 MHz, $CDCl_3$) of 4-(1-tricyclo[3.3.1.1^{3,7}]decyl)thiobenzamide (**14**)

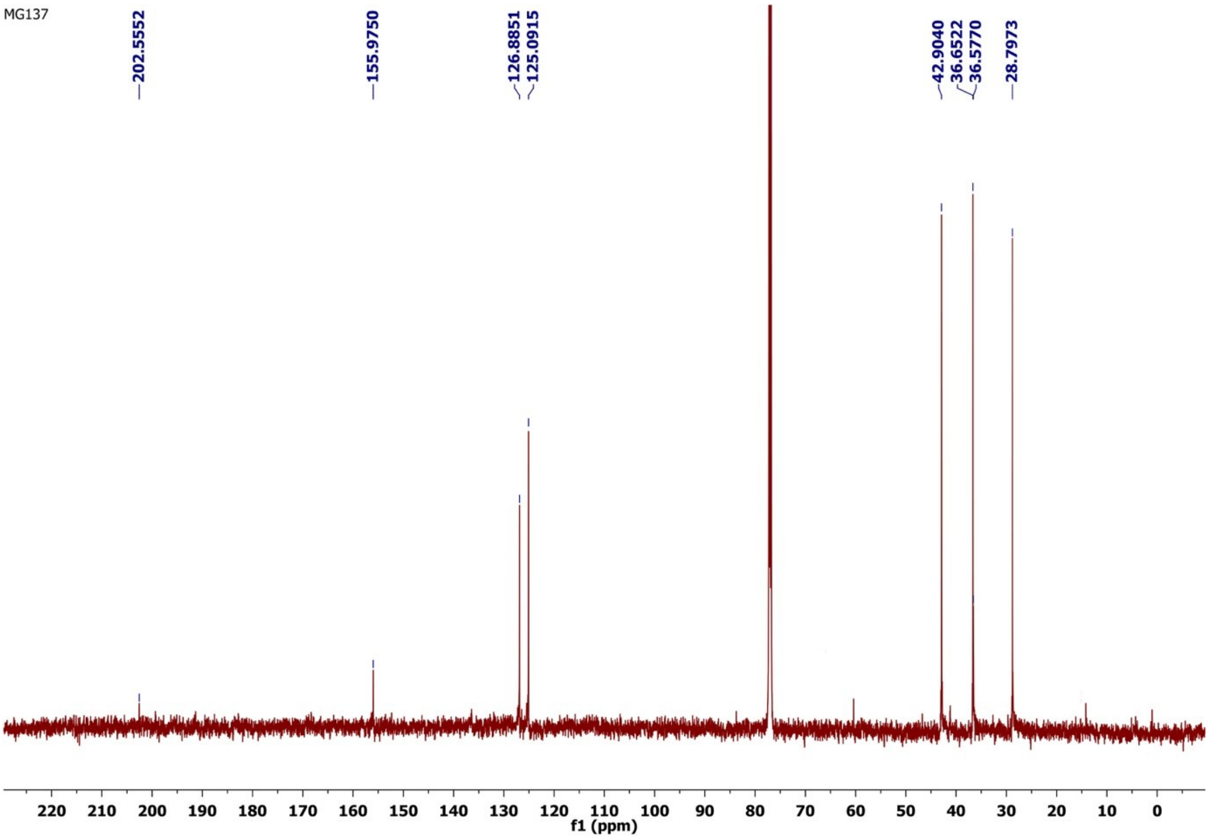

^{13}C NMR (150 MHz, $CDCl_3$) of 4-(1-tricyclo[3.3.1.1^{3,7}]decyl)thiobenzamide (**14**)

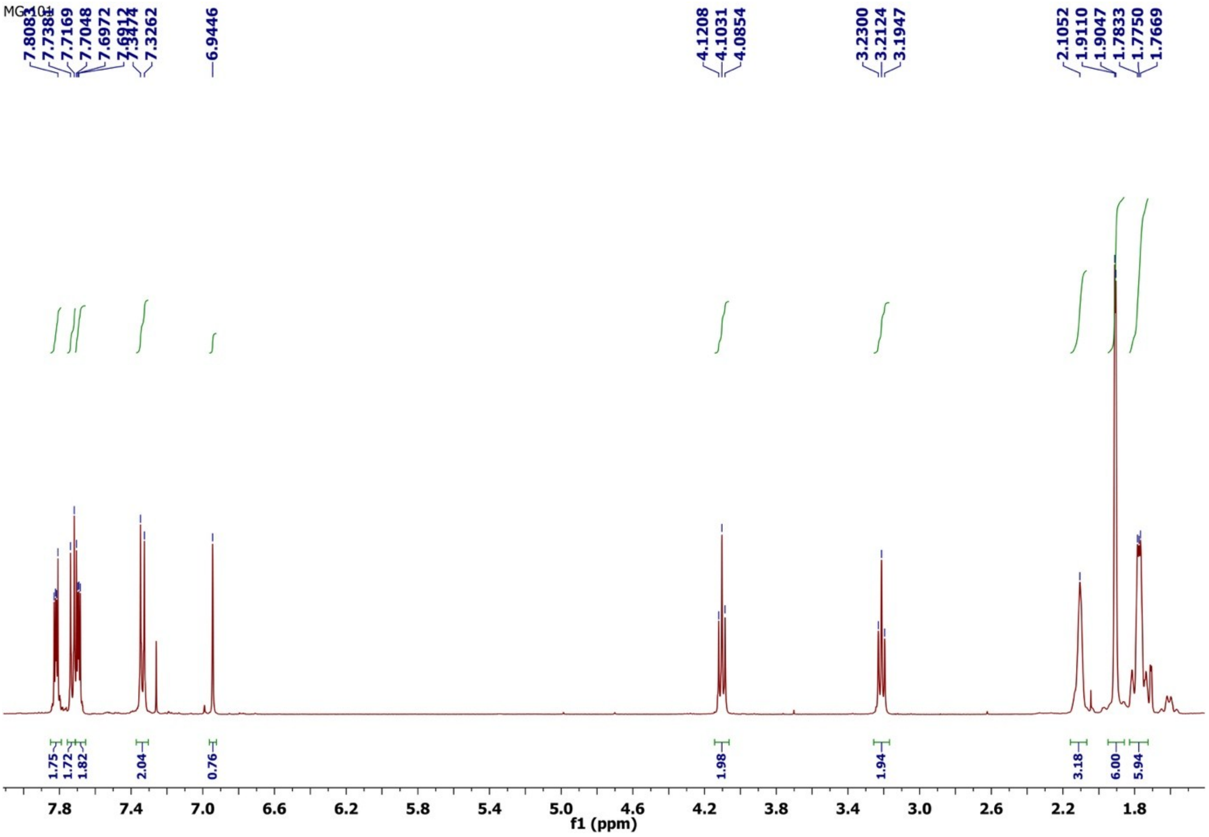

^{1}H NMR (600 MHz, $CDCl_3$) of 2-{2-[2-(4-(1-tricyclo[3.3.1.1^{3,7}]decyl)phenyl)thiazol-4-yl]ethyl}isoindoline-1,3-dione **(10)**

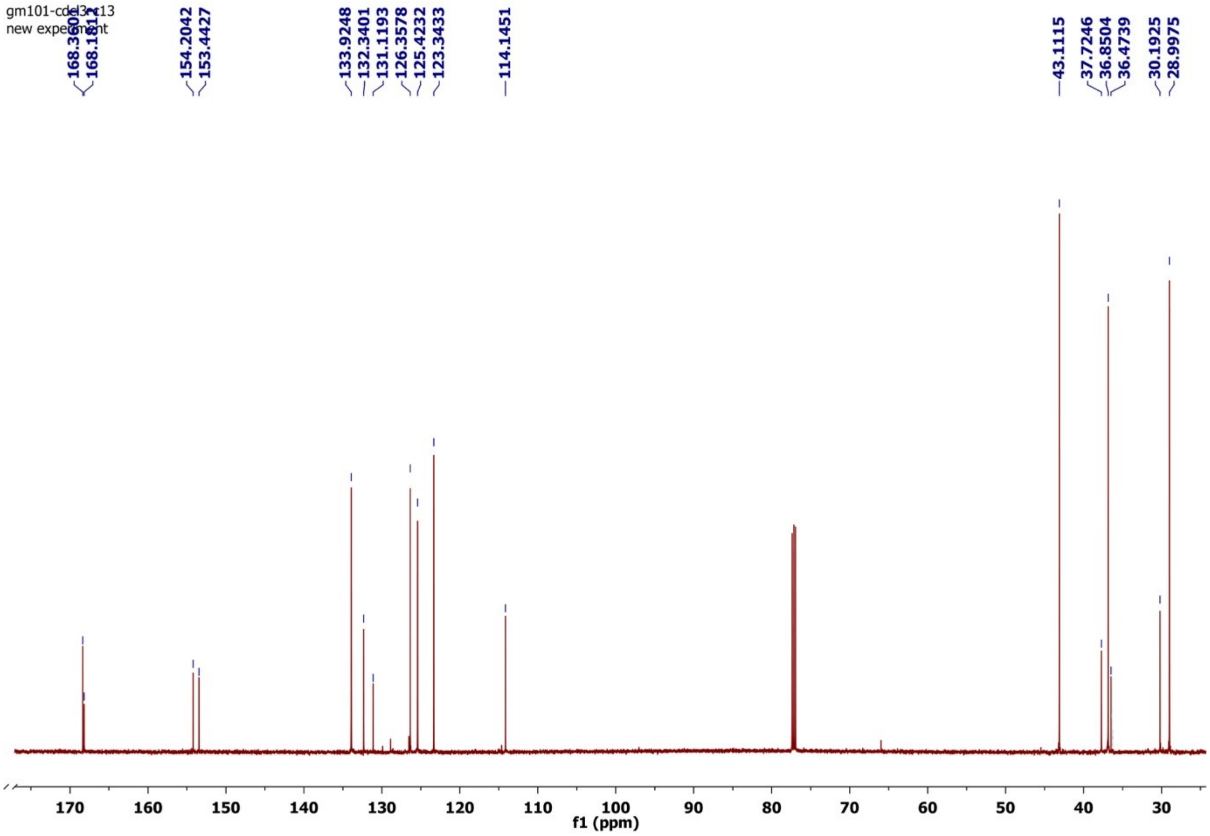

^{13}C NMR (150 MHz, $CDCl_3$) of 2-{2-[2-(4-(1-tricyclo[3.3.1.1^{3,7}]decyl)phenyl)thiazol-4-yl]ethyl}isoindoline-1,3-dione **(10)**

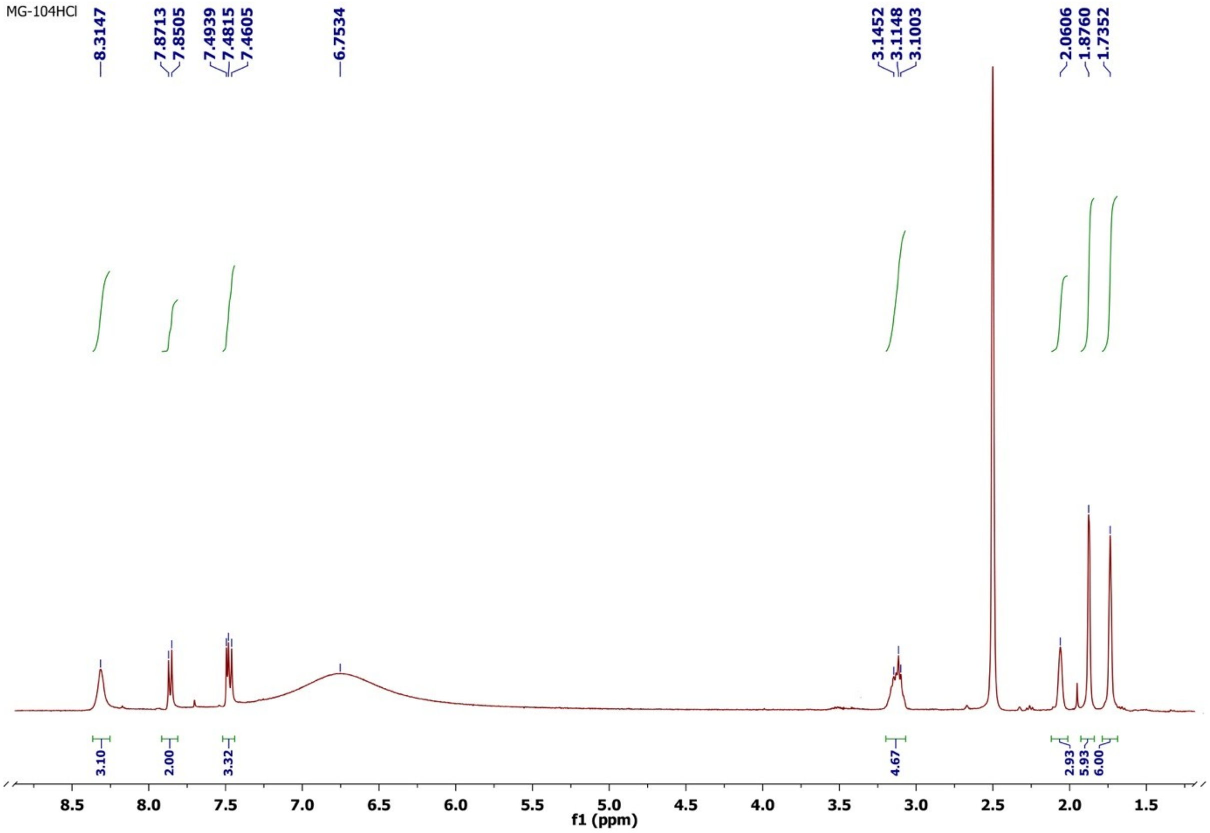

^{1}H NMR (400 MHz, DMSO-d_6) of 2-{2-[4-(1-tricyclo[3.3.1.1^{3,7}]decyl)phenyl]thiazol-4-yl}ethan-1-amine dihydrochloride (**2a**)

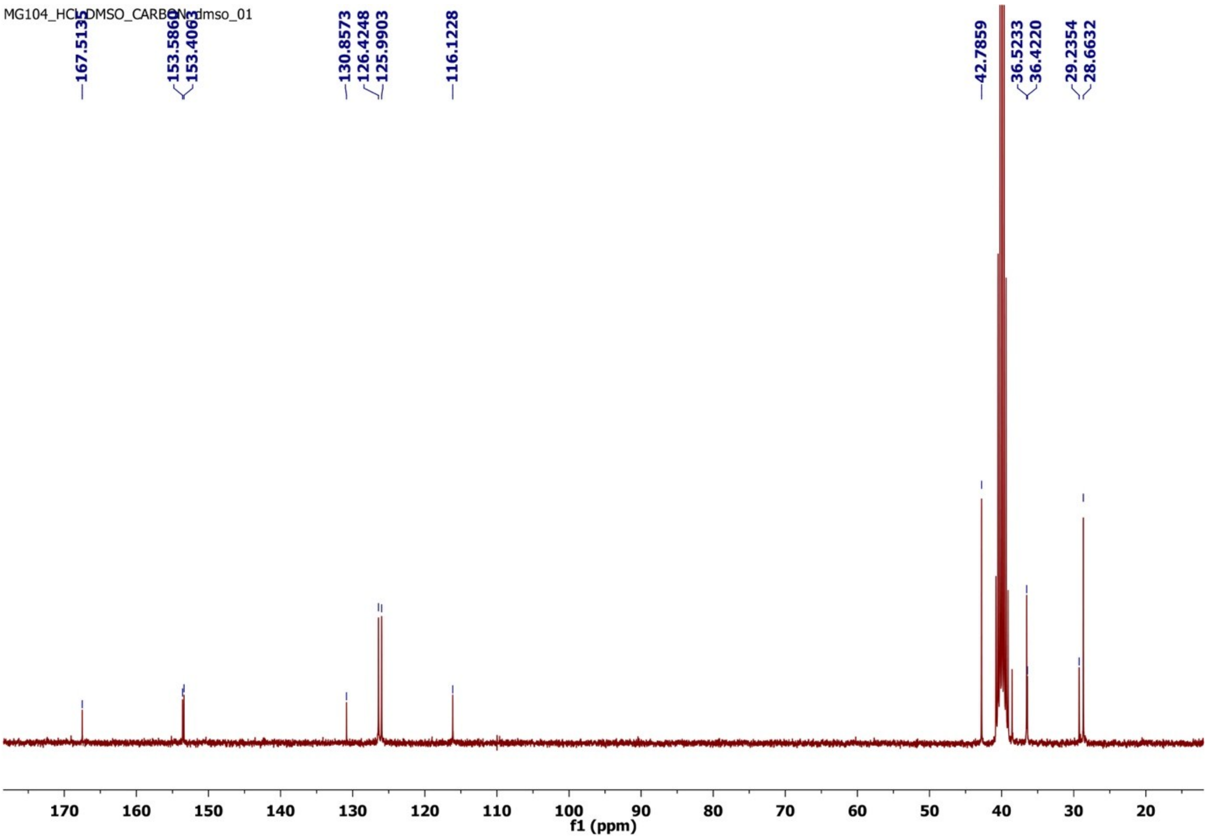

^{13}C NMR (75 MHz, DMSO-d_6) of 2-{2-[4-(1-tricyclo[3.3.1.1^{3,7}]decyl)phenyl]thiazol-4-yl}ethan-1-amine dihydrochloride (**2a**)

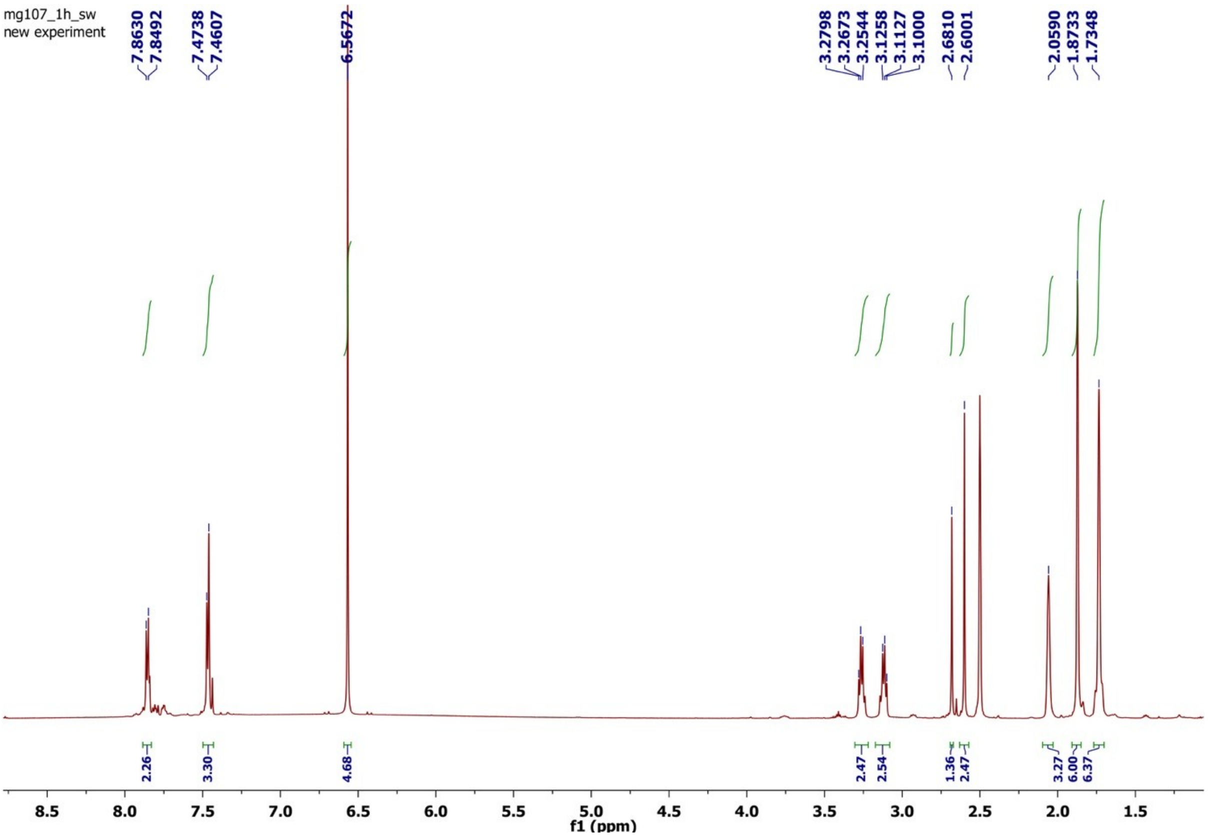

^{1}H NMR (600 MHz, DMSO-d_6) of 2-{2-[4-(1-tricyclo[3.3.1.1^{3,7}]decyl)phenyl]thiazol-4-yl}-*N*-methylethan-1-amine difumarate (**2b**)

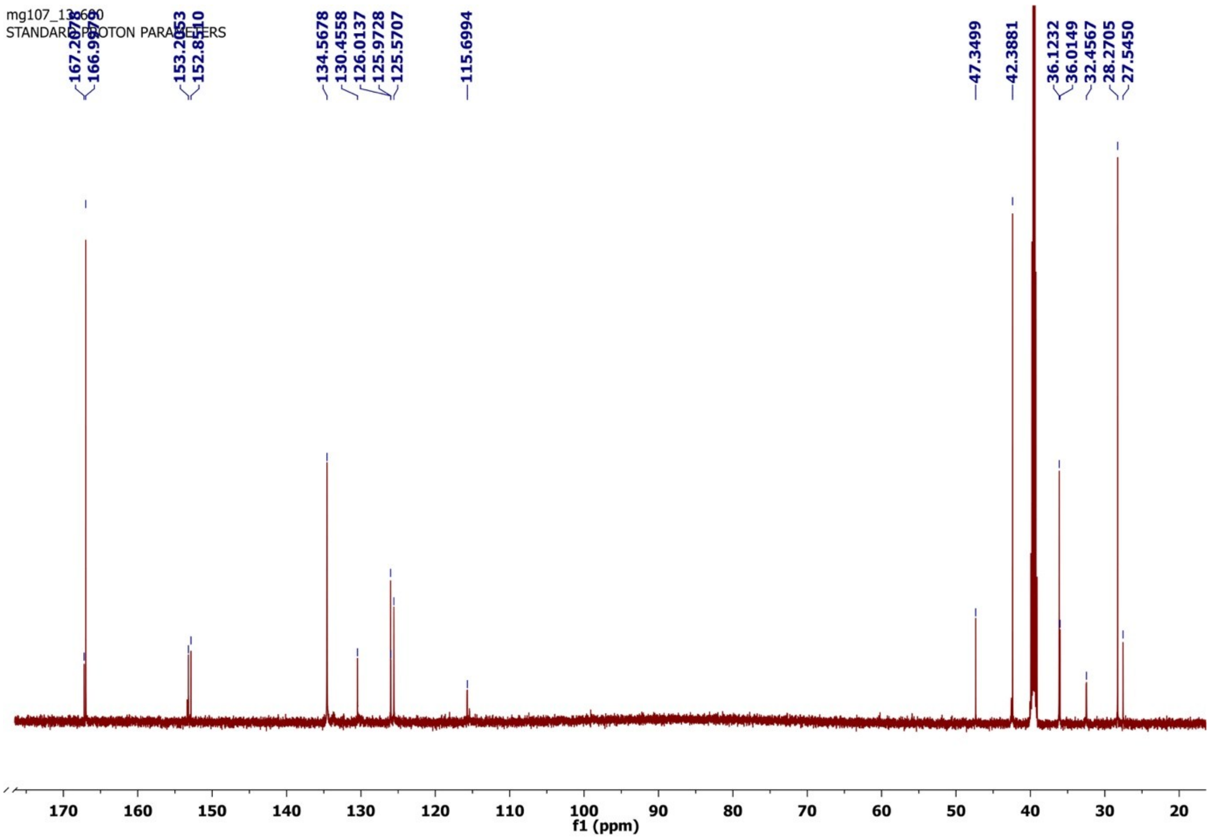

^{13}C NMR (150 MHz, DMSO-d_6) of 2-{2-[4-(1-tricyclo[3.3.1.1^{3,7}]decyl)phenyl]thiazol-4-yl}-*N*-methylethan-1-amine difumarate (**2b**)

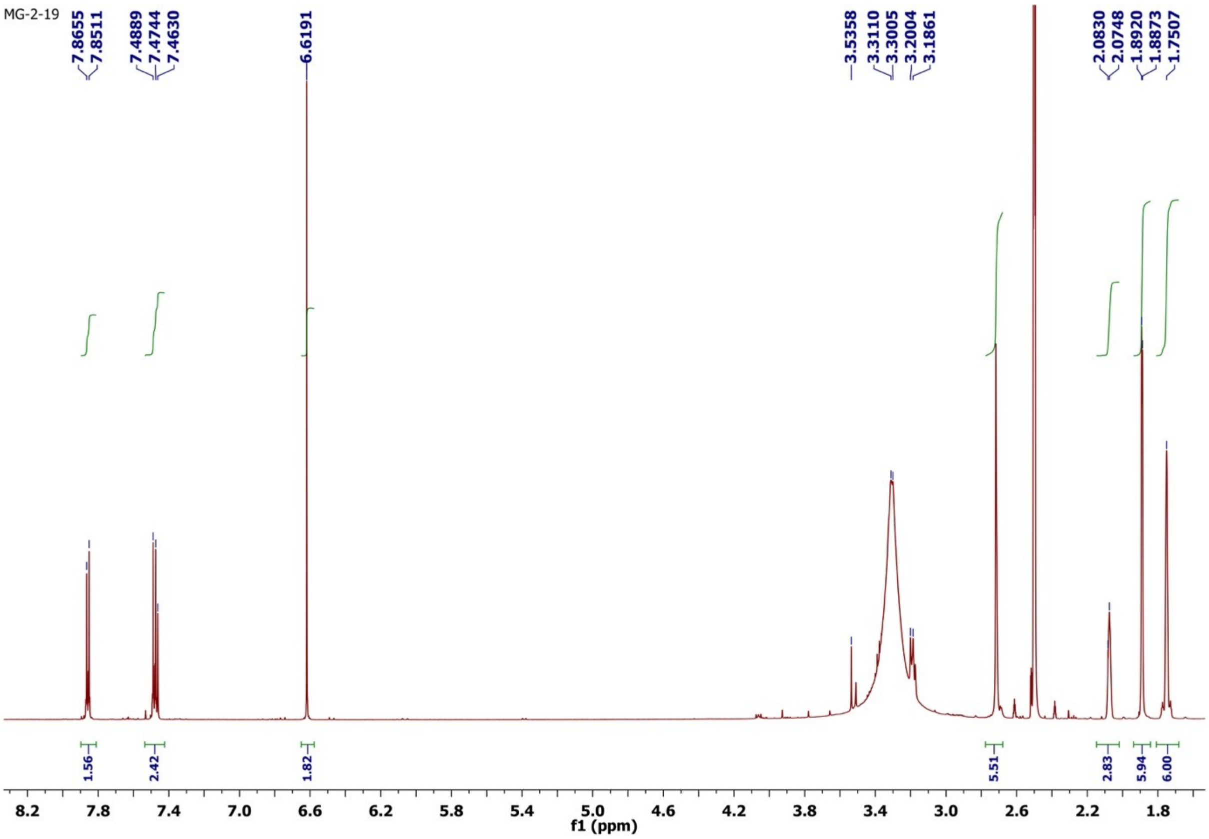

^{1}H NMR (400 MHz, DMSO-d_6) of 2-{2-[4-(1-tricyclo[3.3.1.1^{3,7}]decyl)phenyl]thiazol-4-yl}-*N,N*-dimethylethan-1-amine difumarate (**2c**)

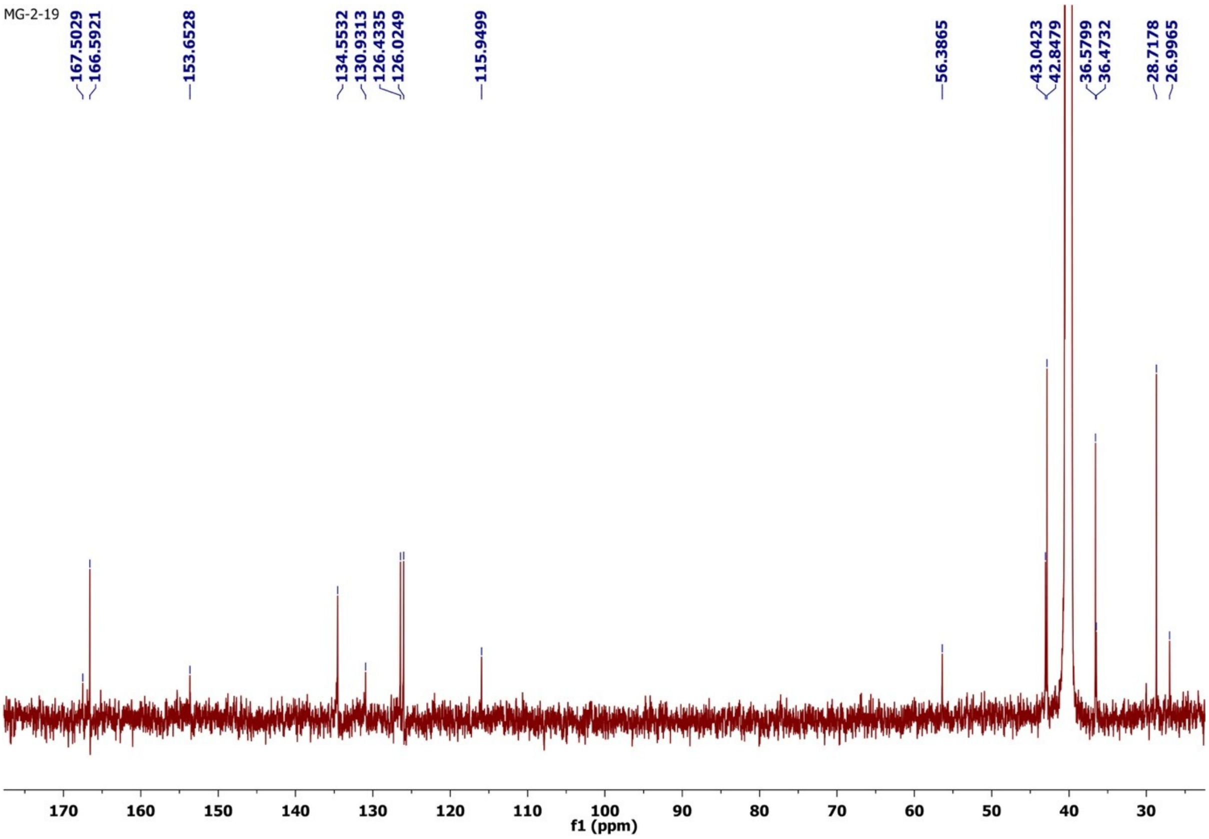

^{13}C NMR (150 MHz, DMSO-d_6) of 2-{2-[4-(1-tricyclo[3.3.1.1^{3,7}]decyl)phenyl]thiazol-4-yl}-*N,N*-dimethylethan-1-amine difumarate (**2c**)

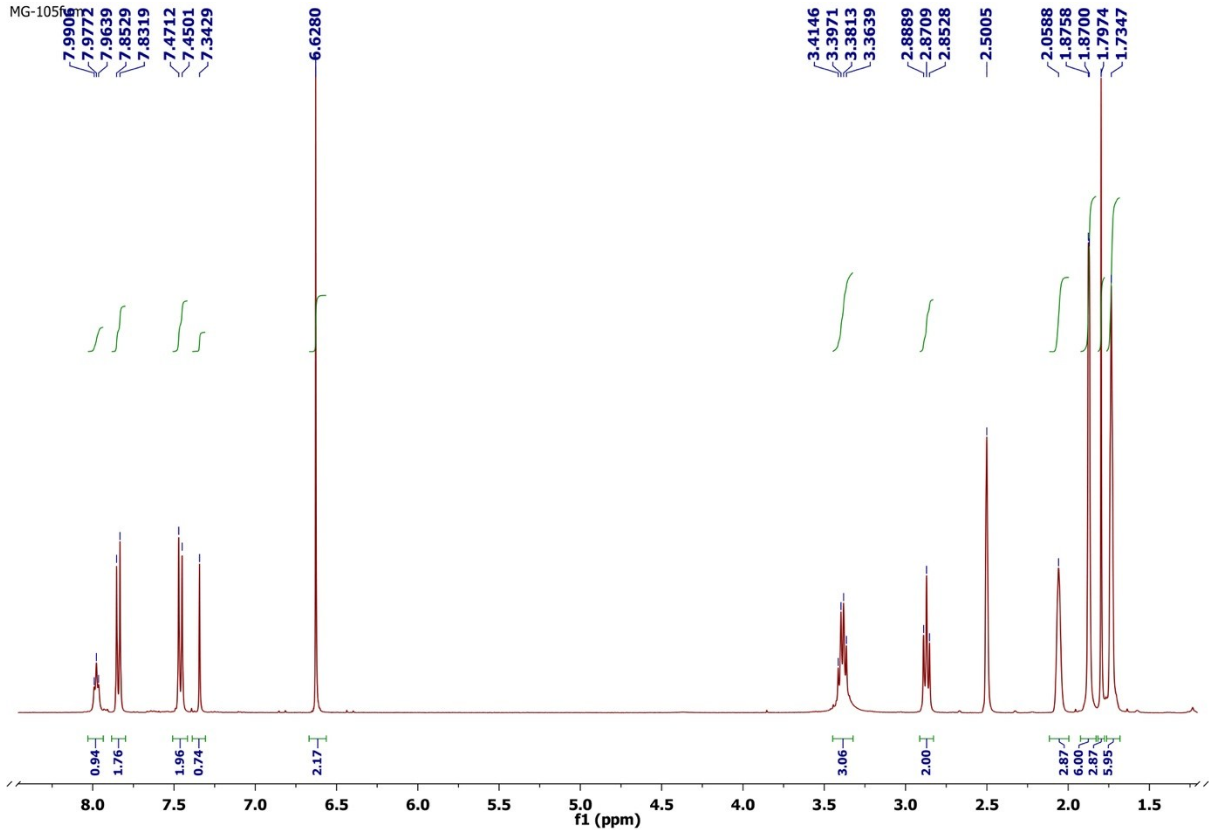

^{1}H NMR (600 MHz, DMSO-d_6) of *N*-{2-[2-(4-(1-tricyclo[3.3.1.1^{3,7}]decyl)phenyl)thiazol-4-yl]ethyl}acetamide fumarate **(2d)**

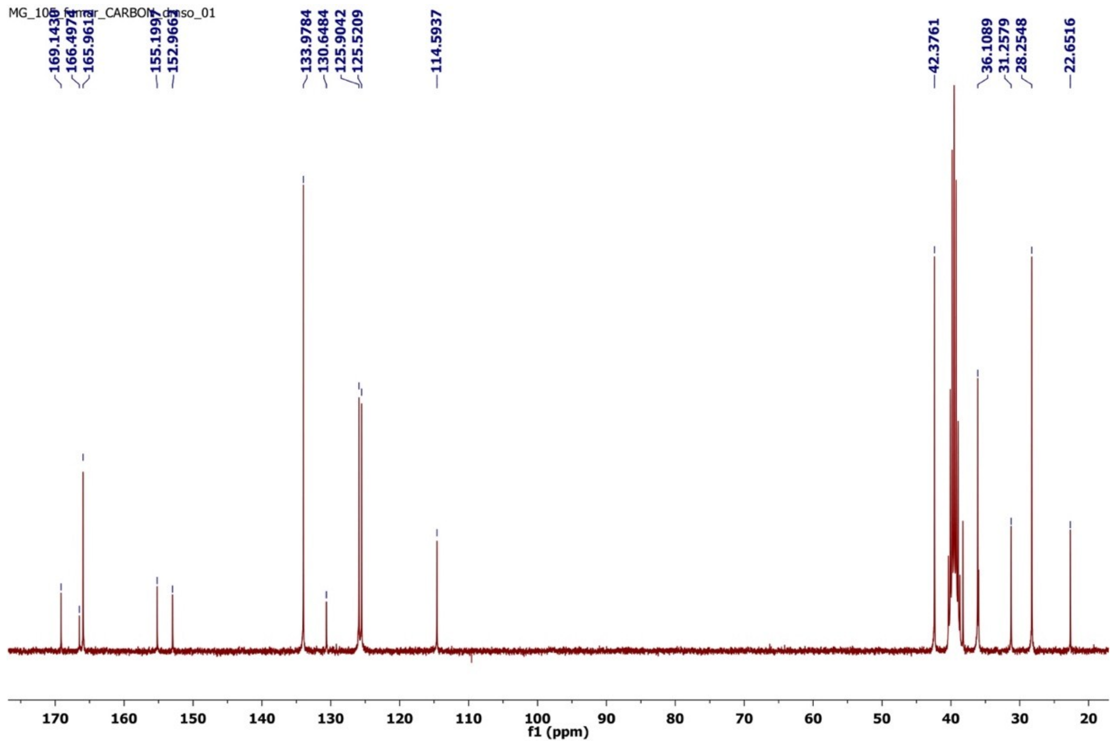

^{13}C NMR (75 MHz, DMSO-d_6) of *N*-{2-[2-(4-(1-tricyclo[3.3.1.1^{3,7}]decyl)phenyl)thiazol-4-yl]ethyl}acetamide fumarate **(2d)**

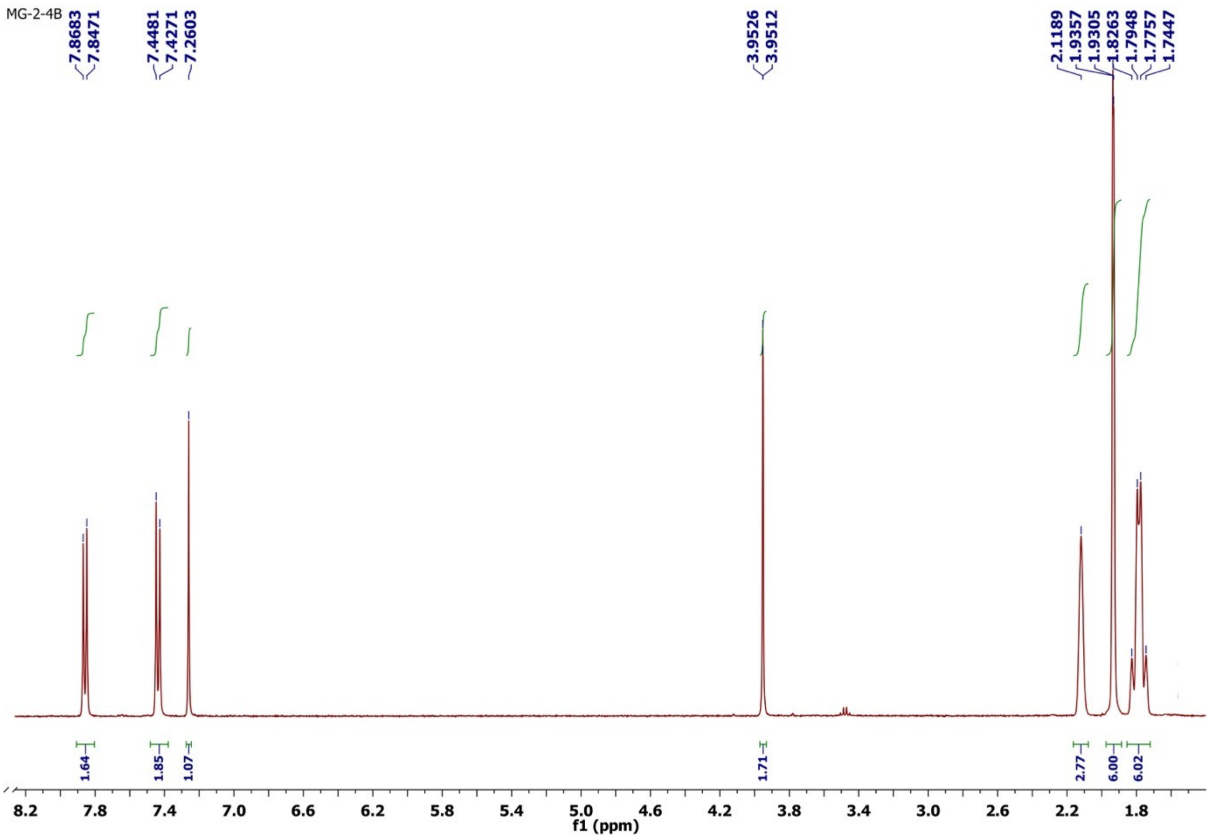

^{1}H NMR (400 MHz, $CDCl_3$) of 2-{2-[4-(1-tricyclo[3.3.1.1^{3,7}]decyl)phenyl]thiazol-4-yl}acetonitrile (**2e**)

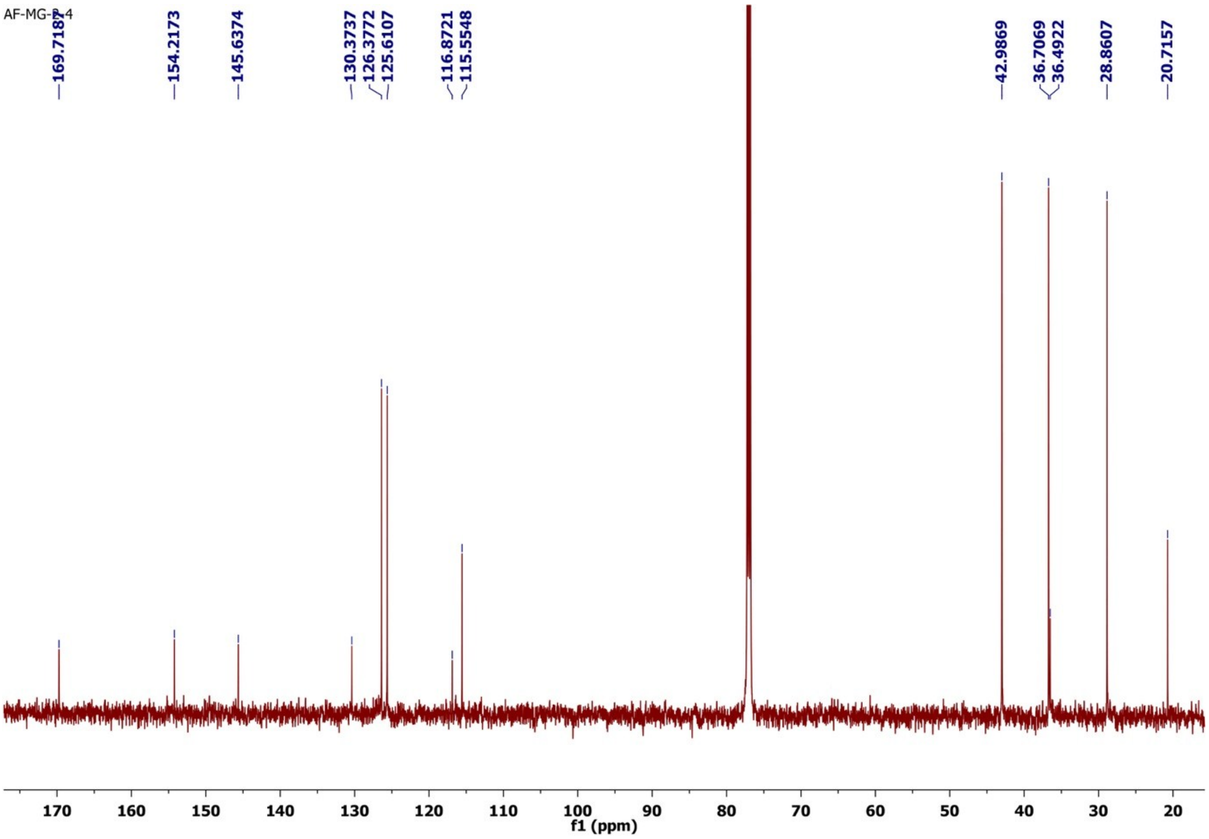

^{13}C NMR (150 Hz, $CDCl_3$) of 2-{2-[4-(1-tricyclo[3.3.1.1^{3,7}]decyl)phenyl]thiazol-4-yl}acetonitrile (**2e**)

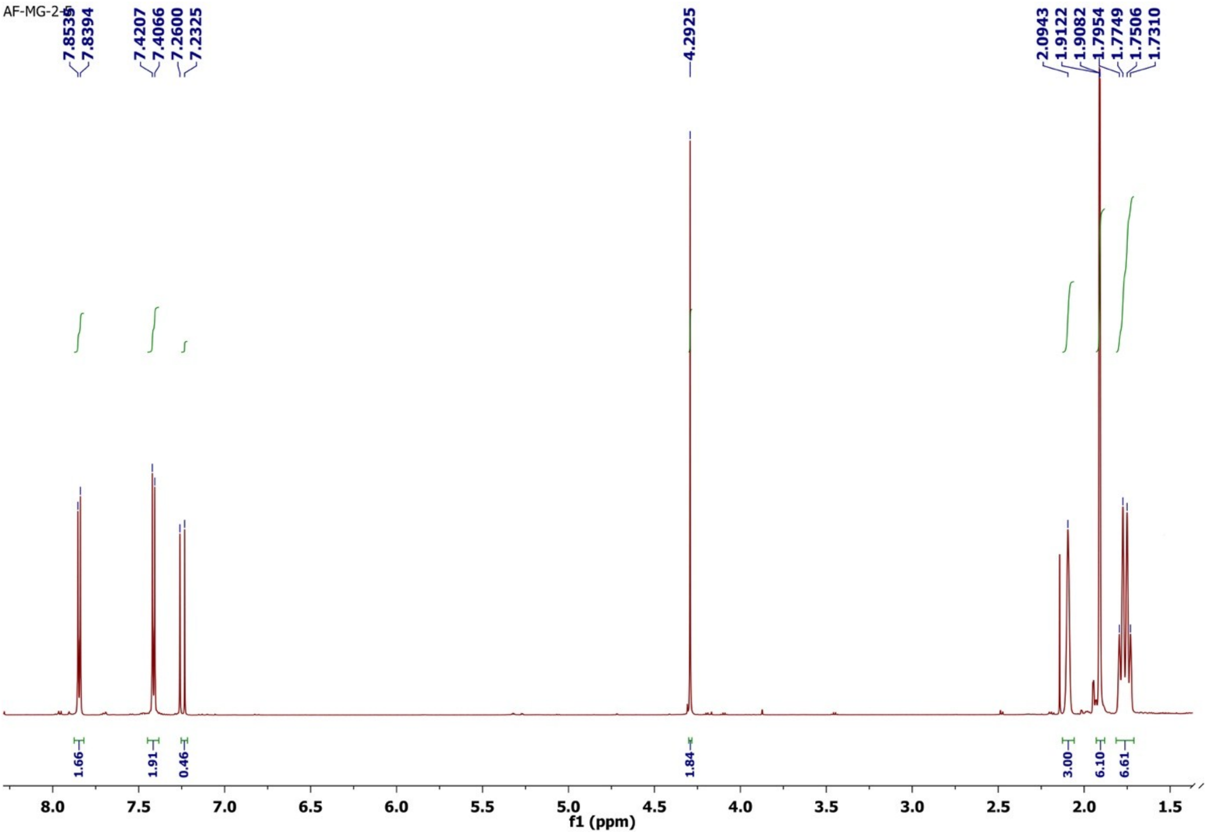

^{1}H NMR (600 MHz, $CDCl_3$) of 2-[4-(1-tricyclo[3.3.1.1^{3,7}]decyl)phenyl]-4-(thiocyanatemethyl)thiazole (**2f**)

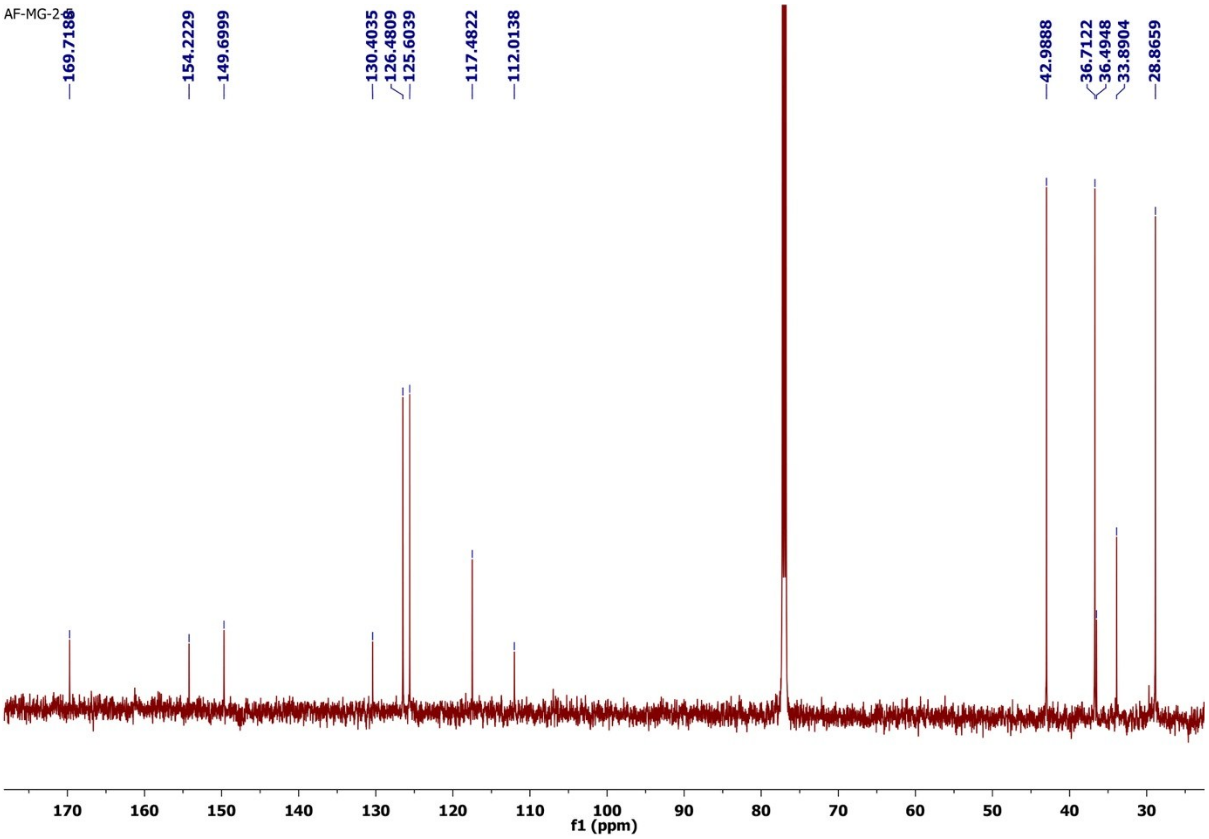

^{13}C NMR (150 Hz, $CDCl_3$) of 2-[4-(1-tricyclo[3.3.1.1^{3,7}]decyl)phenyl]-4-(thiocyanatemethyl)thiazole (**2f**)

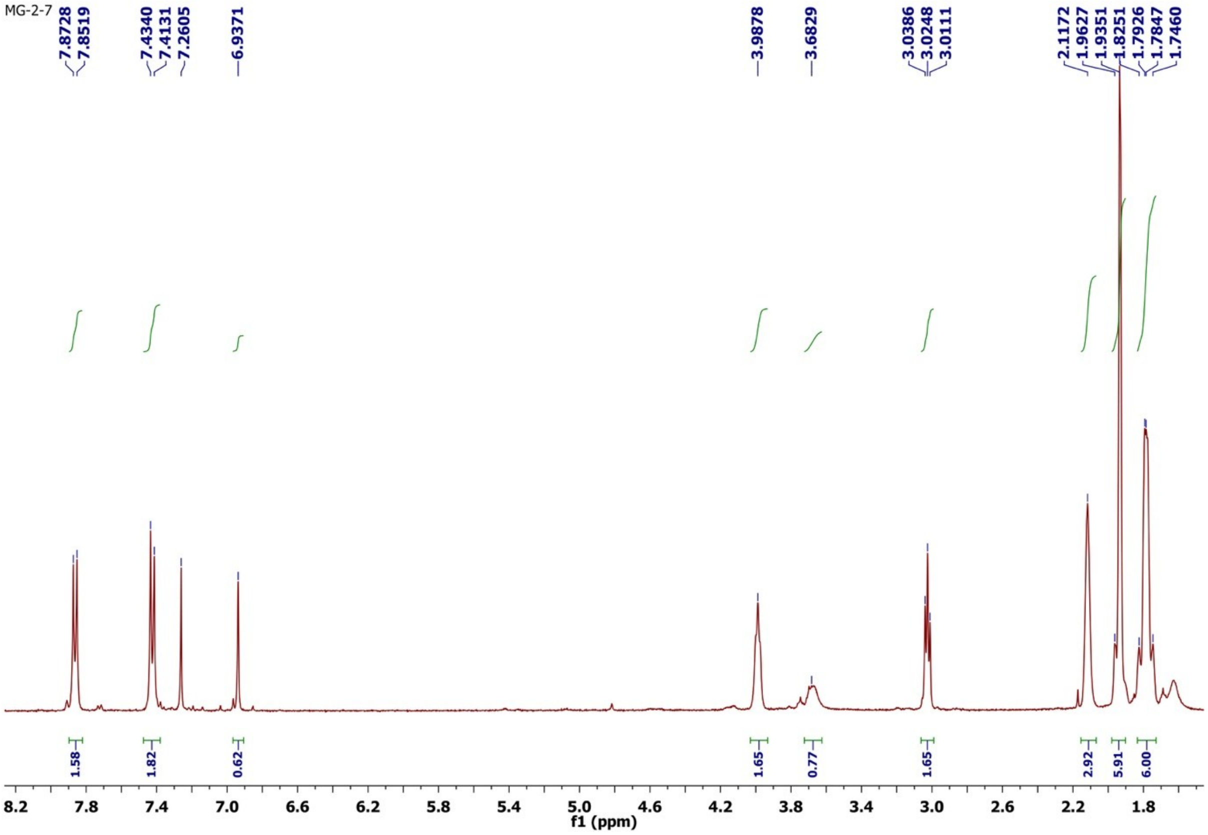

^{1}H NMR (400 MHz, $CDCl_3$) of 2-{2-[4-(1-tricyclo[3.3.1.1^{3,7}]decyl)phenyl]thiazol-4-yl}ethan-1-ol (**2g**)

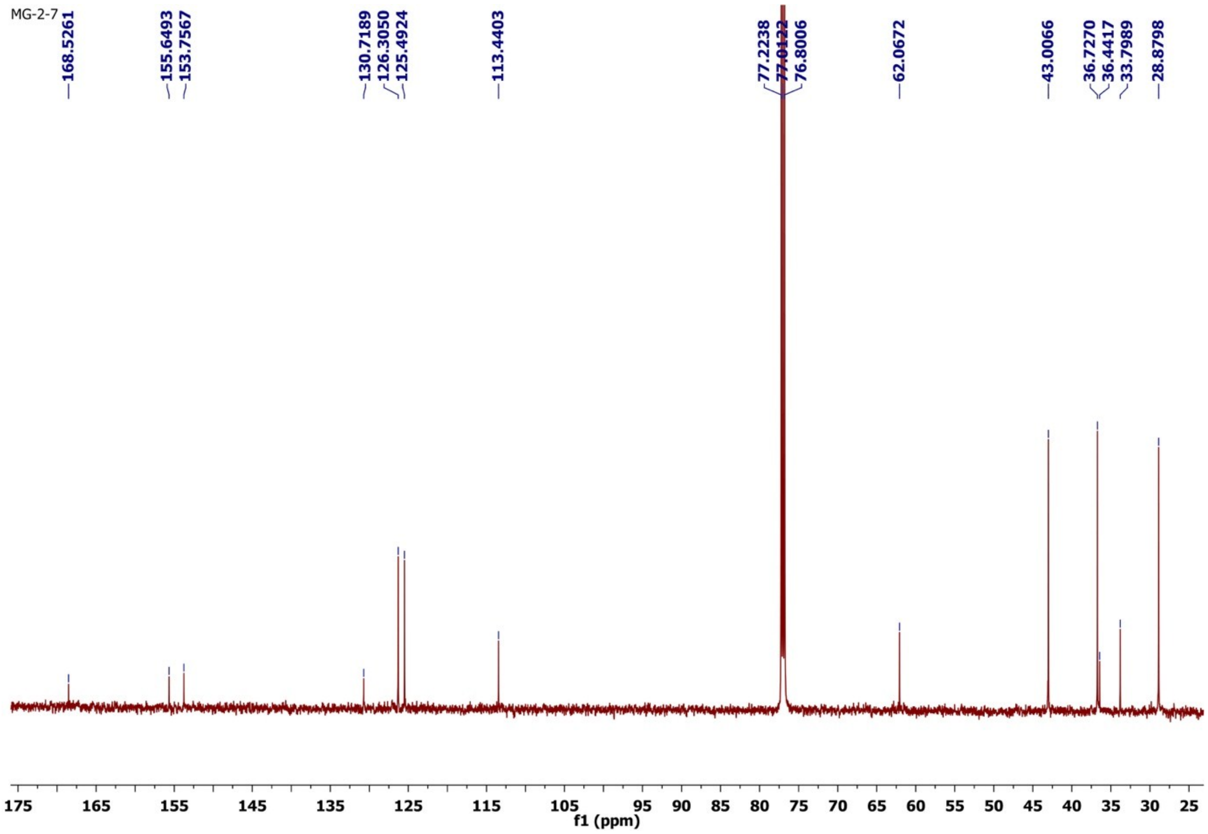

^{13}C NMR (150 MHz, $CDCl_3$) of 2-{2-[4-(1-tricyclo[3.3.1.1^{3,7}]decyl)phenyl]thiazol-4-yl}ethan-1-ol (**2g**)

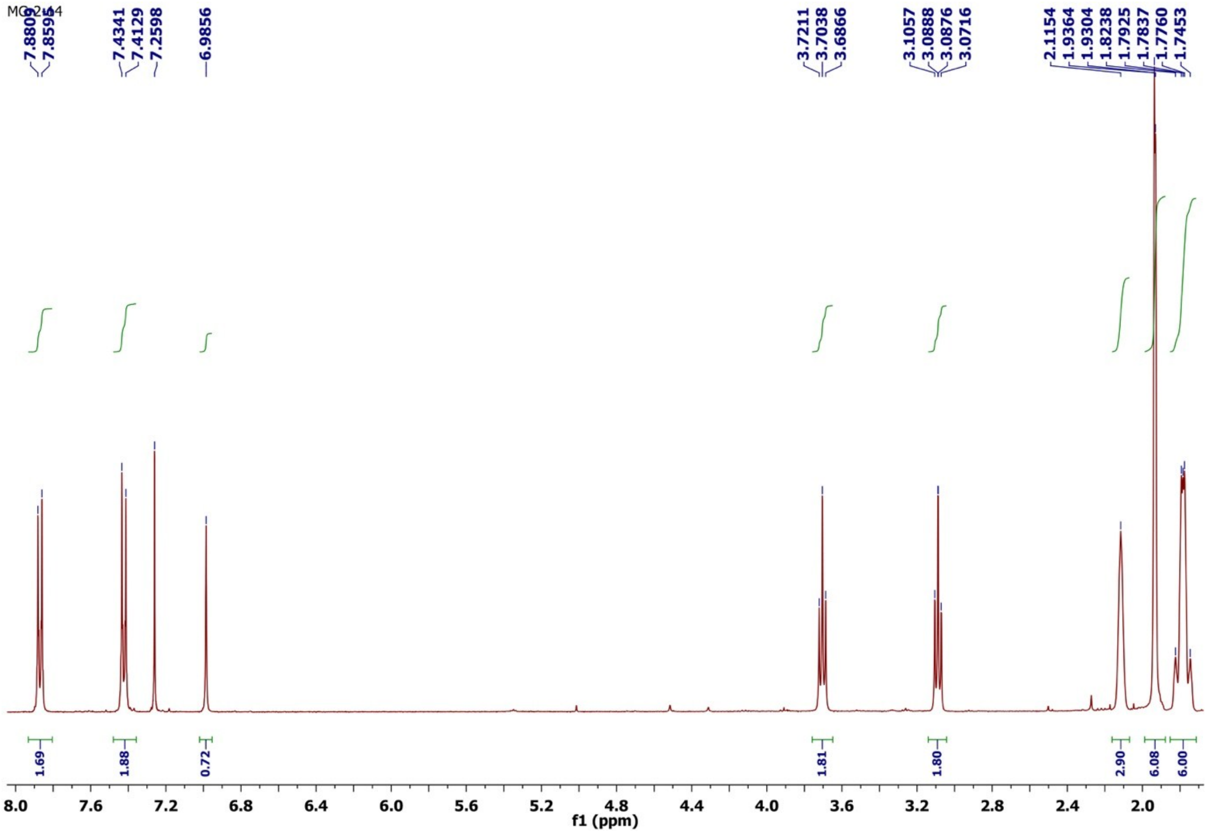

^{1}H NMR (400 MHz, $CDCl_3$) of 2-[4-(1-tricyclo[3.3.1.1^{3,7}]decyl)phenyl]-4-(2-azidoethyl)thiazole (**2h**)

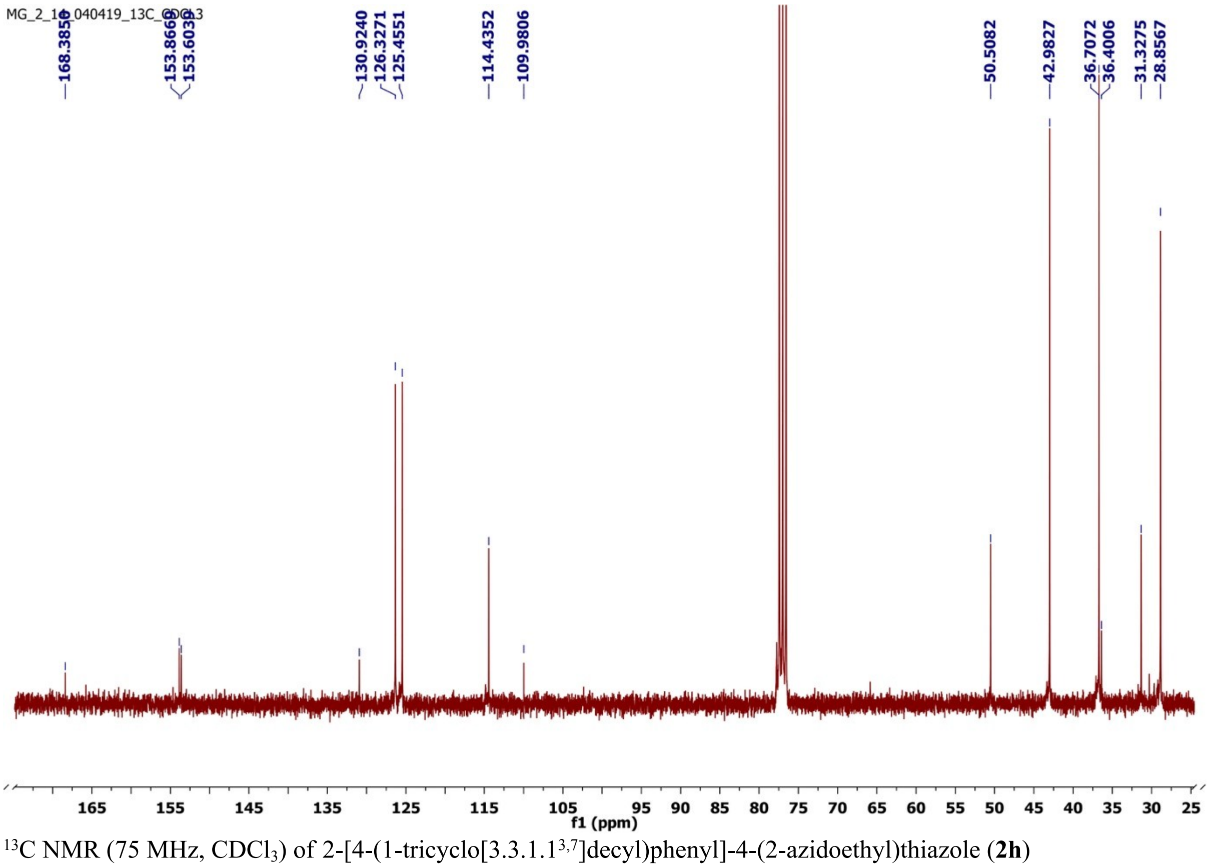

^{13}C NMR (75 MHz, $CDCl_3$) of 2-[4-(1-tricyclo[3.3.1.1^{3,7}]decyl)phenyl]-4-(2-azidoethyl)thiazole (**2h**)

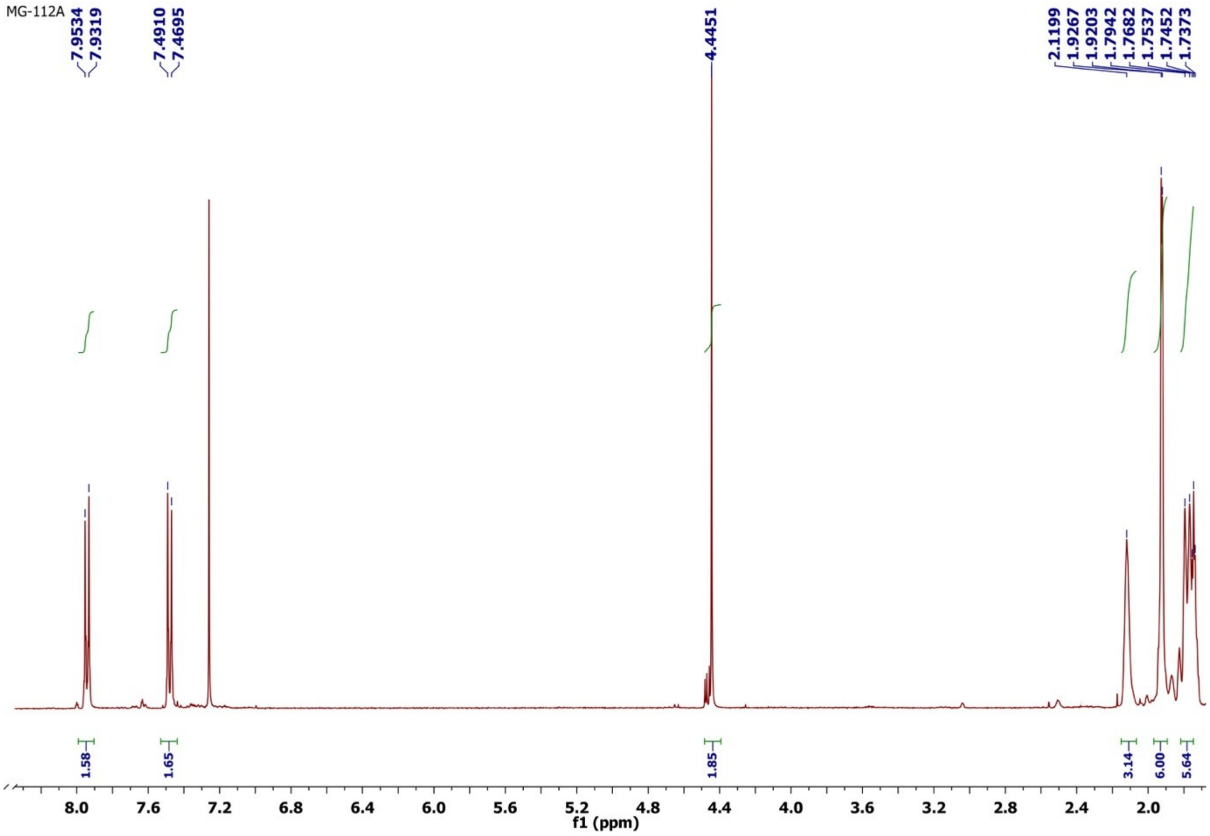

^{1}H NMR (400 MHz, $CDCl_3$) of 2-bromo-1-[4-(1-tricyclo[3.3.1.1^{3,7}]decyl)phenyl]ethanone (**19**)

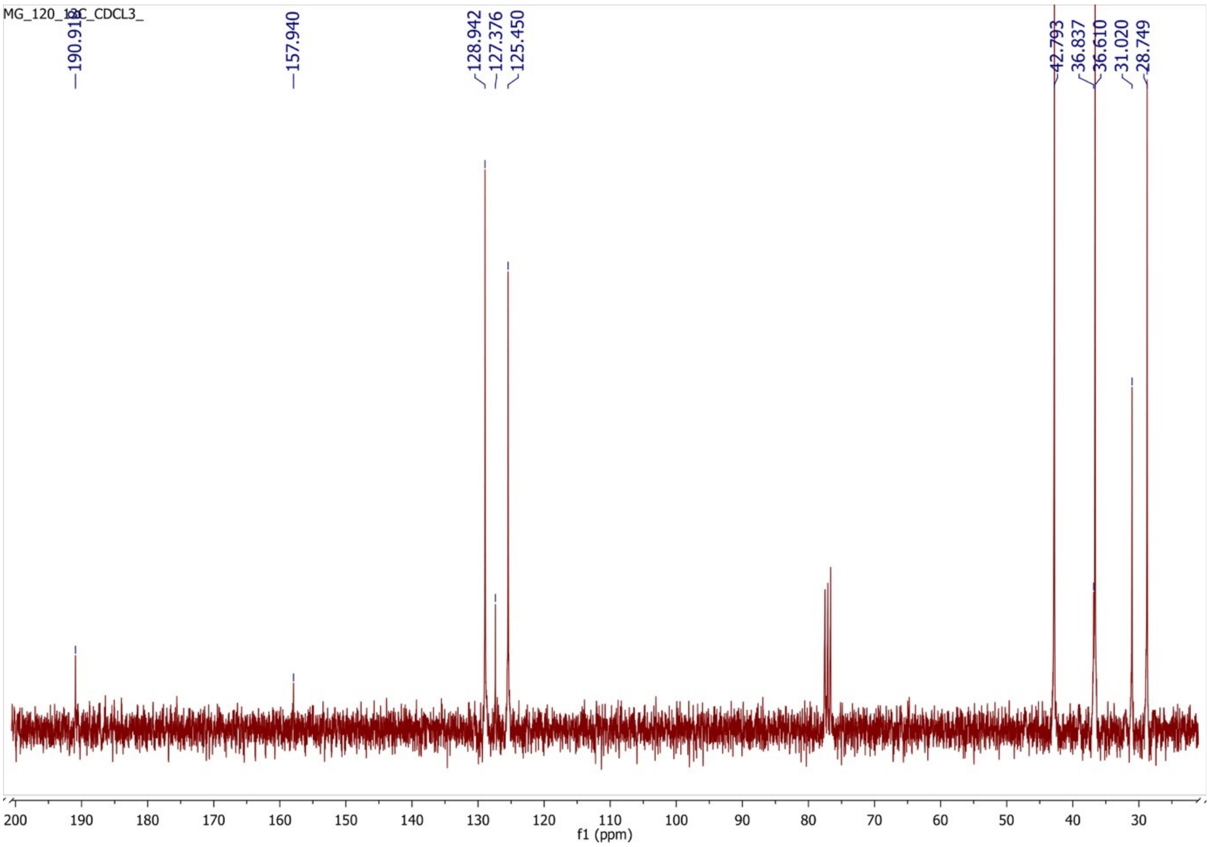

^{13}C NMR (75 MHz, $CDCl_3$) of 2-bromo-1-[4-(1-tricyclo[3.3.1.1^{3,7}]decyl)phenyl]ethanone (**19**)

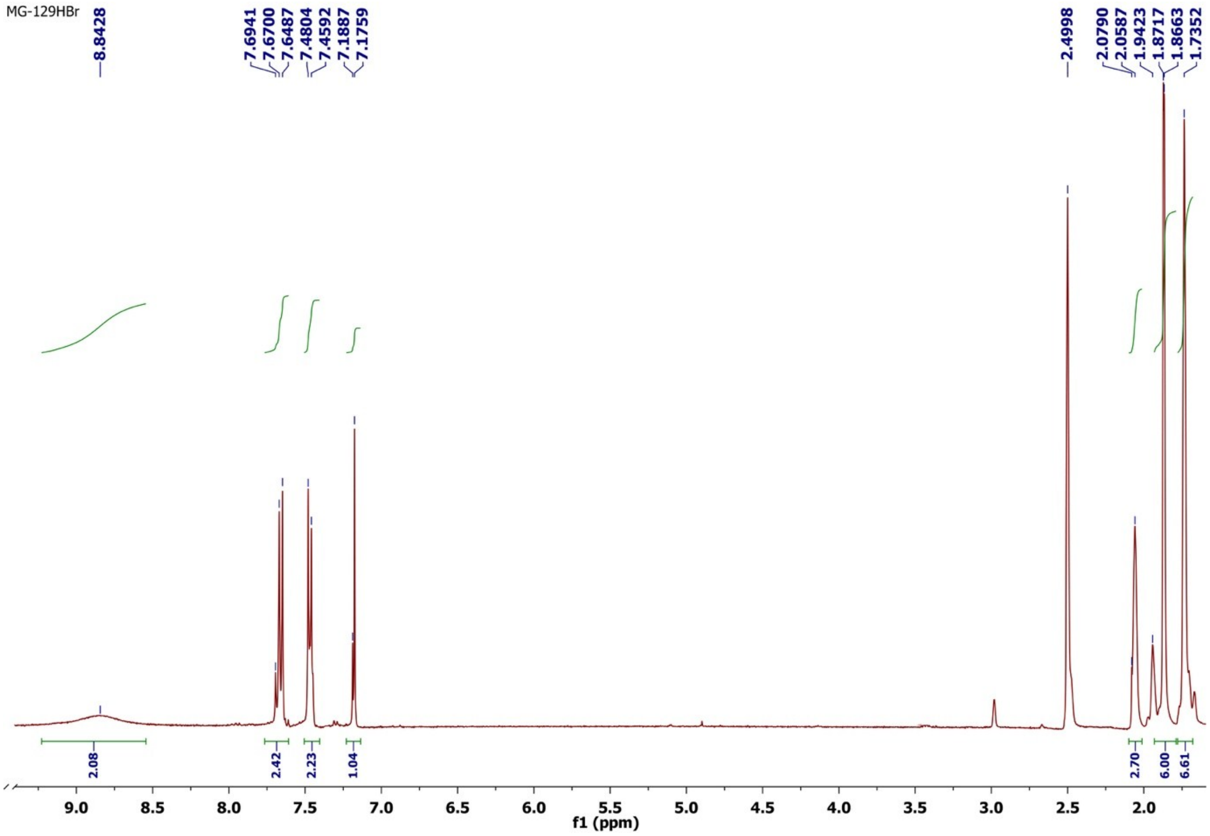

^{1}H NMR (400 MHz, DMSO-d_6) of 4-[4-(1-tricyclo[3.3.1.1^{3,7}]decyl)phenyl]thiazol-2-amine hydrobromide (**3a**)

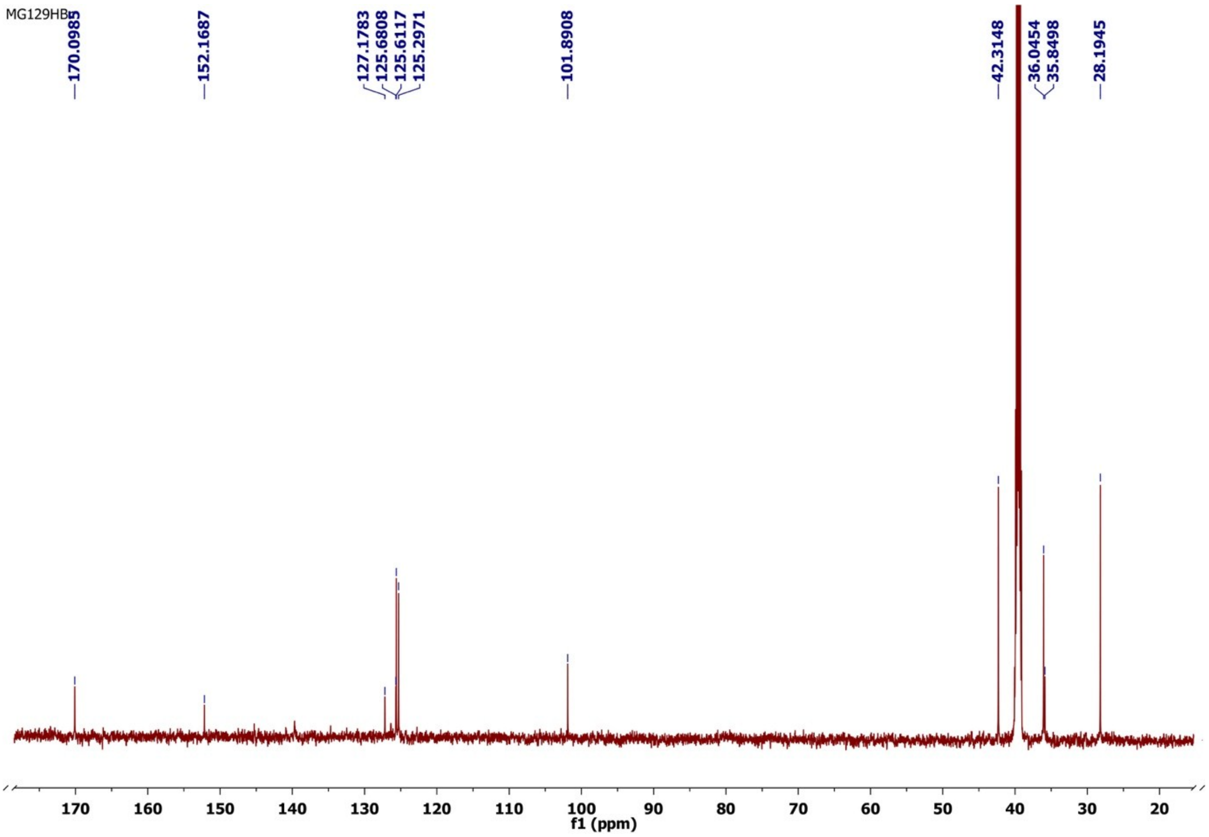

^{13}C NMR (150 MHz, DMSO-d_6) of 4-[4-(1-tricyclo[3.3.1.1^{3,7}]decyl)phenyl]thiazol-2-amine hydrobromide (**3a**)

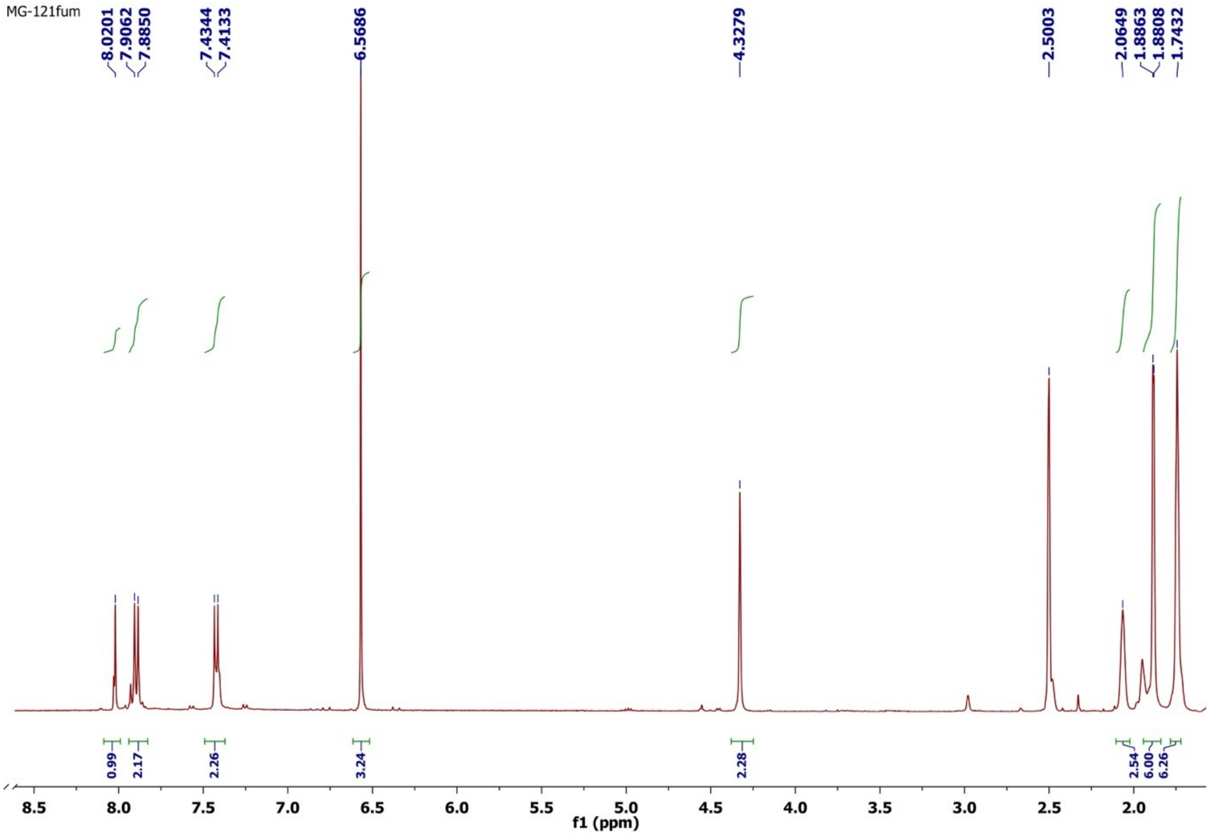

^{1}H NMR (400 MHz, DMSO-d_6) of {4-[4-(1-tricyclo[3.3.1.1^{3,7}]decyl)phenyl]thiazol-2-yl}methanamine difumarate **(3b)**

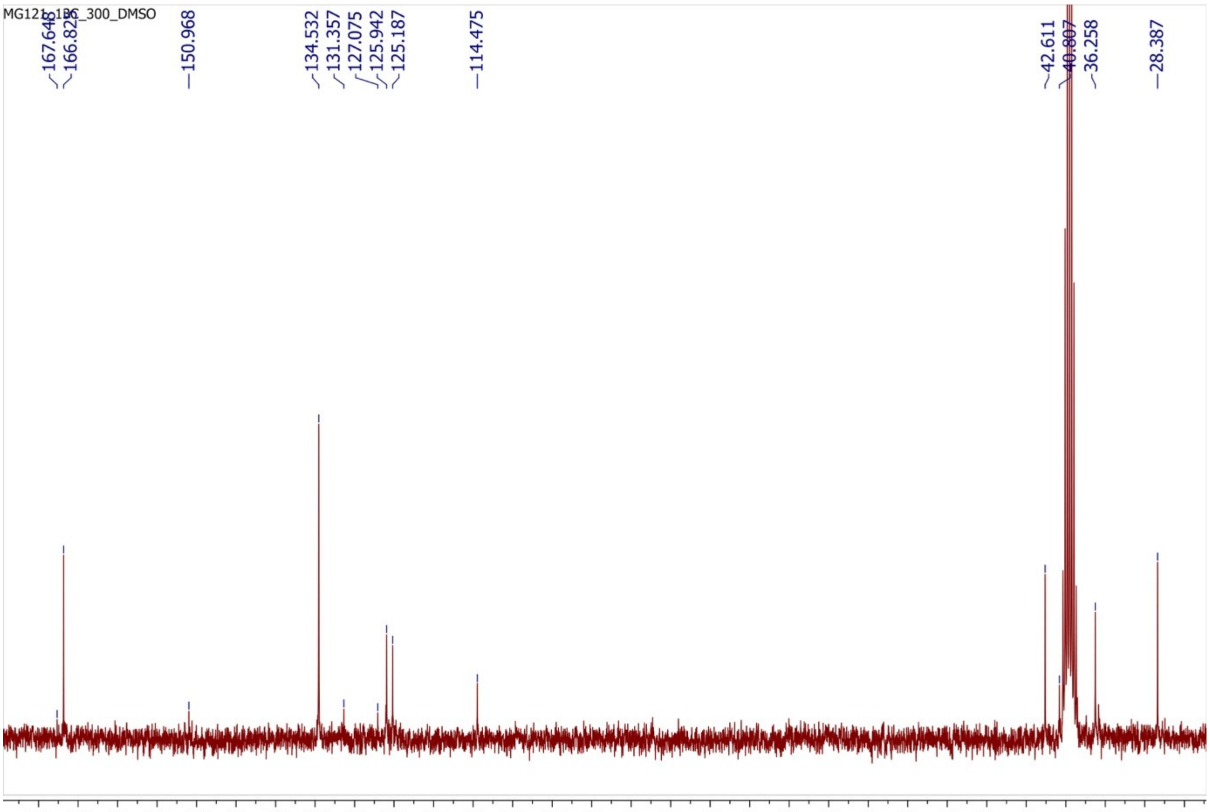

^{13}C NMR (75 MHz, DMSO-d_6) of {4-[4-(1-tricyclo[3.3.1.1^{3,7}]decyl)phenyl]thiazol-2-yl}methanamine difumarate **(3b)**

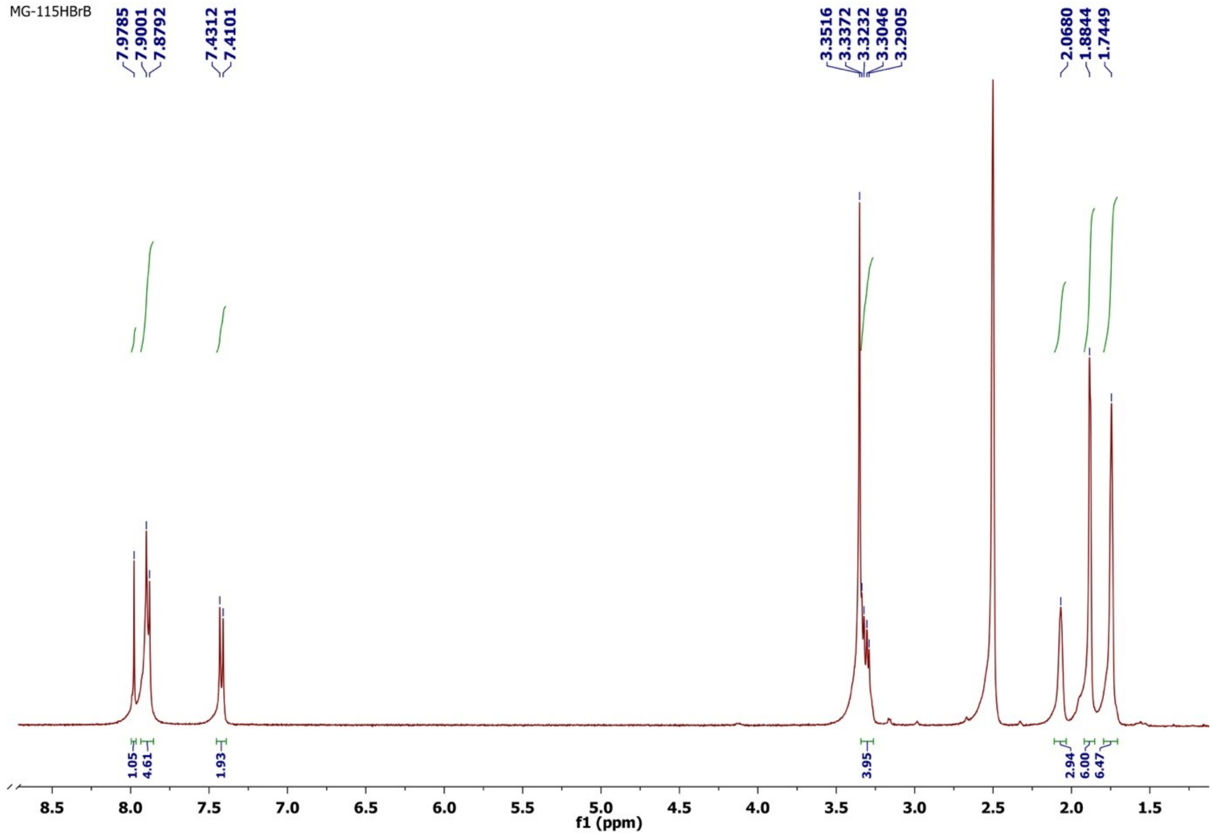

^{1}H NMR (400 MHz, DMSO-d_6) of 2-{4-[4-(1-tricyclo[3.3.1.1^{3,7}]decyl)phenyl]thiazol-2-yl}ethanamine hydrobromide (**3c**)

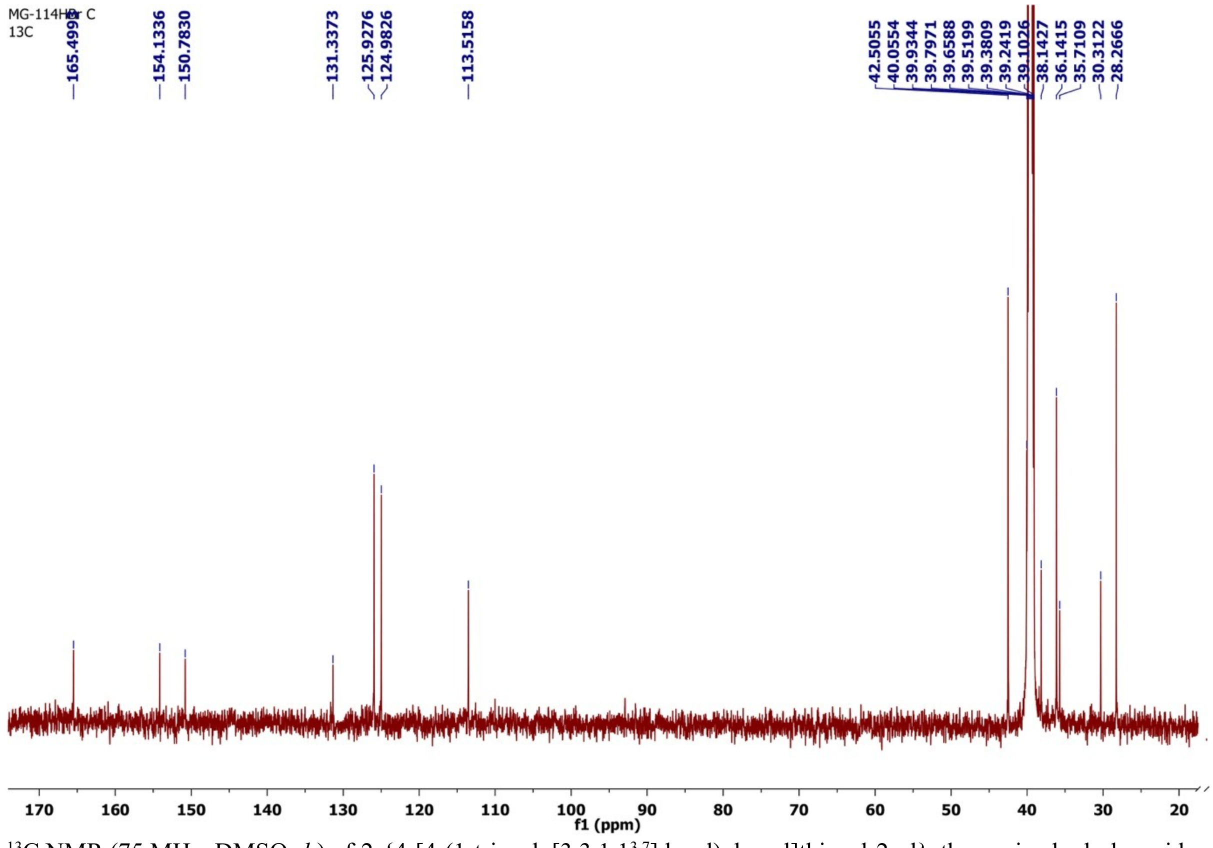

^{13}C NMR (75 MHz, DMSO-d_6) of 2-{4-[4-(1-tricyclo[3.3.1.1^{3,7}]decyl)phenyl]thiazol-2-yl}ethanamine hydrobromide (**3c**)

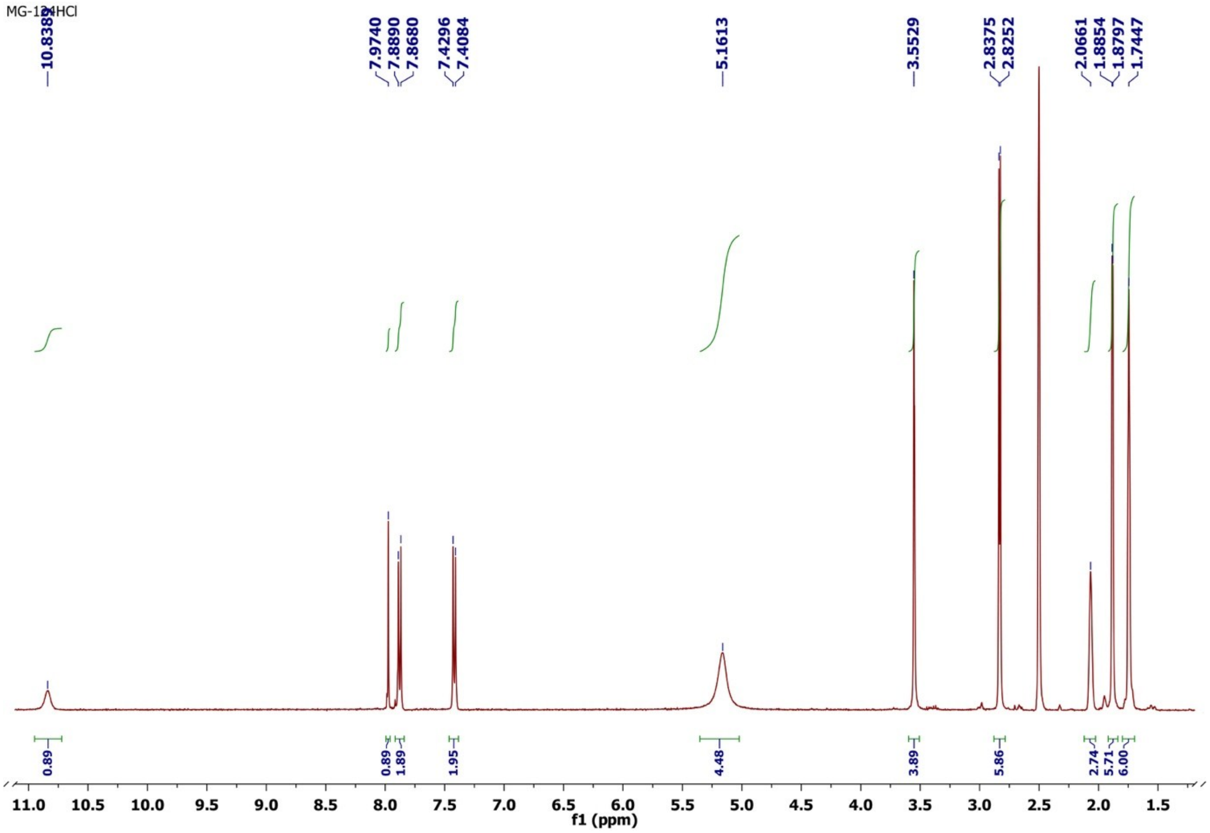

^{1}H NMR (400 MHz, DMSO-d_6) of 2-{4-[4-(1-tricyclo[3.3.1.1^{3,7}]decyl)phenyl]thiazol-2-yl}-*N,N*-dimethylethan-1-amine dihydrochloride (**3d**)

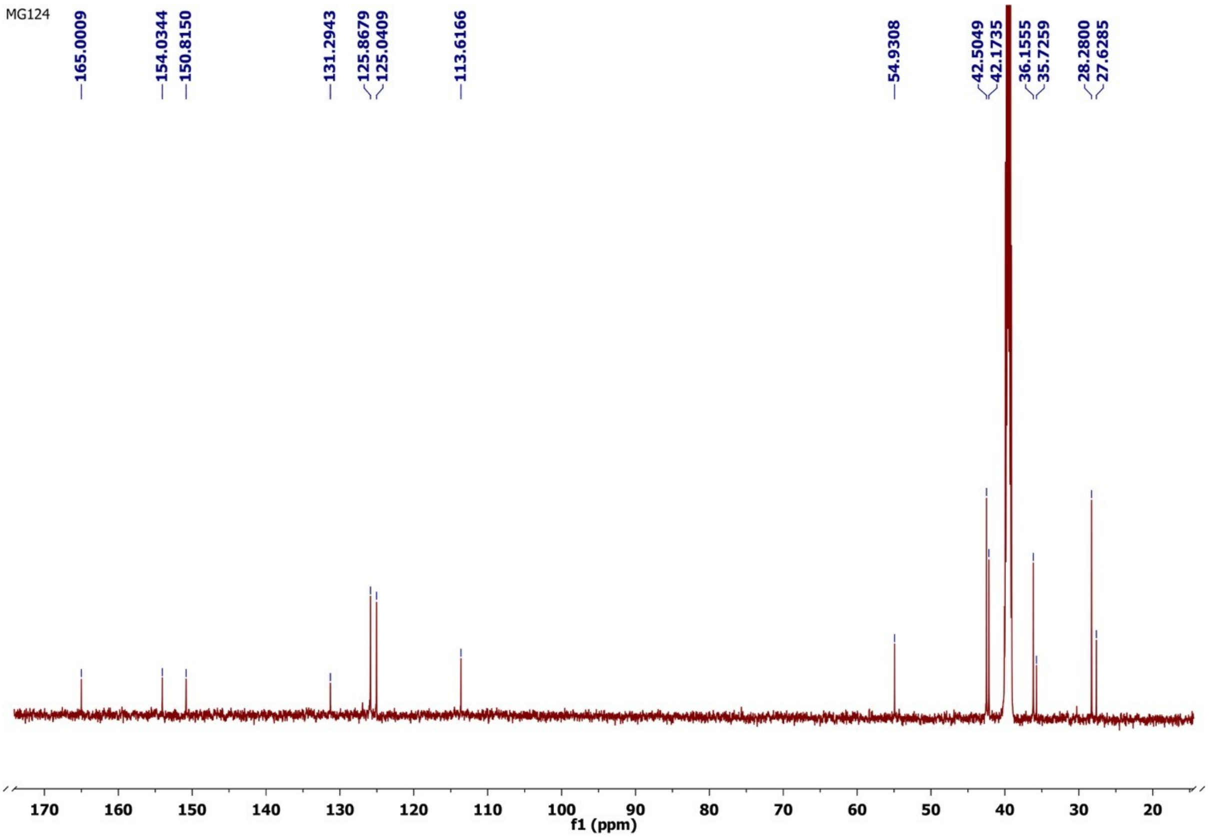

^{13}C NMR (150 MHz, DMSO-d_6) of 2-{4-[4-(1-tricyclo[3.3.1.1^{3,7}]decyl)phenyl]thiazol-2-yl}-*N,N*-dimethylethan-1-amine dihydrochloride (**3d**)

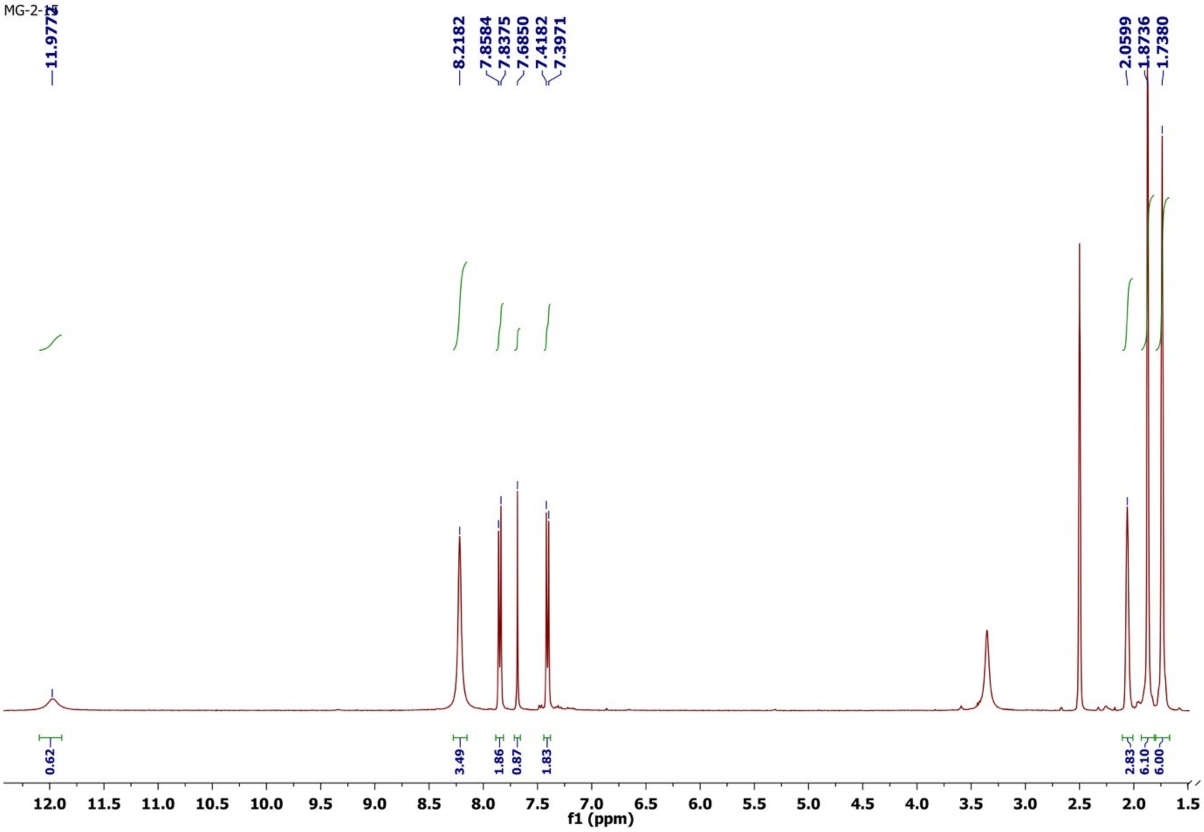

^{1}H NMR (400 MHz, DMSO-d_6) of 1-{2-[4-(1-tricyclo[3.3.1.1^{3,7}]decyl)phenyl]thiazol-4-yl}guanidine (**3e**)

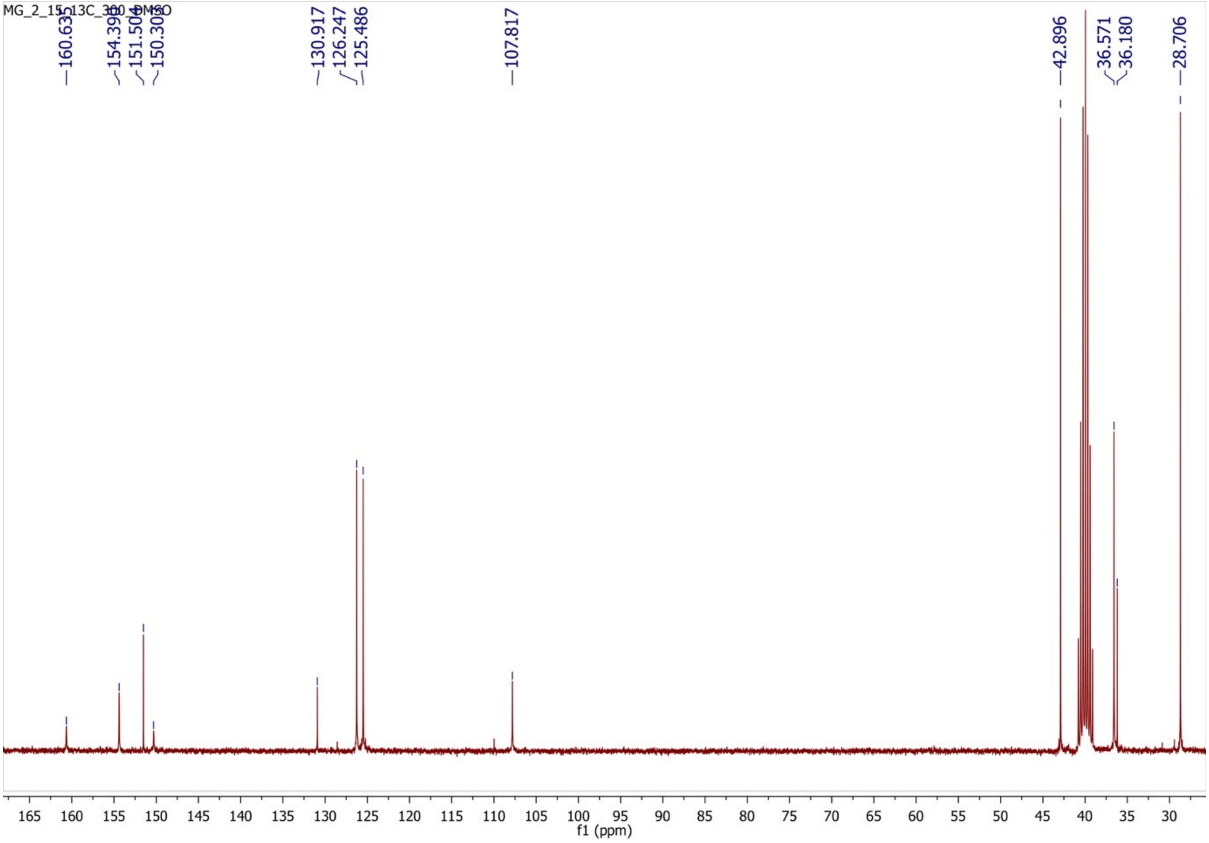

^{13}C NMR (150 MHz, DMSO-d_6) of 1-{4-[4-(1-tricyclo[3.3.1.1^{3,7}]decyl)phenyl]thiazol-2-yl}guanidine (**3e**)

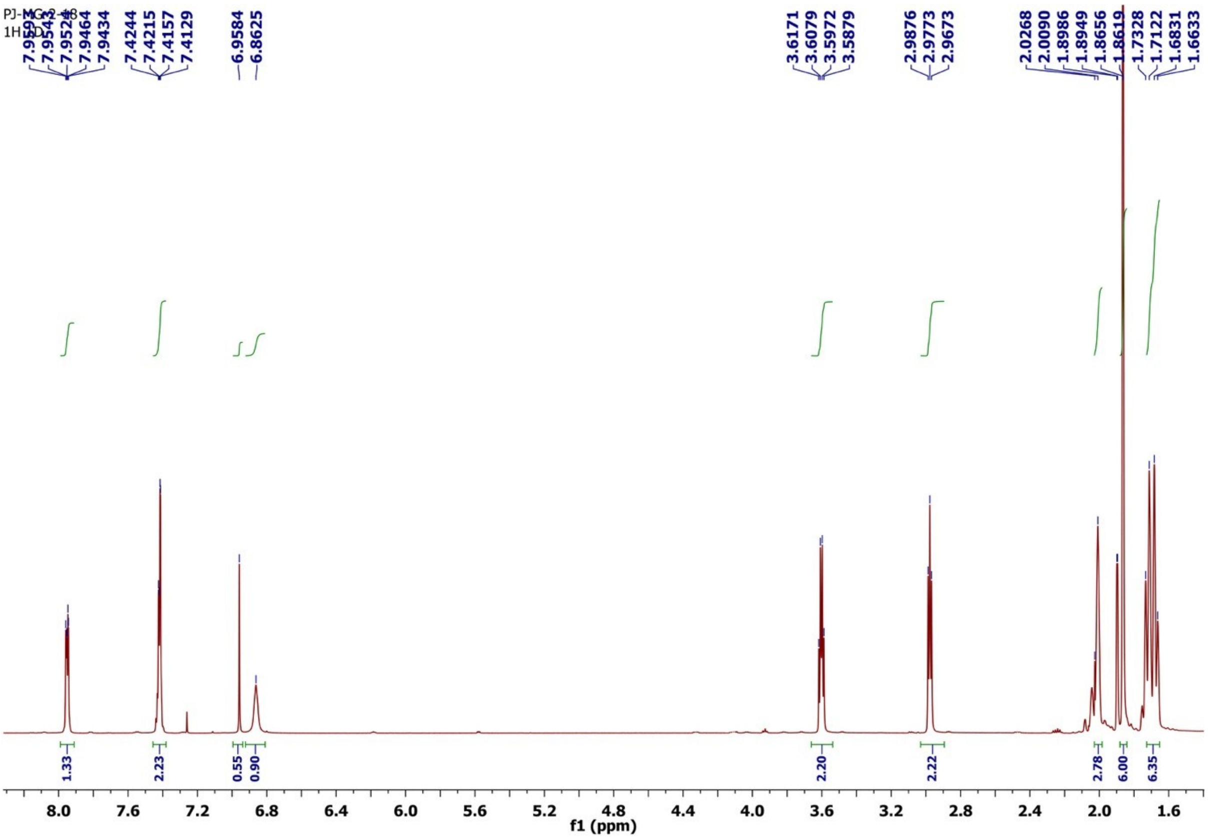

^{1}H NMR (400 MHz, $CDCl_3$) of *N*-[2-(2-Phenylthiazol-4-yl)ethyl](1-tricyclo[3.3.1.1^{3,7}]decane)carboxamide (**4a**)

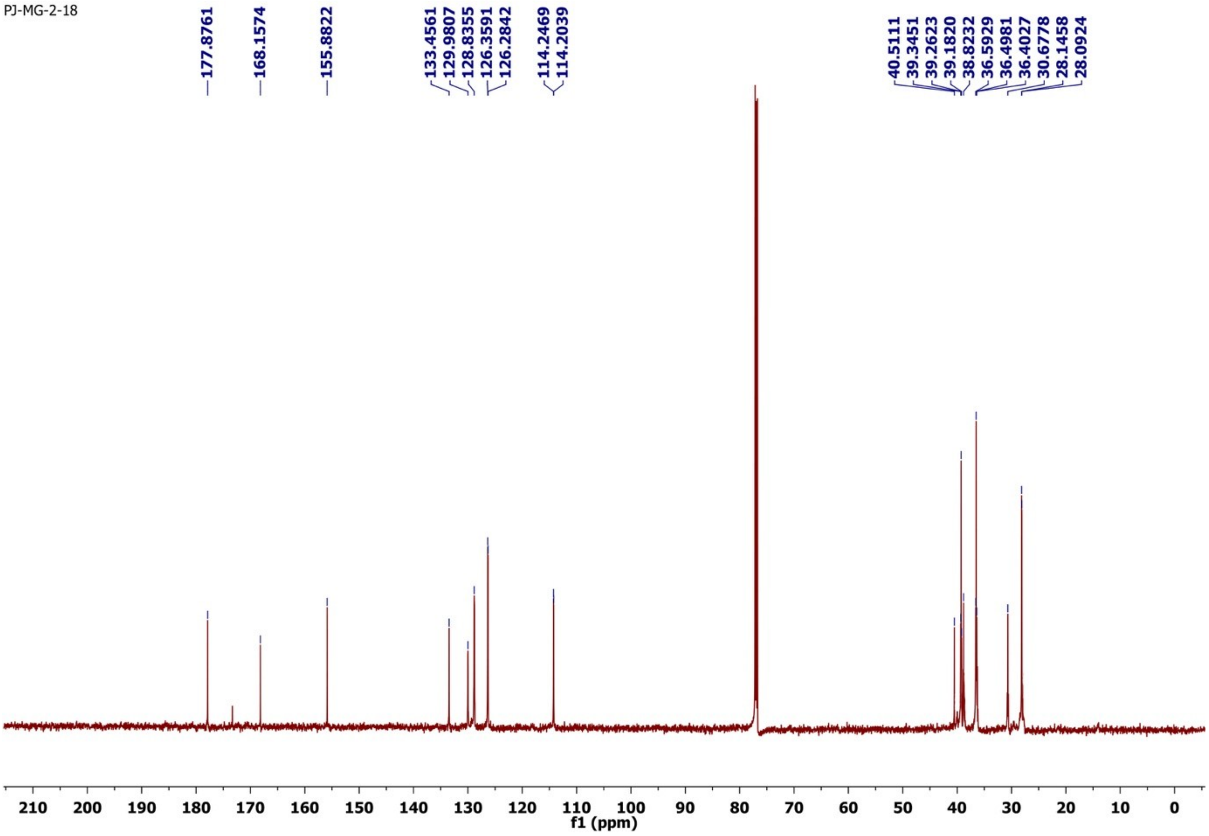

^{13}C NMR (150 MHz, $CDCl_3$) of *N*-[2-(2-phenylthiazol-4-yl)ethyl](1-tricyclo[3.3.1.1^{3,7}]decane)carboxamide (**4a**)

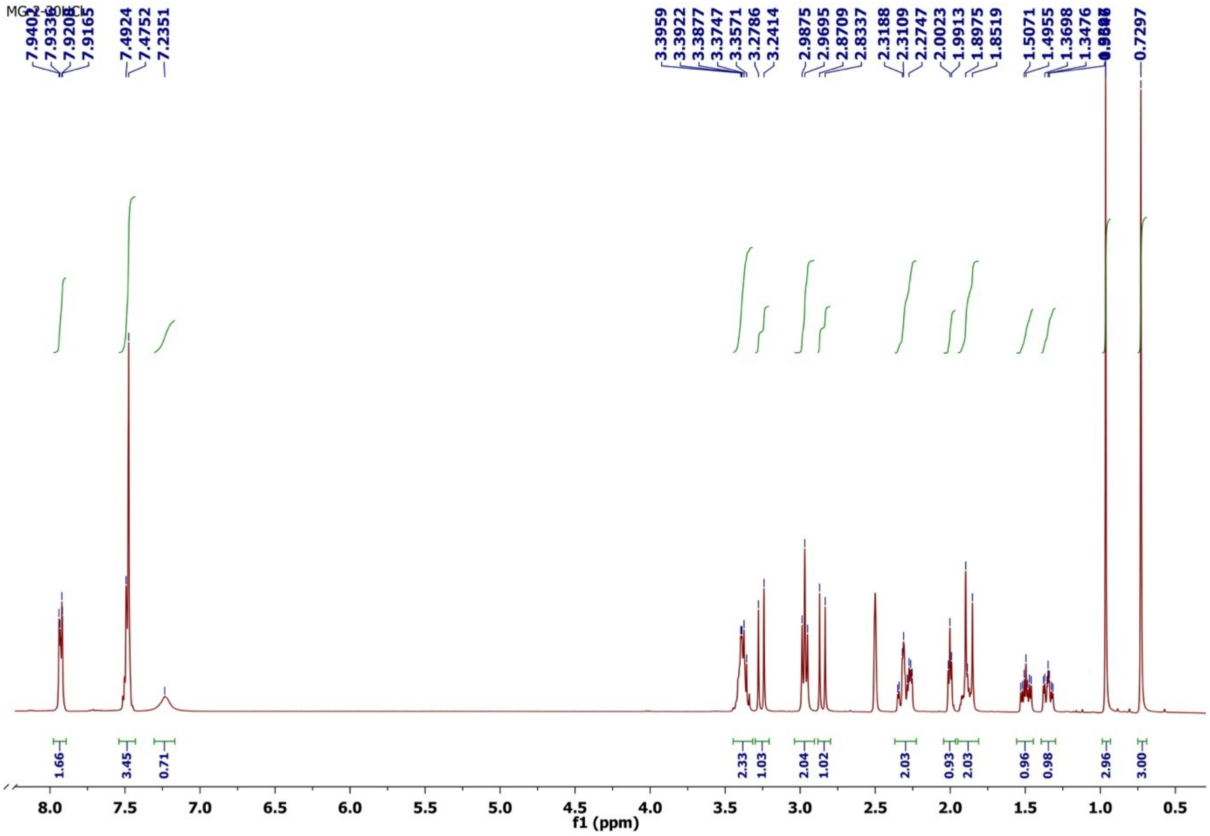

^{1}H NMR (400 MHz, DMSO-d_6) of 1-((1*R*,4*R*)-7,7-Dimethyl-2-oxobicyclo[2.2.1]hept-1-yl)-*N*-(2-(2-phenylthiazol-4-yl)ethyl)methanesulfonamide hydrochloride (**4b**)

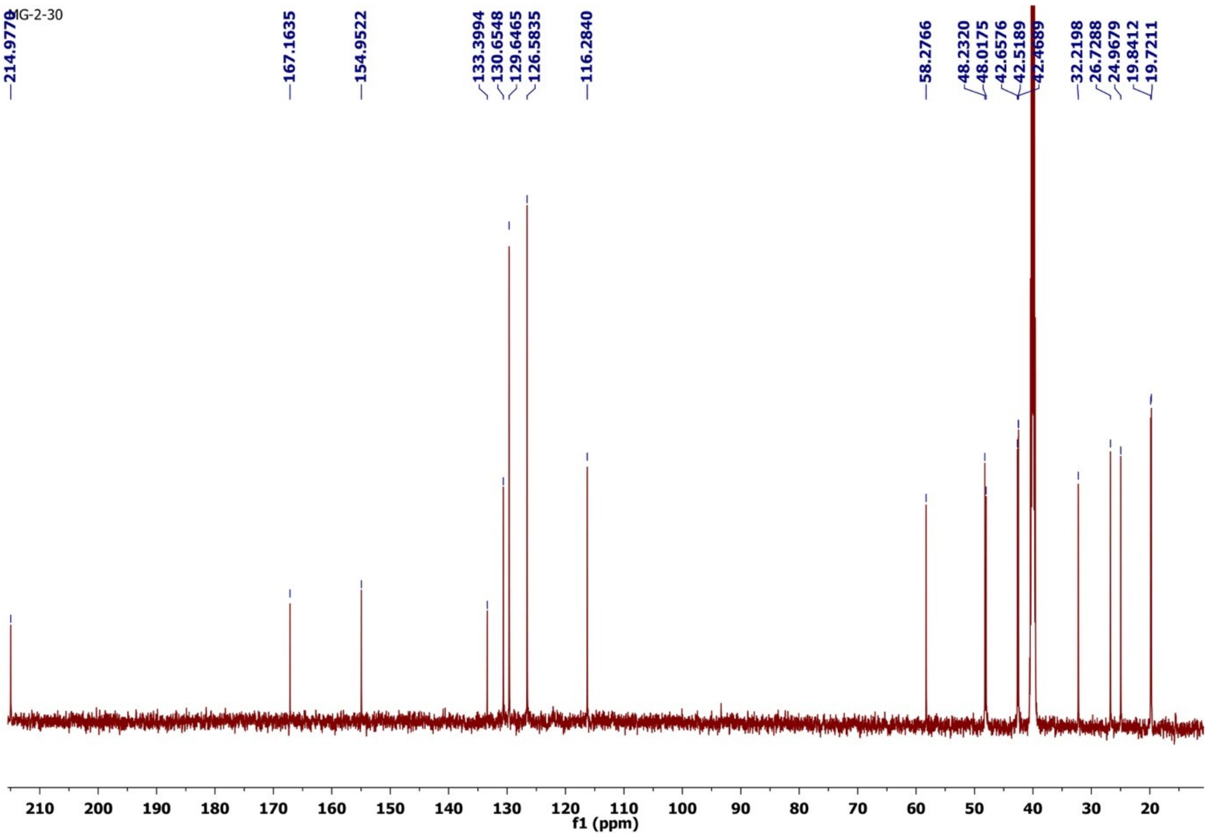

^{13}C NMR (150 MHz, DMSO-d_6) of 1-((1*R*,4*R*)-7,7-Dimethyl-2-oxobicyclo[2.2.1]hept-1-yl)-*N*-(2-(2-phenylthiazol-4-yl)ethyl)methanesulfonamide hydrochloride (**4b**)

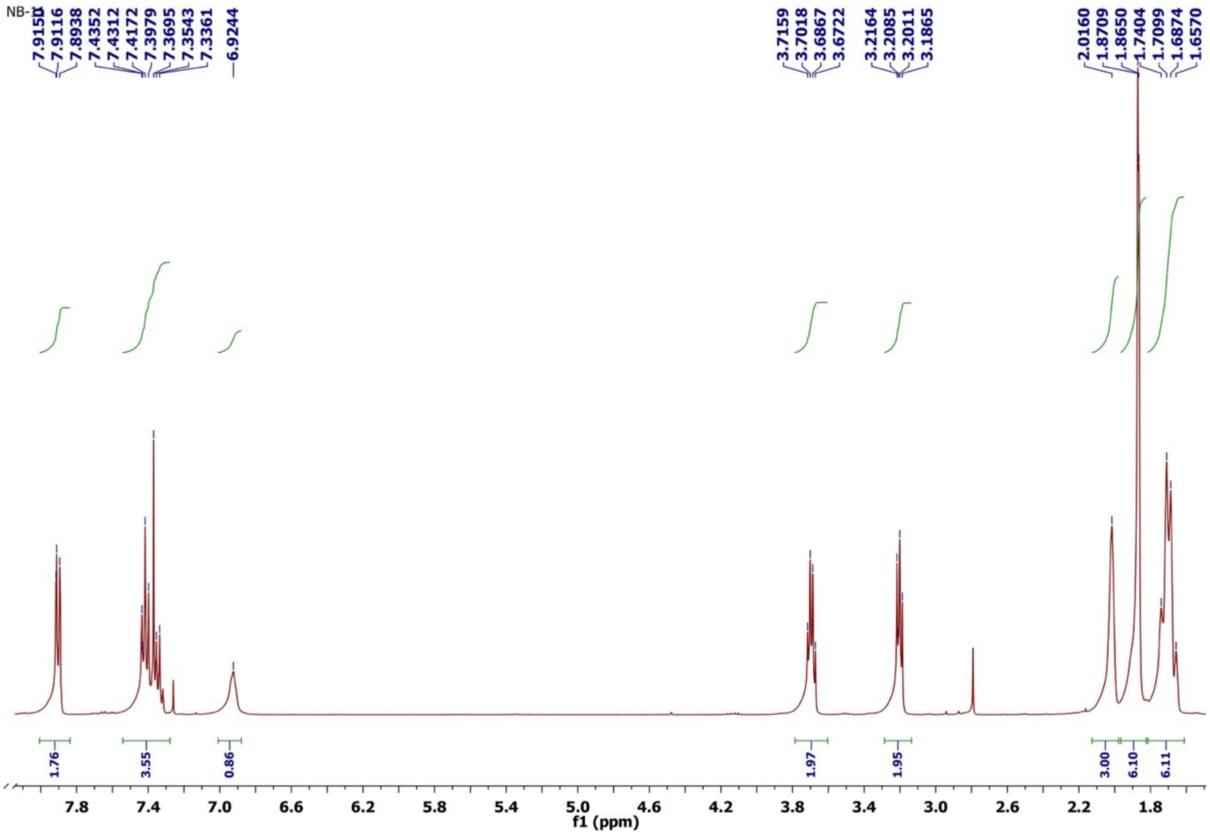

^{1}H NMR (400 MHz, $CDCl_3$) of *N*-[2-(4-Phenylthiazol-2-yl)ethyl](1-tricyclo[3.3.1.1^{3,7}]decanecarboxamide) (**4c**)

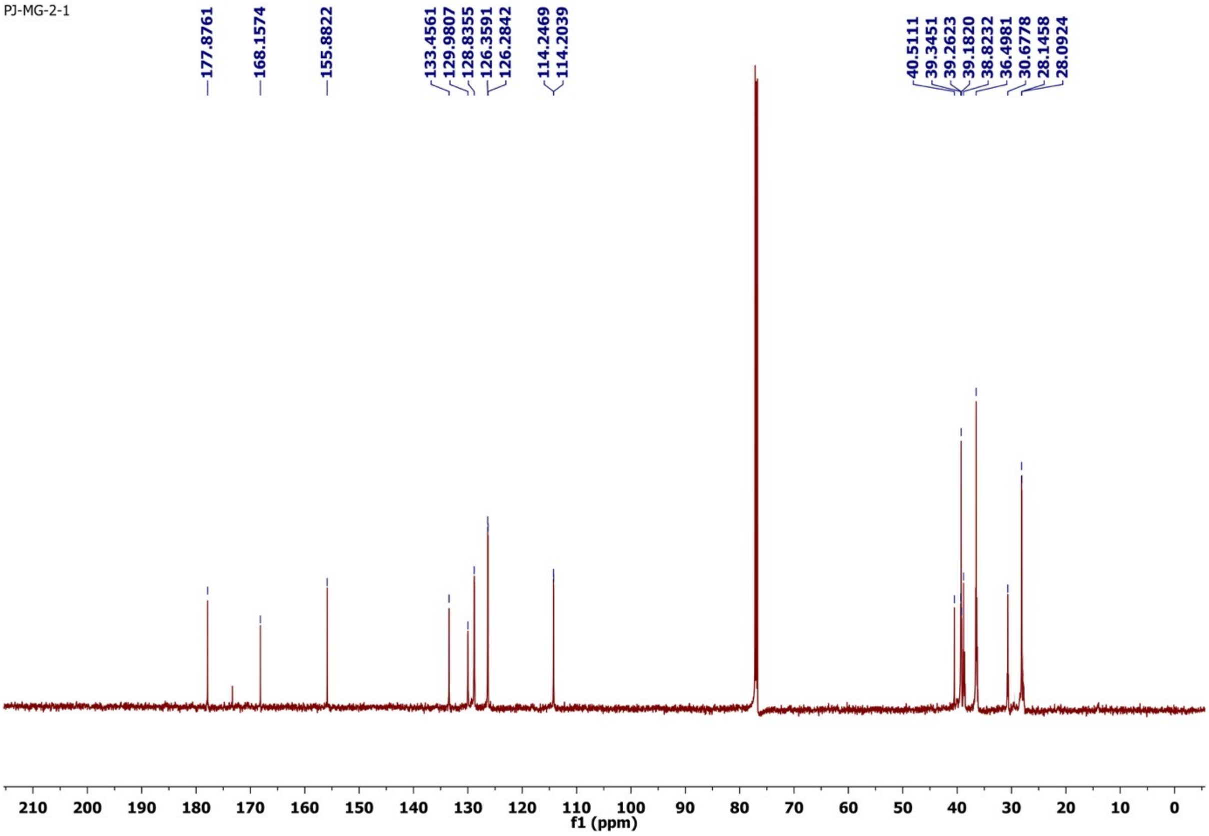

^{13}C NMR (150 Hz, $CDCl_3$) of *N*-[2-(4-Phenylthiazol-2-yl)ethyl](1-tricyclo[3.3.1.1^{3,7}]decanecarboxamide) (**4c**)

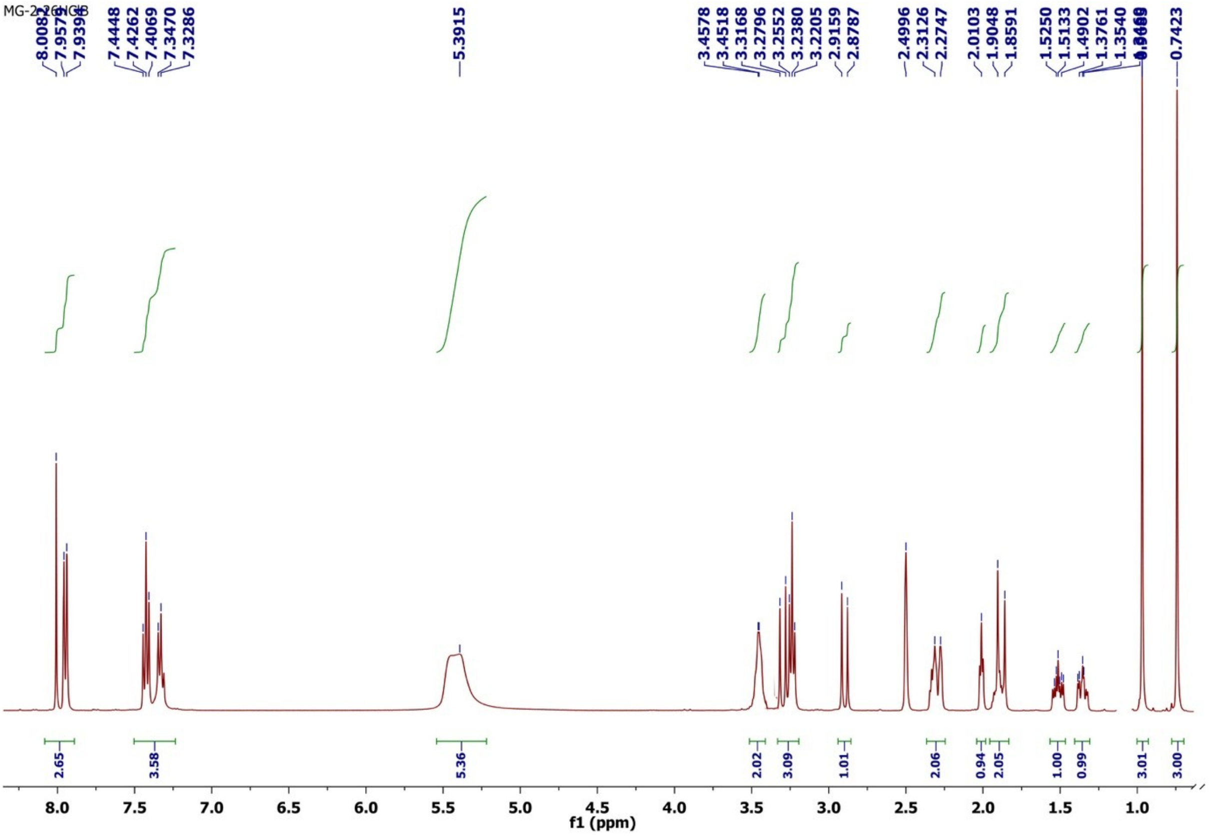

^{1}H NMR (400 MHz, DMSO-d_6) of 1-((*1R,4R*)-7,7-Dimethyl-2-oxobicyclo[2.2.1]hept-1-yl)-*N*-(2-(4-phenylthiazol-2-yl)ethyl)methanesulfonamide hydrochloride (**4d**)

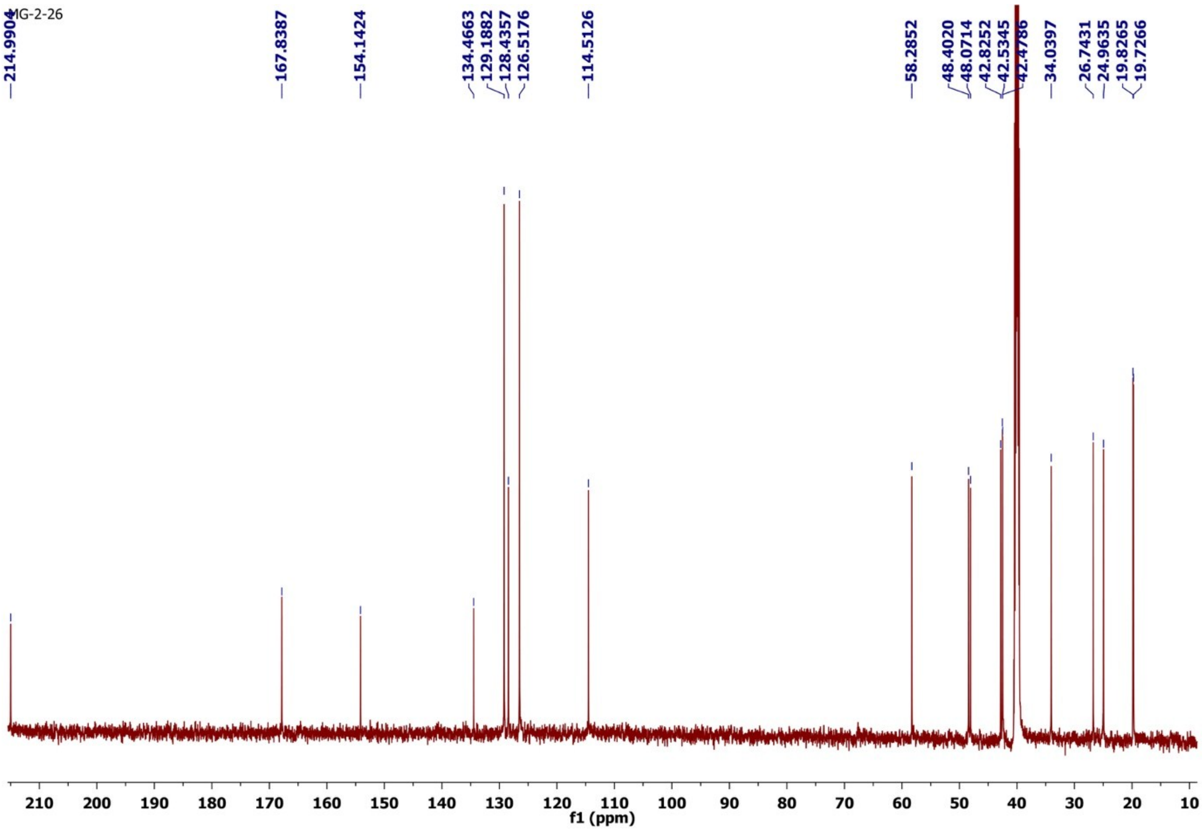

^{13}C NMR (150 Hz, DMSO-d_6) of 1-((*1R,4R*)-7,7-Dimethyl-2-oxobicyclo[2.2.1]hept-1-yl)-*N*-(2-(4-phenylthiazol-2-yl)ethyl)methanesulfonamide hydrochloride (**4d**)
